# Supplementary material for: eHBB: a randomised controlled trial of virtual reality or video for neonatal resuscitation refresher training in healthcare workers in resource-scarce settings
Source: BMJ Open. 2021 Aug 24;11(8):e048506. doi: 10.1136/bmjopen-2020-048506 (PMC8390148; doi:10.1136/bmjopen-2020-048506)
Supplement: Supplementary data [file bmjopen-2020-048506supp001.pdf]

BMJ Publishing Group Limited (BMJ) disclaims all liability and responsibility arising from any reliance placed on this supplemental material which has been supplied by the author(s)

SUPPLEMENTARY FILE 1

STUDY PROTOCOL

eHBB (Helping Babies Breathe)
+
mHBS (mobile Helping Babies Survive)/DHIS2:

Virtual reality technology and DHIS2 mobile data collection to improve newborn healthcare delivery in low and middle income countries

Study Investigators

Principal Investigators

Dr. Rachel A. Umoren
Associate Professor of Paediatrics, University of Washington School of Medicine, Seattle, Washington, U.S.A.

Dr. Sherri Bucher
Associate Research Professor of Paediatrics, Indiana University School of Medicine, Indianapolis, Indiana, U.S.A.

Site PI (Kenya)
Prof. Fabian Esamai
Professor of Paediatrics, Moi University; Principal, Alupe University College, Kenya

Site PI (Nigeria)
Prof. Chinyere V. Ezeaka
Professor of Paediatrics, College of Medicine, University of Lagos. Head of the Perinatology-Neonatology Division, Lagos University Teaching Hospital. Idi-araba, Lagos, Nigeria.

Co-Investigators
Dr. Ime Asangansi
eHealth4everyone, Abuja, Nigeria
Dr. Brian Bresnahan
University of Washington, Seattle, Washington, U.S.A.
Mr. Daniel Hippe
University of Washington, Seattle, Washington, U.S.A.
Dr. Chris Paton
LIFE Project, Oxford University, Oxford, U.K.
Dr. Saptarshi Purkayastha
Indiana University, Indianapolis, Indiana, U.S.A.

Acknowledgments:
We would like to acknowledge the senior administrative and nursing staff at healthcare facilities that participated in the study. We acknowledge the input of Dr. Susan Niermeyer, Dr. Michael Visik and Dr. David Bolnick in developing the HBB 2nd edition video. We acknowledge the members of the University of Oxford LIFE Project who assisted in development of the eHBB VR application: Prof. Michael English, Dr. Niall Winters, Dr. Hilary Edgcombe, Mr. Jakob Rossner, Ms. Naomi Muinga (KEMRI-Wellcome Trust, Kenya) and the staff at National Hospital Abuja who assisted with eHBB VR testing: Dr. Amsa Mairami, Dr. Fatima Mairami, Dr. Adekunle Otuneye, Dr. Lamidi Audu, and Dr. Mariya Mukhtar-Yola. Research assistants were provided with standardized Helping Babies Breathe 2nd Edition Facilitator training by Mr. Sammy O. Barasa and Mr. Geoffrey Mwai, of the Kenya Helping Babies Survive Master Trainer Corps. We would also like to acknowledge the study team members and collaborators who provided administrative, technical, and other support for the study: Mr. Alex McGee, Dr. Iretiola Fajolu, Dr. Beatrice Ezenwa, Dr. Felicitas Okwako, Mr. John Feltner, Ms. Mary Nafula, Ms. Annet Musale, Ms. Olubukola Olawuyi, Ms. Christianah Adeboboye, Ms. Oyin Akinrinola, Mr. Dillon Afenir, Mr. Bhavani Agnikula Kshatriya, Dr. Benjamin Al-Haddad, Mr. Iman Asangansi, Mr. Prem Avanigadda, Mr. Ariyo Ayokunle, Mr. Uchechukwu Chinedu, Mr. Emeka Chukwu, Mr. Chima Chukwudi, Ms. Bailey Clopp, Mr. Domnan Diretnan, Mr. Aniekan Ebito, Ms. Sakina Ginary, Ms. Chioma Ginikanwa, Mr. Oystein Gomo, Mr. Manasseh Mmadu, Ms. Ajayi Motunrayo, Mr. Alvin Ogbonna, Mr. Kevin Otieno, Ms. Shruti Patel, Ms. Amanda Stiffler, Dr. Amy Thompson; *in memorium,* Dr. Charles Spiekerman and Ms. Elisabeth Meyers.

Table of Contents

1. Study Overview 5
2. Background 6
3. Study Objective 7
4. Study Procedures 7
4.1 Study Participants 7
4.2 Study Facilities 7
4.3 Inclusion Criteria 7
4.4 Exclusion Criteria 8
4.5 Recruitment 8
4.6 Informed Consent 8
4.7 Randomization/allocation procedure 8
4.8 Outcome Measures 8
5. Study Interventions 9
5.1 Pre-course interventions 9
5.2 Pre-course assessments 10
5.3 Helping Babies Breathe 2nd Edition Course 10
5.4 Immediate post-course assessments 12
5.5 Follow up assessments 12
6. Study Incentives 12
7. Data management 13
8. Data analysis 13
8.1 Sample size calculations 13
9. Qualitative studies 14
10. References 15
APPENDICES 17
Appendix 1. Abbreviations/Definition of Terms 17
Appendix 2. Study Diagram 18
Appendix 3. Recruitment Information 19
Appendix 4. Consent Form 20
Appendix 5. Participant Surveys 24
Appendix 6. HBB Knowledge Check 31

Appendix 7. Simulation Checklists 34

eHBB + mHBS/DHIS2: Virtual reality technology and DHIS2 mobile data collection to improve newborn healthcare delivery in low and middle income countries

1. Study Overview

Neonatal mortality, or death among newborns 0-28 days, now accounts for approximately 44% of global under 5-years child mortality.[1] Each year, there are 2.65 million stillbirths,[2,3] and nearly 2.8 million newborn deaths, most of which are preventable. The majority of these deaths occur in low- and middle-income countries (LMICs). Intrapartum asphyxia is one of the three leading causes of neonatal mortality, and also underlies high rates of newborn morbidity[4]. Training in neonatal resuscitation (NR) reduces asphyxia-related newborn mortality and morbidity, but there are barriers to the effective implementation of NR programs in LMICs, which are exacerbated by gaps in data collection of key indicators and outcomes.

However, the high penetration of mobile smartphones and cellular network connectivity in urban areas, along with new mobile graphics processing capabilities, makes innovative simulation training, including mobile-phone based virtual reality (VR), potentially feasible even in LMICs. Mobile VR simulations can be engaged at the learner's convenience, on their own smartphone, with game-based automated feedback that is ideal for episodic learning. Mobile VR training provides an opportunity to address challenges related to (a) maintenance of neonatal resuscitation knowledge and skills over time; (b) inconsistent migration of neonatal resuscitation skills into actual clinical practice; and (c) lack of a standardized dissemination to support neonatal resuscitation in LMICs.

We propose to use mobile VR simulations and a DHIS2-integrated mobile data collection platform as a tool to leapfrog over the current challenges to obtaining and maintaining NR knowledge and skills among health care providers (HCPs) in LMICs including lack of infrastructure, staff shortages, and high rates of staff turnover. Our hypotheses are that mobile phone-based VR training and digital data collection will provide: 1) effective training in neonatal resuscitation 2) an easy method to maintain skills, and 3) a less costly option of neonatal resuscitation training than current methods.

Methodology: To test these hypotheses we will conduct a randomized controlled trial of the VR neonatal resuscitation module in healthcare providers in Nigeria and Kenya to assess the impact of module on neonatal resuscitation performance.

Outcome variables: We will assess healthcare provider knowledge and performance of neonatal resuscitation in educational (simulation) and clinical settings and compare this performance across study groups.

2. BACKGROUND

Helping Babies Breathe (HBB) is a neonatal resuscitation training program for health care providers (HCPs) in LMICs. Since its global launch in June 2010, HBB has rolled out in over 80 countries worldwide. Helping Babies Breathe (HBB) training is conducted using a dissemination model called "training of trainers" (TOTs), which involves one to three days of in-person training of future trainers or providers of neonatal resuscitation through the use of lectures, small group learning, hands-on practice among paired learners using low-tech manikins, and evaluation of participants' knowledge and skills using a multiple choice questionnaire, skills checks, and objective structured clinical examinations. However, field studies demonstrate that initial HBB training alone is insufficient for transfer of skills to clinical practice.[5-7] In the absence of continued mentoring and a supportive environment after the course, there is often a failure, among HBB providers, to consolidate and maintain the required knowledge, skills, and performance competencies which are required in order to perform high-quality neonatal resuscitation (NR) during actual clinical practice.[8,9]

Access to mobile phones, including smart phones and devices, has become nearly ubiquitous in LMICs allowing the introduction of e-learning that increases availability, scalability, flexibility and efficiency while decreasing marginal costs for training[10,11]. Evidence shows that e-learning is generally as effective as traditional teaching methods[12]. Under the right conditions, e-learning has the ability to promote innovations in design and distribution[13,14], including interactive learning, problem based learning, and standardized feedback[14-16]. Computer-based simulations, a subset of e-learning strategies, are increasingly used for self-directed learning before, or after attending an in-person course, e.g. HeartCode® ACLS, HeartCode® PALS and NRP eSim® programs.

Mobile VR simulations can be delivered on mobile phones as *screen-based* experiences transformed into *immersive* experiences with the use of a low-cost VR headset. Typically, they are brief, engaging, interactive, experiences that are relevant to clinical practice with the goal of fostering experiential learning that transforms attitudes and behaviors. VR simulations use a strategy of deliberate practice similar to the Ericsson's deliberate practice paradigm,[17] in which learners deliberately practice their skills with expert coaching and feedback until they are able to perform the skills with minimal coaching. In games, automated feedback is tailored to the learner's performance to ensure that practice occurs until the learner achieves the skills needed to move on to the next level of difficulty[18].

Integrating mobile VR training and digital data collection with neonatal resuscitation training in LMICs will address the challenges of (a) maintenance of knowledge and skills over time; (b) inconsistent migration of competencies into actual clinical practice; and (c) lack of a standardized, integrated, bi-directional data collection and information dissemination platform to support training in LMICS. The use of mobile VR simulations in HBB programs potentially

represents increased access to training, opportunities for self-directed learning, and less costly skill maintenance.

Investigators at the University of Washington have co-developed a virtual Helping Babies Breathe (eHBB) training simulation with Oxford University on the Life-Saving Instruction for Emergencies (LIFE) platform. A game-like approach utilizing a 3D game engine allows the user to experience an immersive and engaging training experience that has been adapted for the proposed eHBB (electronic Helping Babies Breathe) module. eHBB will run on a mobile phone but will also include the capability to work with low-cost VR headsets. In addition, investigators at Indiana University are customizing the mHBS (mobile Helping Babies Survive) application, an expanded version of an existing open-source data collection application mHBB to deliver the eHBB training module. The integrated eHBB and mHBS module will be used to augment initial and refresher training on key NR knowledge, skills and competencies among HCPs in LMICs.

3. Study Objective

The primary objective is to assess the impact of mobile VR simulation used before and after initial NR training on neonatal resuscitation educational indicators and performance outcomes in healthcare providers, in comparison to traditional HBB training.

4. Study Procedures

4.1 Study Participants

Our overall target sample size is 250 participants, but we will recruit up to 280 total participants across two international sites with an estimated 140 participants per site to account for attrition. Potential study participants will be identified from individuals registered to take an in-person HBB course or because they work or are assigned rotations in the labor/delivery wards of participating facilities.

4.2 Study Facilities

Participants will be recruited at study sites in Nigeria and Kenya. Potential study sites currently identified include Lagos University Teaching Hospital and referring facilities: Randle General Hospital, Surulere, and Federal Medical Centre, Ebute-Meta, Lagos Island Maternity Hospital, Lagos Island, Harvey Rd. Health Center, Shomolu General Hospital, Mushin General Hospital, Isolo General Hospital, Regina Mundi Hospital, Mushin, R-Jolad Specialist Hospital, Lagos, Nigeria. These facilities were selected on the basis of delivery volumes; number of nursing staff; presence of a neonatal unit; and/or association with a nursing and/or midwifery training program.

4.3 Inclusion Criteria

All HCPs who provide neonatal resuscitation to inborn or outborn infants and provide study consent. Key stakeholders in healthcare administration and community-based stakeholders will

also be recruited to participate in key informant interviews and focus group discussions.

4.4 Exclusion Criteria

HCPs who have received NR training less than one year before enrollment in the study; individuals who do not provide neonatal resuscitation as part of their duties; or will be unavailable or unwilling to participate in follow-up study activities throughout the 6-month post-initial training period will be excluded.

4.5 Recruitment

Site coordinators or research assistants will identify potential study participants from HCPs working in study facilities that meet eligibility criteria and are enrolled in a study HBB course.

A convenience sample of key stakeholders within Nigeria and Kenya, such as health facility administrators and community-based stakeholders will also be recruited to participate in key informant interviews and focus group discussions. (Appendix 3, Recruitment information)

4.6 Informed Consent

Site coordinators or research assistants will obtain informed consent from potential study participants. Each site is to provide their consent form to each study participant to be signed prior to randomization. (Appendix 4, Consent form).

4.7 Randomization/allocation procedure

Study numbers will be randomly generated, via a computer-generated algorithm for the VR, Educational (NR) video only, or Control (standard HBB TOT) groups. The eHBB study will be using block randomization based on provider experience: novice (no previous HBB training) and non-novice (any previous HBB training) to ensure that recruited participants are evenly distributed into all arms and groups of the study.

4.8 Outcome Measures

The *HBB Knowledge check* is a standardized HBB tool, is an 18-item multiple choice questionnaire (MCQ) which assesses HCP knowledge regarding immediate care after birth, routine care, and basic neonatal resuscitation (Appendix 6, HBB Knowledge Check).

BMV Checklist: The bag-and-mask ventilation skills checklist (BMV) is a standardized HBB tool used to assess HCPs competency on the provision of positive pressure ventilation with a bag-and-mask device (Appendix 7, Simulation Checklists).

Standardized simulations/OSCES: The HBB 2nd edition curriculum has two OSCEs, called OSCE A and OSCE B. These standardized simulations involve a series of time-sensitive tasks which happen in rapid succession and follow the steps of the HBB Action Plan (Appendix 7, Simulation Checklists).

BMJ Publishing Group Limited (BMJ) disclaims all liability and responsibility arising from any reliance placed on this supplemental material which has been supplied by the author(s)

Delivery Checklist: Routine newborn care provided by participating healthcare providers will be observed by research assistants using a standardized checklist.

5. Study Interventions

5.1 Pre-course interventions

Site coordinators or research assistants will provide links and airtime to download the mHBS/DHIS-2 application to their phone or a study device.

Pre-survey: The baseline HBB knowledge check and a demographic survey with questions regarding age, gender, area on duty, years of experience, previous health care, and NR training, time elapsed since NR previous training, experience/comfort with using computers, experience/comfort with video games, smartphone or tablet device ownership, will be administered through the mHBS application.

Completion of the pre-survey and baseline HBB knowledge check will "unlock" the study group assignment.

Figure 2. Study Timeline

	Baseline assessment	Pre-course intervention	Pre-course assessment	Immediate post-course assessment	1 month post-course assessment	3 month post-course assessment	6 month post-course assessment
**VR Group**	Knowledge test	eHBB (VR) +Digital HBB manual	Knowledge test, simulation performance	Knowledge test, simulation performance	Knowledge test, simulation performance	Knowledge test, simulation performance	Knowledge test, simulation performance
					VR + LDHF manikin practice		
**Video Group**	Knowledge test	HBB video +Digital HBB manual	Knowledge test, simulation performance	Knowledge test, simulation performance	Knowledge test, simulation performance	Knowledge test, simulation performance	Knowledge test, simulation performance
					NR Video + LDHF manikin practice		
**Control Group**	Knowledge test	Digital HBB manual	Knowledge test, simulation performance	Knowledge test, simulation performance	Knowledge test, simulation performance	Knowledge test, simulation performance	Knowledge test, simulation performance
					LDHF manikin practice only		
Baseline Clinical Observations		Post-training clinical observations					

Legend: HBB: *Helping Babies Breathe*
NR: Neonatal resuscitation;
VR: Virtual reality;
LDHF: Low-dose high frequency

Umoren R, *et al. BMJ Open* 2022; 11:e048506. doi: 10.1136/bmjopen-2020-048506

BMJ Publishing Group Limited (BMJ) disclaims all liability and responsibility arising from any reliance placed on this supplemental material which has been supplied by the author(s)

Study group assignment: Participants will be given access to study materials based on their group assignment of VR (eHBB), NR video or Control groups for review before their in-person HBB course. All groups will receive digital versions of the HBB manual accessible through the mHBS platform.

5.2 Pre-course assessments

During the pre-course period, study personnel will visit study facilities on a weekly basis (or more frequently) to observe delivery practices, number of deliveries, and will conduct a needs assessment on the preparedness of study facility and personnel to provide neonatal resuscitation.

- All study facilities will be provided with resuscitation kits as needed, based on the initial HBB equipment and resource mapping, which will be conducted during the clinical observations baseline period, before in-person HBB training courses.

The research assistant will also perform baseline knowledge tests, bag-mask ventilation (BMV) skills and standardized simulation performance assessments in all participants using the NeoNatalie Advanced Simulator (Figure 3).

Figure 3. Neonatalie Advanced Manikin (Laerdal)

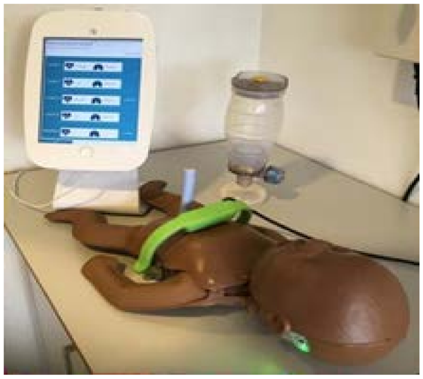

Participant performance during the simulation will be tracked using the Simulation (BMV and OSCE) Checklists by the research assistant and by the NeoNatalie Advanced simulator with standardized performance-based feedback provided to each participant. The manikin stores training data in a database that can be accessed remotely. When feasible, research assistants doing the performance and clinical observations will be blinded to the allocation of the HCPs whom they are assessing.

5.3 Helping Babies Breathe 2nd Edition Course

All study participants will complete a traditional in-person HBB Second Edition provider training course using a NeoNatalie low-fidelity manikin training kit and provider training materials. All participants will receive course attendance fees and a HBB 2nd edition course completion certificate.

HBB corner: All facilities will be encouraged to set up a HBB corner for manikin-based low-dose, high-frequency practice per current HBB program recommendations. All participants in all groups will be encouraged to perform low dose, high frequency (LDHF) manikin-based practice at facility-based HBB corners per current HBB recommendations. The frequency with which

participants interact with the manikins will be tracked using self-report on the mHBS app and paper logs at the HBB corners, and with trackers if feasible.

Figure 1. Study Diagram

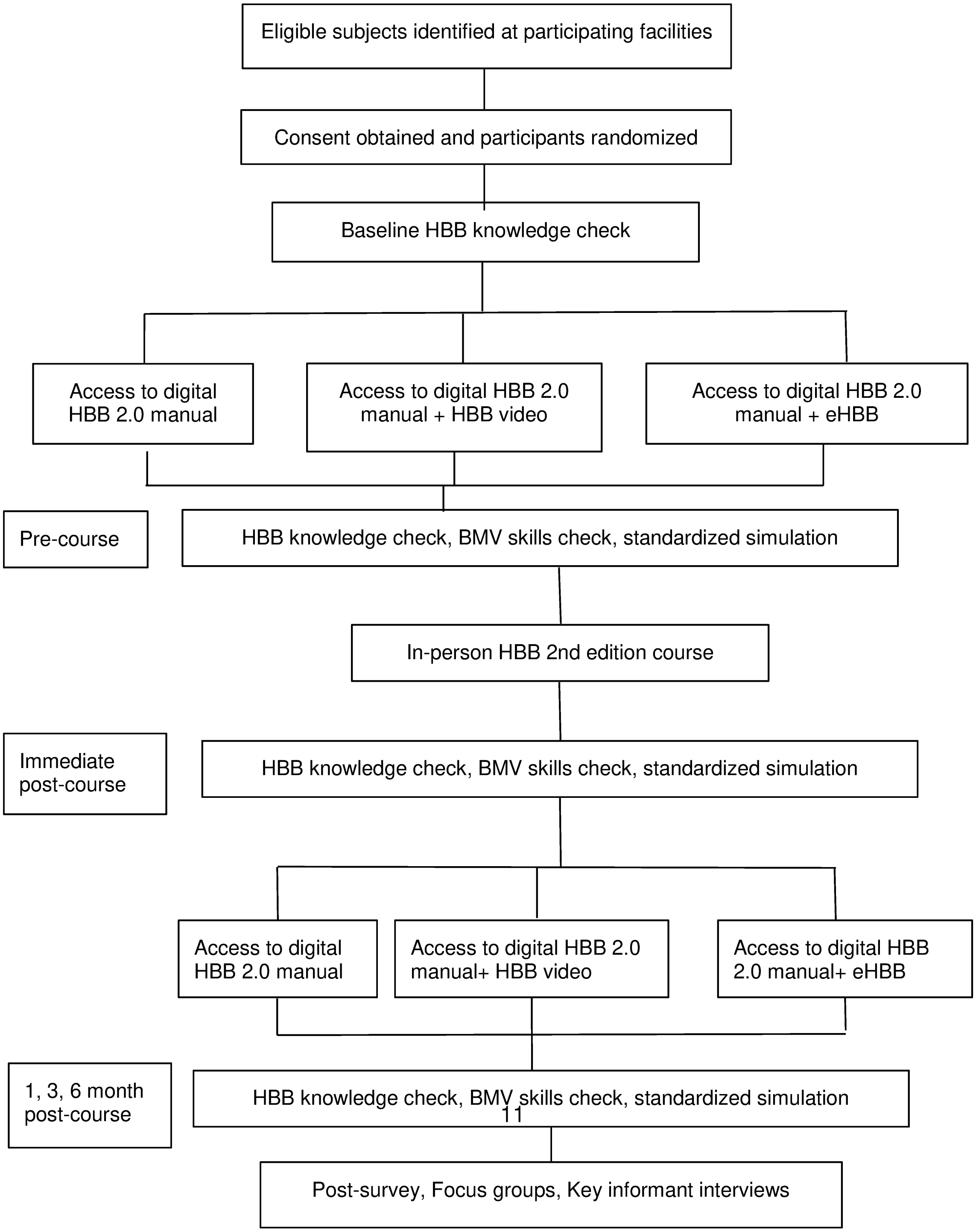

5.4 Immediate post-course assessments

Immediately after the HBB course, the research assistant will perform baseline knowledge tests, bag-mask ventilation (BMV) skills and standardized simulation performance assessments) in all participants using the NeoNatalie Advanced Simulator to conduct standardized simulation performance assessments.

Participant performance during the simulation will be tracked using the BMV and OSCE Checklists by the research assistant and by the NeoNatalie Advanced simulator with standardized performance-based feedback provided to each participant. The manikin stores training data in a database that can be accessed remotely. Where feasible, research assistants doing the performance and clinical observations will be blinded to the allocation of the HCPs whom they are assessing.

5.5 Follow up assessments

Participants will have ongoing access to the mHBS platform and will be sent reminders at 1, 3, and 6 months after initial NR training to take a follow-up NR knowledge test and to again participate in standardized performance simulations of bag-mask ventilation (BMV) skills and standardized simulation performance assessments conducted by research assistants who will visit their hospital facility with the NeoNatalie Advanced Simulator.

To understand the impact of educational strategies on clinical practice, clinical observations of routine care provided by participating healthcare providers will be conducted by research assistants in participating facilities using a standardized Delivery Checklist. This includes data on newborn care at the time of delivery and resuscitation (if applicable). See attached checklist. We will also gather data regarding capacity for provision of NR within facilities (e.g., number of deliveries, availability of trained staff and NR equipment), presence/absence of HBB practice corners, and rates of NR training among HCPs). We will review delivery outcomes if available from existing databases such as the Neonatal Registry before and after the HBB training. The Neonatal Registry data will be deidentified by a staff member who is not a member of the study team before release. When possible, resuscitation debriefs will be encouraged using the resuscitation debrief form and the perinatal death audit form will be used to identify the cause of death when not specified (see attached). No identifiable patient data will be stored and study facilities and participants will be identified by only by study code number.

At the end of the 6-month follow up period, participants will be asked to complete a feedback survey, designed to capture metrics related to the accessibility and utility of VR for neonatal resuscitation initial and refresher training.

6. Study Incentives

All active study participants will be provided with travel (if applicable), course attendance fees and air-time to facilitate upload of study data to a secure study database during the study follow-up period. Automated reminders will be sent one week before, on the due date, and one week

Supplemental material BMJ Publishing Group Limited (BMJ) disclaims all liability and responsibility arising from any reliance placed on this supplemental material which has been supplied by the author(s) BMJ Open

after the activity is due. After the third automated reminder, two attempts will be made by the study investigators to contact the study participant using contact information provided on enrollment. If no contact is made, the study participant will be deemed inactive.

7. DATA MANAGEMENT

Neonatalie Advanced Manikin data collected during simulation sessions will be identified by study ID only. *HBB Knowledge check, BMV and OSCE Checklists* data will be entered into mHBS and RedCap. Data from paper records will be entered into RedCap after each session. Electronic copies of study data will be stored on a local password protected study computer and in a secure study database. The hard copy of all documents should be filed in a locked cabinet in the study office.

8. DATA ANALYSIS

Demographic data will be compared between VR, NR video, and control groups and assessed for group to group differences. Pass/Fail rates on the study evaluations: Knowledge test, bag-and-mask ventilation skills assessment (BMV), and standardized simulations of routine care and initial resuscitation (OSCE A) and prolonged resuscitation (OSCE B) done at the following time periods: Baseline, Pre-course, Immediate post-course, and at 1 month, 3 months, and 6 months in the follow up period will be calculated and compared between groups. The primary endpoint is the 6-month post-baseline evaluation. We will perform an analysis of covariance with each six-month score regressed on the baseline score of each subject and compared among the three groups.

Two post-hoc statistical tests will be performed to ascertain possible statistically significant differences between VR and Video groups with the Control group at each time point. A secondary analysis will take full advantage of the longitudinal nature of the data (see Figure 9). Here, we posit a structural response model where skills will be initially acquired uniformly by all subjects (at the immediate post-course assessment after face-to-face training) but retention of these skills will dissipate rapidly over time in the Control group, but less precipitously in the other two groups, with the least deterioration of knowledge and skills in the VR group. The statistical modeling approach in this case will be through a change-point model with random intercept and slope. The main statistical test will be the assessment of the statistical significance of the interaction between group and time, during the second period (i.e., after the change or inflection point in the curve).

8.1 SAMPLE SIZE CALCULATIONS

We hypothesize that there will be a minimum 20% difference in the proportion of subjects who pass OSCE A and OSCE B, via a one-sided test at the 6-month evaluation. The desired power is 80% taking into account that two comparisons (VR vs. Controls and Video vs. Controls) will be carried out with the hypothesis that the proportions of subjects in the VR group will demonstrate performance metrics that are higher (by at least 20%) than the control group.

The alpha level has been halved so as not to inflate the type-1 error rate (of 5% overall) so 83 participants per group or a total of 249 participants will be sufficient to detect this difference under all the scenarios considered here. However, to account for study dropout, we will recruit up to 280 participants across the two country sites. We expect that the power generated by the ANCOVA procedure will be even higher as well as the power generated by the longitudinal (secondary) change-point analyses will be much more sensitive (powerful) to detect differences in each pairwise comparison. Thus the proposed sample size will likely be able to detect difference in skill retention with very high power.

9. QUALITATIVE STUDIES

This will consist of focus groups and key informant interviews led by an experienced facilitator utilizing semi-structured questionnaires. We will conduct 4-6 focus groups each consisting of 6-8 study participants and up to 15-20 key informant interviews among NR trained HCPs enrolled in the study and a convenience sample of key stakeholders within Nigeria such as health facility administrators and community-based stakeholders.

Domains addressed will include: what exposure to VR do healthcare providers have currently and what are their thoughts about learning from VR? How and when do healthcare providers use VR and what are their perspectives on it? What are the barriers and facilitators to using VR for initial and refresher training?

Sessions will be digitally recorded and transcribed. The data will be organized using qualitative analysis software. Qualitative data will be analyzed by two study investigators experienced in thematic analysis. Investigators will independently review all FGD transcripts to identify initial themes then work together to build consensus on all major themes. They will then independently code all transcripts through an iterative process using thematic content analysis[19]. As new codes are identified, the coding scheme will be refined using the grounded theory constant comparative method[20].

Investigators will meet regularly to compare and discuss codes, resolve disagreements, and come to consensus on discordantly coded data. Saturation will be achieved after no new themes emerge from the data. Codes within and across transcripts will be compared and synthesized into overarching themes that reflect the perspectives of healthcare providers on the use of technology and VR for training. A random sample of transcripts will be double coded to ensure agreement between coders. The data will be used to prepare a report on the facilitators and barriers to VR training for NR initial and refresher training in LMICs from the perspective of participants and stakeholders. The results will be shared with a subset of the participants for verification.

10. REFERENCES

1. Chou D, Daelmans B, Jolivet RR, Kinney M, Say L. Ending preventable maternal and newborn mortality and stillbirths. *BMJ.* 2015;351.
2. Sharma G, Mathai M, Dickson K, et al. Quality care during labour and birth: a multi-country analysis of health system bottlenecks and potential solutions. *BMC Pregnancy Childbirth.* 2015;15(Suppl 2):S2.
3. Enweronu-Laryea C, Dickson K, Moxon S, et al. Basic newborn care and neonatal resuscitation: a multi-country analysis of health system bottlenecks and potential solutions. *BMC Pregnancy Childbirth.* 2015;15(Suppl 2):S4.
4. Lawn JE, Blencowe H, Oza S, et al. Every Newborn: progress, priorities, and potential beyond survival. *The Lancet.* 2014;384(9938):189-205.
5. Ersdal H, Singhal N. Resuscitation in resource-limited settings. Paper presented at: Seminars in Fetal and Neonatal Medicine2013.
6. Ersdal H, Vossius C, Bayo E, et al. A one-day “Helping Babies Breathe” course improves simulated performance but not clinical management of neonates. *Resuscitation.* 2013;84(10):1422-1427.
7. Mduma E, Ersdal H, Svensen E, Kidanto H, Auestad B, Perlman J. Frequent brief on-site simulation training and reduction in 24-h neonatal mortality—An educational intervention study. *Resuscitation.* 2015;93:1-7.
8. Bang A, Patel A, Bellad R, et al. Helping Babies Breathe (HBB) training: What happens to knowledge and skills over time? *BMC Pregnancy Childbirth.* 2016;16(1):364.
9. Bang A, Patel A, Bellad R, et al. Helping Babies Breathe (HBB) training: What happens to knowledge and skills over time? *BMC Pregnancy and Childbirth.* 2016;16(1):364.
10. Nagunwa TP, Lwoga ET. Developing an eLearning strategy to implement medical competency based curricula: Experiences from Muhimbili University of Health and Allied Sciences. Paper presented at: Education and e-Learning Innovations (ICEELI), 2012 International Conference on2012.
11. Frehywot S, Vovides Y, Talib Z, et al. E-learning in medical education in resource constrained low-and middle-income countries. *Human resources for health.* 2013;11(1):4.
12. Harun MH. Integrating e-learning into the workplace. *The Internet and higher education.* 2001;4(3):301-310.
13. Ruggeri K, Farrington C, Brayne C. A global model for effective use and evaluation of e-learning in health. *Telemedicine and e-Health.* 2013;19(4):312-321.
14. Joynes C. Distance learning for health: what works-A global review of accredited post-qualification training programmes for health workers in low and middle Income Countries. *London Int Dev Cent.* 2011;1.
15. Sweigart LI, Umoren RA, Scott PJ, et al. Virtual TeamSTEPPS® Simulations Produce Teamwork Attitude Changes Among Health Professions Students. *Journal of Nursing Education.* 2016;55(1):31-35.
16. Umoren RP, JA; Sweigart L; Rybas, N; Gossett, E; Johnson, M; Allen, M; Scott, PJ; Truman, B; Das, R. TEAMSTEPPS VIRTUAL TEAMS: Interactive virtual team training and practice for health professional learners *accepted for publication in Creative Nursing.*
17. Ericsson KA. Deliberate practice and the acquisition and maintenance of expert performance in medicine and related domains. *Academic medicine.* 2004;79(10):S70-S81.

18. Kapp KM. *The gamification of learning and instruction: game-based methods and strategies for training and education.* John Wiley & Sons; 2012.
19. Charmaz K. *Constructing grounded theory: A practical guide through qualitative analysis.* Sage; 2006.
20. Glaser BG. The constant comparative method of qualitative analysis. *Social problems.* 1965;12(4):436-445.

APPENDICES

Appendix 1. Abbreviations/Definition of Terms

AAP = American Academy of Pediatrics
BMGF = Bill and Melinda Gates Foundation
BMV = bag-and-mask ventilation
DHIS2 = District Health Information Software
eHBB = mobile/virtual reality Helping Babies Breathe
ECEB = Essential Care for Every Baby
ECSB = Essential Care for Small Babies
ENAP = Essential newborn action plan
ETAT+ = Emergency triage, assessment, and Treatment plus admission
HBB = Helping Babies Breathe
HBS = Helping Babies Survive
HCPs = Health care providers
LDHF = Low dose, high-frequency
LIFE = Life-saving Instruction for Emergencies
LMICs = Low/Middle income countries
mHBS = mobile Helping Babies Survive
MOH = Ministry of Health
MNCH = maternal newborn child health
NMR = neonatal mortality rate
NR = neonatal resuscitation
NRP = Neonatal Resuscitation Program
OSCE = Objective Structured Clinical Examination
PPV = positive-pressure ventilation
TOTs = Training of Trainers
VR = virtual reality
WHO = World Health Organization

Definition of Terms

3D simulation: These screen-based training scenarios are similar to the NRP eSIM® scenarios or the LIFE mobile game that enables learners practice the algorithm (e.g., “The Action Plan”) and reinforces knowledge of NR pathways but do not require a VR headset.

VR simulation: These training scenarios enable learners to practice both the NR algorithm and skills required for NR such as with the LIFE immersive VR NR game using a VR headset with or without hand controllers, e.g. Google cardboard, Google Daydream, Oculus Rift or HTC Vive.

Educational video: This is a video showing recommended NR algorithms, skills, and/or performance competencies related to preparation for delivery, immediate care at birth and/or successful newborn resuscitation using recommended HBB practices, such as videos offered by Global Health Media.

eHBB+mHBS/DHIS2 v.1

APPENDIX 2. STUDY DIAGRAM

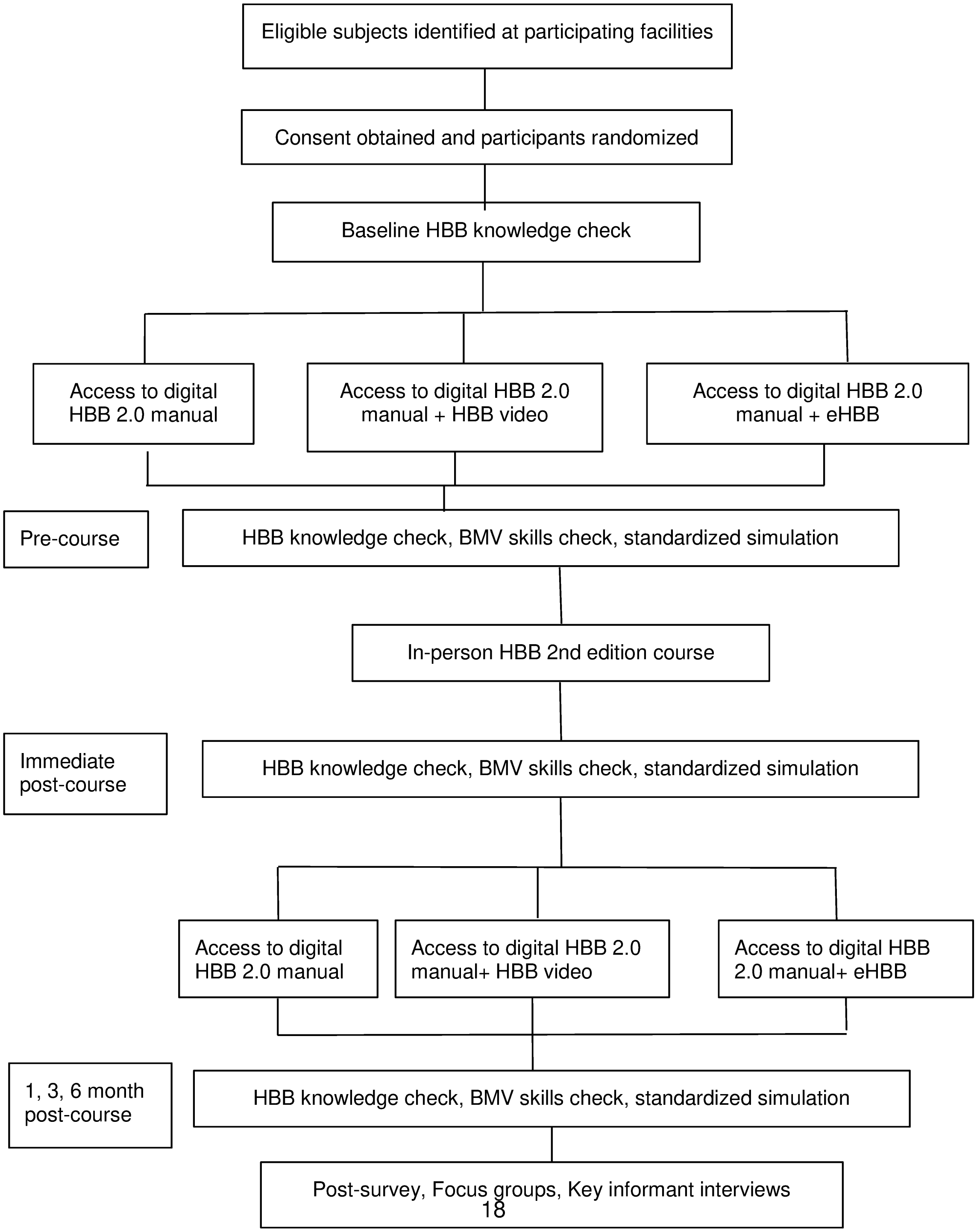

Appendix 3. Recruitment Information

You are invited to participate in the testing of a new mobile app for training on newborn resuscitation using the Helping Babies Breathe program.

We are recruiting health care workers and administrators with and without experience in using the Helping Babies Breathe program to assist with providing feedback on the application, particularly on whether it is easy to use and understand and whether it could be used in the future for training.

If you agree to participate, you will be assigned to one of three groups, eHBB, video or control group. You can use your own phone or a study phone to view the application using a simple virtual reality headset such as Google cardboard.

You will also receive training in the Helping Babies Breathe, 2nd edition program and your knowledge and skills in neonatal resuscitation will be observed by trained study personnel using a new type of manikin called the Neonatalie Advanced manikin. We will also ask you to answer questions about your experience either individually or as a group to help us understand your perspective on using these applications.

You will also receive a data credit to your phone as a thank you for your participation each month that you are in the study, for up to a six month period.

APPENDIX 4. CONSENT FORM

Approved
8/7/2018
UW HSD IRB

INFORMATION SHEET FOR STAKEHOLDER INTERVIEWS

eHBB: virtual-reality training game for resuscitation of newborns

Researchers: <Site PI, Department, Institution, Contact information>

Study PI: Dr. Rachel A. Umoren, Pediatrics, University of Washington, 202-543-3200

Researchers' statement

We are asking you to be in a research study. The purpose of this consent form is to give you the information you will need to help you decide whether to be in the study or not. Please read the form carefully. You may ask questions about the purpose of the research, what we would ask you to do, the possible risks and benefits, your rights as a volunteer, and anything else about the research or this form that is not clear. When we have answered all your questions, you can decide if you want to be in the study or not. This process is called "informed consent." We will give you a copy of this form for your records.

PURPOSE OF THE STUDY

Virtual reality has been used extensively in industry for training in various domains. We are conducting a research project to evaluate "eHBB", a mobile phone based virtual reality (VR) simulation of the *Helping Babies Breathe* program integrated with mHBS/DHIS2 for training healthcare workers on newborn resuscitation. The goal of this study is to explore the perceptions of stakeholders on VR simulation for healthcare training.

STUDY PROCEDURES

We would like you to participate in a 30-minute interview or one-hour focus group discussion during which you will have an opportunity to use a new VR application called eHBB and answer questions on your perceptions of VR-based training with DHIS2 data collection. If you agree, the interview will be digitally recorded for analysis by researchers. There will not be any personal questions. Participation in the study is completely voluntary. You may refuse to answer any question or item. You are free to not participate if you so choose. You can stop at any time without penalty.

RISKS, STRESS, OR DISCOMFORT

Some individuals experience motion sickness with using VR. Using VR is not recommended for individuals with seizure disorders. If you experience discomfort, please stop using the eHBB VR application and inform the study coordinator immediately. All study information will be kept confidential. There is a small risk of loss of confidentiality. However every effort will be made to minimize this risk by using password protection and secure storage of study records. Data collected during the study will be kept indefinitely and deidentified data may be shared with other researchers.

BENEFITS OF THE STUDY

There are no anticipated benefits to participating in this study.

SOURCE OF FUNDING

The development of the eHBB and mHBS apps is being supported by the Bill and Melinda Gates Foundation.

CONFIDENTIALITY OF RESEARCH INFORMATION

All of the information you provide will be confidential. Identifier data and observation data will be collected but they will not be linked. Government or university staff may sometimes review studies such as this one to make sure they are being done safely and legally. If a review of this study takes place, your records may be examined. The reviewers will protect your privacy. There are some limits to this protection.

OTHER INFORMATION

You may refuse to participate and you are free to withdraw from this study at any time without penalty or loss of benefits to which you are otherwise entitled. There are no costs to you for participating in the study. You will be provided with travel costs (if applicable), and internet data for downloading and using the application. There is no compensation for participating in the study. If you have questions, complaints or concerns about this study, you can contact Rachel Umoren at rumoren@uw.edu.

Approved
8/7/2018
UW HSD IRB

CONSENT FORM

eHBB: virtual-reality training game for resuscitation of newborns

Researchers: <Site PI, Department, Institution, Contact information>

Study PI: Dr. Rachel A. Umoren, Pediatrics, University of Washington, 202-543-3200

Researchers' statement

We are asking you to be in a research study. The purpose of this consent form is to give you the information you will need to help you decide whether to be in the study or not. Please read the form carefully. You may ask questions about the purpose of the research, what we would ask you to do, the possible risks and benefits, your rights as a volunteer, and anything else about the research or this form that is not clear. When we have answered all your questions, you can decide if you want to be in the study or not. This process is called "informed consent." We will give you a copy of this form for your records.

PURPOSE OF THE STUDY

Virtual reality has been used extensively in industry for training in various domains. We are conducting a research project to evaluate "eHBB", a mobile phone based virtual reality (VR) simulation of the *Helping Babies Breathe* program for training healthcare workers on newborn resuscitation. The goal of this study is to assess the impact of mobile VR simulation used before and after initial neonatal resuscitation training on educational outcomes, in comparison to watching a neonatal resuscitation video or traditional HBB training.

STUDY PROCEDURES

If you agree to participate, you will be randomly assigned to one of three study groups: eHBB (VR) group, neonatal resuscitation Video group, and standard HBB training material (Control) group. Participants will attend a full-day HBB 2nd edition course free of charge and receive a course completion certificate. Study participants will be given access to the study intervention for their group before the HBB class and for six months after the class. Participants' knowledge and skills in neonatal resuscitation will be assessed through standardized tests and simulations by trained study observers before, immediately after, and at 1, 3, and 6 months following the HBB 2nd edition course. Study researchers may also visit participating facilities to assess readiness for neonatal resuscitation and delivery practices. The estimated time for each simulation session is 20 minutes. The eHBB simulation and video takes less than 10 minutes per viewing session.

Regardless of group assignment, participants will have the opportunity to view the eHBB and mHBS/DHIS2 applications and provide feedback on their perceptions of VR-based training during one-hour focus groups and interviews at the end of the study. Data captured during simulations, interviews and focus group discussions will be digitally recorded for analysis by researchers. There will not be any personal or sensitive questions.

Participation in the study is completely voluntary. You may refuse to answer any question or item. You are free to not participate if you so choose. You can stop at any time without penalty.

RISKS, STRESS, OR DISCOMFORT

Some individuals experience motion sickness with using virtual reality. Using virtual reality is not recommended for individuals with seizure disorders. If you experience discomfort, please stop using the eHBB VR application and inform the study coordinator immediately. All study information will be kept confidential. There is a small risk of loss of confidentiality. However every effort will be made to minimize this risk by using password protection and secure storage of study records. Data collected during the study will be kept indefinitely and deidentified datasets may be shared with other researchers.

Approved
8/7/2018
UW HSD IRB

BENEFITS OF THE STUDY

Healthcare workers participating in the study may gain added expertise in newborn resuscitation.

SOURCE OF FUNDING

The development of the eHBB and mHBS apps is being supported by the Bill and Melinda Gates Foundation.

CONFIDENTIALITY OF RESEARCH INFORMATION

All of the information you provide will be confidential. Identifier data and observation data will be collected but they will not be linked. Government or university staff sometimes review studies such as this one to make sure they are being done safely and legally. If a review of this study takes place, your records may be examined. The reviewers will protect your privacy. The study records will not be used to put you at legal risk of harm. There are some limits to this protection.

OTHER INFORMATION

You may refuse to participate and you are free to withdraw from this study at any time without penalty or loss of benefits to which you are otherwise entitled.

There are no costs to you for participating in the study. You will be provided with travel costs (if applicable), course attendance fees, and internet data for downloading and using the application. There is no compensation for participating in the study.

RESEARCH-RELATED INJURY

If you have questions, complaints or concerns about this study, you can contact Rachel Umoren at rumoren@uw.edu. The UW does not normally provide compensation for harm except through its discretionary program for medical injury. You do not waive any right to seek payment by signing this consent form.

Printed name of researcher   Signature of researcher   Date

Subject's statement

This study has been explained to me. I volunteer to take part in this research. I have had a chance to ask questions. If I have questions later about the research, or if I have been harmed by participating in this study, I can contact one of the researchers listed on the first page of this consent form. If I have questions about my rights as a research subject, I can call the UW Human Subjects Division at (206) 543-0098 or call collect at (206) 221-5940. I will receive a copy of this consent form.

Printed name of participant   Signature of participant   Date

Copies to: Researcher
Participant

Appendix 5. Participant Surveys

Confidential

Demographic Survey

Study ID

____

Please complete the following survey

1. Age range

◯ less than 21 years ◯ 21 - 30 years ◯ 31 - 40 years ◯ 41 - 50 years ◯ More than 50 years

2. Sex

◯ Male ◯ Female

3. Profession

◯ Consultant physician
◯ Senior registrar
◯ Registrar
◯ Nurse
◯ Midwife
◯ Clinical Officer
◯ Other

profession_other

____

Please list your Degree/Certification in your area of practice

____

4. Years of practice since completion of training

◯ Less than 5 years ◯ 5 - 10 years ◯ 11 - 15 years ◯ 16 - 20 years ◯ More than 20 years

5. Current location of practice

◯ Nigeria ◯ Kenya ◯ Other

Other (please specify)

____

6. Health care level of practice

◯ Government (Tertiary care)
◯ Government (Secondary care)
◯ Government (Primary care)
◯ Private
◯ Faith-based facility

Healthcare Facility

____

Confidential

7. Specialty (if applicable)

◯ General Paediatrics
◯ Subspecialty Paediatrics
◯ Obstetrics & Gynaecology
◯ Subspecialty O & G
◯ Other specialty

Other Specialty (please specify)

____

8. Does your institution/health facility have facilities for simulation based training?

◯ Yes ◯ No

9. In what capacity does your institution use simulation-based training?

☐ Teaching ☐ Research ☐ Examination

10. Does your center have a skills-based simulation lab?

◯ Yes ◯ No

11. What is the skills-based simulation lab available for

☐ Skills practice, eg. HBB corner ☐ Teaching ☐ Research ☐ Examination

13. Which modality of simulation based training have you been exposed to?

☐ Manikin-based training (HBB)
☐ Manikin-based training (NRT)
☐ Manikin-based training (PALS)
☐ Manikin-based training (ENCC)
☐ Manikin-based training (BLS)
☐ NRP eSIM™
☐ HeartCode™ (PALS online course)
☐ Online Basic Life Support course
☐ Online ACLS course
☐ Virtual Reality Simulation (VR)
☐ Other (please specify below)

Other (please specify)

____

14. Are you aware of virtual reality simulation training?

◯ Yes ◯ No

15. When or where were you exposed to virtual reality simulation?

____

Confidential

16. What are the challenges to online (computer-based or virtual reality) simulation?

☐ Lack of awareness about VR based simulation
☐ Lack of internet access
☐ Lack of standardized VR training modules
☐ Inconsistent power supply
☐ Lack of access to VR equipment and computers
☐ Other

Other (please specify)

____

18. Are you aware that VR simulation can be run on a mobile phone so that you can learn skills on your own time and pace.

◯ Yes ◯ No

19. What type of mobile phone device do you own or use? (Choose all that apply)

◯ a. Tablet (e.g. Ipad, tablets)
◯ b.Smart Phone (e.g. Iphone, Samsung, Techno, Nexus, Infinix etc.)
◯ c. Feature phone (e.g. Does some gprs based activities)
◯ d.Basic (e.g. Used for call and SMS only)

20. What is the manufacturer and model of your phone/mobile device?

____

21. If you are using an android enabled device, what android version does your device run (To find out, Goto |Settings->General->AboutDevice| and look for version number)

◯ a. Gingerbread (version 2.3)
◯ b.Ice Cream Sandwich (version 4.0)
◯ c. Jelly Bean (version 4.1 - 4.3)
◯ d.KitKat (Version 4.4)
◯ e. Lollipop (Version 5.0 - 5.1)
◯ f. Marshmallow (Version 6.0)
◯ g. Nougat (Version 7)
◯ h.Oreo (Version 8)
◯ i. Other

If Other, please specify

____

22. Do you use mobile device (phone or tablet) currently for your work?

◯ Yes ◯ No

Please describe how you use your phone for work

Umoren R, *et al. BMJ Open* 2022; 11:e048506. doi: 10.1136/bmjopen-2020-048506

Confidential

25. If all facilities were available, would you recommend online simulation for training healthcare workers in your country?

◯ a. Yes ◯ b. No

If all facilities were available, would you recommend online simulation for your center?

◯ a. Yes ◯ b. No

26. Please state your reason(s)

Confidential

Follow Up Survey

Study ID ______________________

Please tell us a little about your participation in deliveries and ongoing HBB training in the last 6 months.

Approximately how many deliveries have you participated in during the last 6 months?
- ◯ none
- ◯ 1-5
- ◯ 6-10
- ◯ 11-15
- ◯ 16-20
- ◯ 21-25
- ◯ >25

How many deliveries have you participated in during the last 6 months that required resuscitation (at least one: bag-mask ventilation, chest compressions, intubation)? ______________________

Have you used the eHBB program in the last 6 months?
- ◯ No
- ◯ Yes

Have you watched the HBB neonatal resuscitation video in the last 6 months?
- ◯ No
- ◯ Yes

Please let us know about the quality of your training:

	Strongly disagree	Disagree	Neutral	Agree	Strongly agree
eHBB was easy to access	◯	◯	◯	◯	◯
eHBB was valuable for clinical practice	◯	◯	◯	◯	◯
eHBB was easy to navigate	◯	◯	◯	◯	◯
eHBB had realistic graphics	◯	◯	◯	◯	◯
eHBB provided valuable feedback	◯	◯	◯	◯	◯
the HBB video was easy to access	◯	◯	◯	◯	◯
the HBB video was valuable for clinical practice	◯	◯	◯	◯	◯
the HBB video was realistic	◯	◯	◯	◯	◯

Did you have any technical difficulties with the refresher training (eHBB or HBB videos) between your in person simulations?
- ◯ Yes
- ◯ No

If yes, please explain: ______________________

Would you use this method of refresher training again?
- ◯ Yes
- ◯ No

Confidential

If no, please explain:	__________________________
If you weren't able to complete your assigned refresher training to watch the HBB video or do the eHBB at the 1, 3 or 6 month mark from your HBB course, please tell us which barrier(s) contributed?	☐ I didn't receive a reminder<br>☐ The mHBS application didn't work<br>☐ I had trouble accessing the refreshers on mHBS<br>☐ I didn't have time to complete the refreshers<br>☐ I didn't find the refreshers relevant<br>☐ I didn't feel that I needed a refresher<br>☐ I didn't remember about the refreshers<br>☐ Other
If you chose other, please tell us about the barriers you experienced with the refreshers	__________________________
If you weren't able to complete your simulation practice at the 1, 3 or 6 month mark from your HBB course, please tell us which barrier(s) contributed?	☐ I didn't receive a reminder from the study personnel<br>☐ The practice location was not convenient<br>☐ I had trouble contacting the study personnel<br>☐ I didn't have time to complete the practice<br>☐ I didn't find the practice relevant<br>☐ I didn't feel that I needed the practice<br>☐ I didn't remember about the practice<br>☐ Other
If you chose other, please tell us about the barriers you experienced with attending the simulation practice	__________________________
Other comments or concerns:	__________________________

Umoren R, *et al. BMJ Open* 2022; 11:e048506. doi: 10.1136/bmjopen-2020-048506

APPENDIX 6. HBB KNOWLEDGE CHECK

Confidential

HBB Knowledge Check

Study ID	______________________________
1. What should you do in The Golden Minute?	◯ a. Bathe the baby<br>◯ b. Deliver the placenta<br>◯ c. Evaluate the heart rate<br>◯ d. Help a baby breathe if necessary
2. To prepare for a birth	◯ a. You identify a helper and review the emergency plan<br>◯ b. You ask everyone but the mother to leave the area<br>◯ c. You prepare equipment only when you need it<br>◯ d. You do not need a helper
3 To prepare the area for delivery	◯ a. Open all the doors and windows to get fresh air<br>◯ b. Darken the room<br>◯ c. Make sure the area is clean, warm, and well-lighted<br>◯ d. Keep the room temperature cold
4. What should you do to keep the baby warm?	◯ a. Open all the windows<br>◯ b. Give the baby a bath after birth<br>◯ c. Place hot water bottles next to the baby's skin<br>◯ d. Place the baby skin-to-skin with mother
5. What should you do to keep the baby clean?	◯ a. Wash your hands before touching the baby and help<br>◯ mother wash her hands before breastfeeding<br>◯ b. Reuse the suction device before cleaning<br>◯ c. Keep the umbilical cord tightly covered<br>◯ d. Do not touch the baby
6. Which baby can receive routine care after birth?	◯ a. A baby who is not breathing<br>◯ b. A baby who is gasping<br>◯ c. A baby who is crying and/or breathing well<br>◯ d. A baby who is limp
7. Routine care for a healthy baby at birth includes	◯ a. Drying, removing the wet cloth, and bathing the baby<br>◯ b. Drying, removing the wet cloth, and positioning<br>◯ the baby skin-to-skin<br>◯ c. Bathing and putting clean clothes on the baby<br>◯ d. Drying and wrapping the baby in the wet cloth
8. When should the umbilical cord be clamped or tied and cut during routine care?	◯ a. After the placenta is delivered<br>◯ b. Around 1-3 minutes after birth<br>◯ c. Immediately after the baby is born<br>◯ d. Before a baby has cried
9. A baby is quiet, limp and not breathing at birth. What should you do?	◯ a. Dry the baby thoroughly<br>◯ b. Shake the baby<br>◯ c. Throw cold water on the face<br>◯ d. Hold the baby upside down

Confidential

10. A newborn baby is quiet, limp and not crying. The baby does not respond to steps to stimulate breathing. What should you do next?	◯ a. Slap the baby's back<br>◯ b. Hold the baby upside down<br>◯ c. Squeeze the baby's ribs<br>◯ d. Begin ventilation
11. In which situation should a baby be suctioned?	◯ a. When a baby is crying at birth<br>◯ b. When a baby is crying but there is meconium in<br>◯ the amniotic fluid<br>◯ c. When you see secretions blocking the mouth and nose<br>◯ d. Before drying the baby
12. Suctioning a baby unnecessarily or frequently can	◯ a. Cause a baby to stop breathing<br>◯ b. Make a baby start coughing and breathing<br>◯ c. Stimulate a baby to cry<br>◯ d. Increase the baby's heart rate
13. Which of the following statements about ventilation with bag and mask is TRUE?	◯ a. The mask should cover the eyes<br>◯ b. Air should escape between the mask and face<br>◯ c. Squeeze the bag to produce gentle movement of the chest<br>◯ d. Squeeze the bag to give 80 to 100 breaths per minute
14. A baby's chest is not moving with bag and mask ventilation. What should you do?	◯ a. Stop ventilation<br>◯ b. Reapply the mask to get a better seal<br>◯ c. Slap the baby's back<br>◯ d. Give medicine to the baby
15. You can stop ventilation if	◯ a. A baby is blue and limp<br>◯ b. A baby's heart rate is slow<br>◯ c. A baby's heart rate is normal and the chest is not moving<br>◯ d. A baby's heart rate is normal and the baby is breathing or crying
16. A newborn baby's heart rate should be:	◯ a. Faster than your heart rate<br>◯ b. Slower than your heart rate<br>◯ c. Checked before drying the baby<br>◯ d. Checked only when the baby is crying
17. A baby who received ventilation	◯ a. Needs continued observation with mother<br>◯ b. Cannot be fed<br>◯ c. Always needs advanced care<br>◯ d. Should immediately receive antibiotics
18. When should the bag and mask and suction device be disinfected?	◯ a. After every use<br>◯ b. Only when they appear dirty<br>◯ c. Weekly<br>◯ d. Once a month

APPENDIX 7. SIMULATION CHECKLISTS

Confidential

BMV Skill Check

Study ID ______________________________

Complete this evaluation with participants before they attempt the OSCE evaluations.
- Read aloud the following instructions
- Use the comments below the numbered steps to score the performance
- Note the number of steps done correctly on the first attempt
- Read the feedback from Neonatalie Advanced ipad. Do not give any other feedback.

"You are attending the delivery of a term infant. You have prepared for the birth and tested the bag, mask, and suction device. You have dried and stimulated the baby, but the baby is not breathing. Show me how you will provide ventilation."

1. Begin to ventilate with bag and mask

	Done	Not done
Place the baby on the area for ventilation	☐	☐
Stand at the baby's head	☐	☐
Check that mask size is correct	☐	☐

2. Ventilate with bag and mask

	Done	Not done
Position the head slightly extended	☐	☐
Apply the mask to the face	☐	☐
Make a tight seal between the mask and the face	☐	☐
Squeeze the bag to produce gentle movement of the chest	☐	☐

3. Continue ventilation (for 1 minute)

	Done	Not done
Ventilate to produce gentle movement of the chest with each ventilation breath	☐	☐
Ventilate at 40 breaths/minute (30-50 breaths/minute acceptable)	☐	☐

The baby's chest has stopped moving with ventilation. Show me what you would do to improve ventilation.

Umoren R, *et al. BMJ Open* 2022; 11:e048506. doi: 10.1136/bmjopen-2020-048506

Confidential

4. Improve ventilation

	Done	Not done
Reapply mask	☐	☐
Reposition head	☐	☐
Clear mouth and nose of secretions	☐	☐
Open the mouth	☐	☐
Squeeze the bag harder	☐	☐

Score on first attempt ________________________________

Umoren R, *et al. BMJ Open* 2022; 11:e048506. doi: 10.1136/bmjopen-2020-048506

Confidential

OSCE A

Study ID ______________________________

Objective Structured Clinical Evaluations (OSCEs) can be used to determine whether participants have learned the essential steps to help a baby breathe. They can be used to verify that a participant knows enough to pass the course, or also as an exercise repeated regularly for practice. Most importantly, each completed evaluation should be used as an opportunity for the participant to review and learn.

Read the case scenario aloud to the participant. Provide the prompts shown in red. Indicate the baby's response to the participant's actions using the neonatal simulator or words if using a mannequin. For example, when the participants evaluate crying, show that the baby is not crying with a simulator. Say that the baby is not crying if using a mannequin. As you observe the participant, tick the boxes "Done" or "Not Done" for each activity. Apart from giving these prompts, keep silent during the evaluation.

After participants complete the OSCE, ask the 5 questions written below OSCE A. These questions will help the participants reflect on what actions they took and what they can do better the next time. Participants who can recognize their own mistakes will better remember the right steps to take the next time. Comment on the participant's performance only at the end of the case, after he/she has answered these 5 questions.

"I am going to read a role play case. Please listen carefully, and then show me the actions you would take. I will indicate the
baby's responses, but I will provide no other feedback until the end of the case."

"You are called to assist the delivery of a term baby. There are no complications in the pregnancy. The baby will be born in less than 10 minutes. Introduce yourself and prepare for the birth and care of the baby."

	Done	Not Done
Identifies a helper and reviews an emergency plan.	○	○
Prepares the area for delivery (warm, well-lighted, clean)	○	○
Washes hands	○	○
Prepares an area for ventilation and checks function of bag, mask and suction device	○	○

Prompt: After 5-7 minutes give baby to participant and say, "There is meconium in the amniotic fluid.
The baby is delivered onto the mother's abdomen. Show how you will care for the baby."

	Done	Not Done
Dries thoroughly	○	○
Removes wet cloth	○	○

Confidential

Prompt: Show the baby is not crying. "There is meconium blocking the mouth."

	Done	Not Done
Recognizes baby is not crying	○	○
Positions head and clears airway	○	○
Stimulates breathing by rubbing the back	○	○

Prompt: Show the baby is breathing well (cries)

	Done	Not Done
Recognizes baby is crying and breathing well	○	○
Clamps or ties and cuts the cord	○	○
Positions skin-to-skin on mother's chest and puts on the head covering	○	○
Communicates with mother	○	○

Use the questions below to help the participant reflect on his or her own performance and then provide feedback.
1. What happened at the birth?
2. Did you follow the Action Plan?
3. What went well and what could have gone better?
4. What did you learn?
5. What will you do differently next time?

Comments

____

Umoren R, *et al. BMJ Open* 2022; 11:e048506. doi: 10.1136/bmjopen-2020-048506

Confidential

OSCE B

Study ID ______________________________

Instructions to the facilitator: Read the below instructions for the case scenario.

"I am going to read a role play case. Please listen carefully, and then show me the actions you would take. I will indicate the baby's responses, but I will provide no other feedback until the end of the case."

"You are called to assist at the birth of 34 week (7-1/2 months) gestation baby. You have identified a helper, prepared an area for ventilation, washed your hands, and checked your equipment. The baby is born, and the amniotic fluid is clear.
Show how you will care for the baby."

	Done	Not Done
Dries thoroughly	○	○
Removes wet cloth	○	○

Prompt: Show the baby is not crying. "You do not see or hear secretions in the baby's mouth or nose."

	Done	Not Done
Recognizes baby is not crying	○	○
Stimulates breathing by rubbing the back	○	○

Prompt: Show the baby is not breathing

	Done	Not Done
Recognizes baby is not breathing	○	○
Cuts cord and moves to area for ventilation OR positions by mother for ventilation	○	○
Ventilates with bag and mask within The Golden Minute (at ____seconds)	○	○
Achieves a firm seal as demonstrated by chest movement	○	○
Time of effective ventilation (chest moving gently at _____ seconds)	○	○
Ventilates at 40 breaths/minute (30-50 acceptable)	○	○
Evaluates for breathing or chest movement	○	○

Umoren R, *et al. BMJ Open* 2022; 11:e048506. doi: 10.1136/bmjopen-2020-048506

Confidential

Prompt: Show the baby is not breathing.

	Done	Not Done
Recognizes baby is not breathing	○	○
Calls for help	○	○
Continues ventilation	○	○

Prompt: Say, "Please show what to do if the chest is not moving with ventilation." After one or more steps to improve ventilation, say "The chest is moving now."

	Done	Not Done
Reapplies mask	○	○
Repositions head	○	○
Clears secretions from the mouth and nose as needed	○	○
Opens mouth slightly	○	○
Squeeze bag harder	○	○

Prompt: Show the baby is not breathing; heart rate is normal.

	Done	Not Done
Recognizes baby is not breathing but heart rate is normal	○	○
Continues ventilation	○	○

Prompt: (After 3 minutes) Show the heart rate is 120 per minute and the baby is breathing.

	Done	Not Done
Recognizes baby is breathing and heart rate is normal	○	○
Stops ventilation	○	○
Provides close observation for the baby and communicates with the mother	○	○

Use the questions below to help the participant reflect on his or her own performance and then provide feedback.
1. What happened at the birth?
2. Did you follow the Action Plan?
3. What went well and what could have gone better?
4. What did you learn?
5. What will you do differently next time?

Comments

____

Umoren R, *et al. BMJ Open* 2022; 11:e048506. doi: 10.1136/bmjopen-2020-048506
