# Supplementary material for: eHBB: a randomised controlled trial of virtual reality or video for neonatal resuscitation refresher training in healthcare workers in resource-scarce settings
Source: BMJ Open. 2021 Aug 24;11(8):e048506. doi: 10.1136/bmjopen-2020-048506 (PMC8390148; doi:10.1136/bmjopen-2020-048506)
Supplement: Supplementary data [file bmjopen-2020-048506supp002.pdf]

SUPPLEMENTARY FILE 2

SAMPLE CONSENT FORM

Approved
8/7/2018
UW HSD IRB

INFORMATION SHEET FOR STAKEHOLDER INTERVIEWS

eHBB: virtual-reality training game for resuscitation of newborns

Researchers: <Site PI, Department, Institution, Contact information>
Study PI: Dr. Rachel A. Umoren, Pediatrics, University of Washington, 202-543-3200

Researchers' statement

We are asking you to be in a research study. The purpose of this consent form is to give you the information you will need to help you decide whether to be in the study or not. Please read the form carefully. You may ask questions about the purpose of the research, what we would ask you to do, the possible risks and benefits, your rights as a volunteer, and anything else about the research or this form that is not clear. When we have answered all your questions, you can decide if you want to be in the study or not. This process is called "informed consent." We will give you a copy of this form for your records.

PURPOSE OF THE STUDY

Virtual reality has been used extensively in industry for training in various domains. We are conducting a research project to evaluate "eHBB", a mobile phone based virtual reality (VR) simulation of the *Helping Babies Breathe* program integrated with mHBS/DHIS2 for training healthcare workers on newborn resuscitation. The goal of this study is to explore the perceptions of stakeholders on VR simulation for healthcare training.

STUDY PROCEDURES

We would like you to participate in a 30-minute interview or one-hour focus group discussion during which you will have an opportunity to use a new VR application called eHBB and answer questions on your perceptions of VR-based training with DHIS2 data collection. If you agree, the interview will be digitally recorded for analysis by researchers. There will not be any personal questions. Participation in the study is completely voluntary. You may refuse to answer any question or item. You are free to not participate if you so choose. You can stop at any time without penalty.

RISKS, STRESS, OR DISCOMFORT

Some individuals experience motion sickness with using VR. Using VR is not recommended for individuals with seizure disorders. If you experience discomfort, please stop using the eHBB VR application and inform the study coordinator immediately. All study information will be kept confidential. There is a small risk of loss of confidentiality. However every effort will be made to minimize this risk by using password protection and secure storage of study records. Data collected during the study will be kept indefinitely and deidentified data may be shared with other researchers.

BENEFITS OF THE STUDY

There are no anticipated benefits to participating in this study.

SOURCE OF FUNDING

The development of the eHBB and mHBS apps is being supported by the Bill and Melinda Gates Foundation.

CONFIDENTIALITY OF RESEARCH INFORMATION

All of the information you provide will be confidential. Identifier data and observation data will be collected but they will not be linked. Government or university staff may sometimes review studies such as this one to make sure they are being done safely and legally. If a review of this study takes place, your records may be examined. The reviewers will protect your privacy. There are some limits to this protection.

OTHER INFORMATION

You may refuse to participate and you are free to withdraw from this study at any time without penalty or loss of benefits to which you are otherwise entitled. There are no costs to you for participating in the study. You will be provided with travel costs (if applicable), and internet data for downloading and using the application. There is no compensation for participating in the study. If you have questions, complaints or concerns about this study, you can contact Rachel Umoren at rumoren@uw.edu.

Approved
8/7/2018
UW HSD IRB

CONSENT FORM

eHBB: virtual-reality training game for resuscitation of newborns

Researchers: <Site PI, Department, Institution, Contact information>

Study PI: Dr. Rachel A. Umoren, Pediatrics, University of Washington, 202-543-3200

Researchers' statement

We are asking you to be in a research study. The purpose of this consent form is to give you the information you will need to help you decide whether to be in the study or not. Please read the form carefully. You may ask questions about the purpose of the research, what we would ask you to do, the possible risks and benefits, your rights as a volunteer, and anything else about the research or this form that is not clear. When we have answered all your questions, you can decide if you want to be in the study or not. This process is called "informed consent." We will give you a copy of this form for your records.

PURPOSE OF THE STUDY

Virtual reality has been used extensively in industry for training in various domains. We are conducting a research project to evaluate "eHBB", a mobile phone based virtual reality (VR) simulation of the *Helping Babies Breathe* program for training healthcare workers on newborn resuscitation. The goal of this study is to assess the impact of mobile VR simulation used before and after initial neonatal resuscitation training on educational outcomes, in comparison to watching a neonatal resuscitation video or traditional HBB training.

STUDY PROCEDURES

If you agree to participate, you will be randomly assigned to one of three study groups: eHBB (VR) group, neonatal resuscitation Video group, and standard HBB training material (Control) group. Participants will attend a full-day HBB 2nd edition course free of charge and receive a course completion certificate. Study participants will be given access to the study intervention for their group before the HBB class and for six months after the class. Participants' knowledge and skills in neonatal resuscitation will be assessed through standardized tests and simulations by trained study observers before, immediately after, and at 1, 3, and 6 months following the HBB 2nd edition course. Study researchers may also visit participating facilities to assess readiness for neonatal resuscitation and delivery practices. The estimated time for each simulation session is 20 minutes. The eHBB simulation and video takes less than 10 minutes per viewing session.

Regardless of group assignment, participants will have the opportunity to view the eHBB and mHBS/DHIS2 applications and provide feedback on their perceptions of VR-based training during one-hour focus groups and interviews at the end of the study. Data captured during simulations, interviews and focus group discussions will be digitally recorded for analysis by researchers. There will not be any personal or sensitive questions.

Participation in the study is completely voluntary. You may refuse to answer any question or item. You are free to not participate if you so choose. You can stop at any time without penalty.

RISKS, STRESS, OR DISCOMFORT

Some individuals experience motion sickness with using virtual reality. Using virtual reality is not recommended for individuals with seizure disorders. If you experience discomfort, please stop using the eHBB VR application and inform the study coordinator immediately. All study information will be kept confidential. There is a small risk of loss of confidentiality. However every effort will be made to minimize this risk by using password protection and secure storage of study records. Data collected during the study will be kept indefinitely and deidentified datasets may be shared with other researchers.

Approved
8/7/2018
UW HSD IRB

BENEFITS OF THE STUDY

Healthcare workers participating in the study may gain added expertise in newborn resuscitation.

SOURCE OF FUNDING

The development of the eHBB and mHBS apps is being supported by the Bill and Melinda Gates Foundation.

CONFIDENTIALITY OF RESEARCH INFORMATION

All of the information you provide will be confidential. Identifier data and observation data will be collected but they will not be linked. Government or university staff sometimes review studies such as this one to make sure they are being done safely and legally. If a review of this study takes place, your records may be examined. The reviewers will protect your privacy. The study records will not be used to put you at legal risk of harm. There are some limits to this protection.

OTHER INFORMATION

You may refuse to participate and you are free to withdraw from this study at any time without penalty or loss of benefits to which you are otherwise entitled.

There are no costs to you for participating in the study. You will be provided with travel costs (if applicable), course attendance fees, and internet data for downloading and using the application. There is no compensation for participating in the study.

RESEARCH-RELATED INJURY

If you have questions, complaints or concerns about this study, you can contact Rachel Umoren at rumoren@uw.edu. The UW does not normally provide compensation for harm except through its discretionary program for medical injury. You do not waive any right to seek payment by signing this consent form.

Printed name of researcher   Signature of researcher   Date

Subject's statement

This study has been explained to me. I volunteer to take part in this research. I have had a chance to ask questions. If I have questions later about the research, or if I have been harmed by participating in this study, I can contact one of the researchers listed on the first page of this consent form. If I have questions about my rights as a research subject, I can call the UW Human Subjects Division at (206) 543-0098 or call collect at (206) 221-5940. I will receive a copy of this consent form.

Printed name of participant   Signature of participant   Date

Copies to: Researcher
Participant
