# Supplementary material for: eHBB: a randomised controlled trial of virtual reality or video for neonatal resuscitation refresher training in healthcare workers in resource-scarce settings
Source: BMJ Open. 2021 Aug 24;11(8):e048506. doi: 10.1136/bmjopen-2020-048506 (PMC8390148; doi:10.1136/bmjopen-2020-048506)
Supplement: Author's manuscript [file bmjopen-2020-048506.draft_revisions.pdf]

BMJ Open is committed to open peer review. As part of this commitment we make the peer review history of every article we publish publicly available.

When an article is published we post the peer reviewers' comments and the authors' responses online. We also post the versions of the paper that were used during peer review. These are the versions that the peer review comments apply to.

The versions of the paper that follow are the versions that were submitted during the peer review process. They are not the versions of record or the final published versions. They should not be cited or distributed as the published version of this manuscript.

BMJ Open is an open access journal and the full, final, typeset and author-corrected version of record of the manuscript is available on our site with no access controls, subscription charges or pay-per-view fees (http://bmjopen.bmj.com).

If you have any questions on BMJ Open's open peer review process please email info.bmjopen@bmj.com

BMJ Open

eHBB: A randomized controlled trial of virtual reality or video for neonatal resuscitation refresher training in healthcare workers

Journal:	*BMJ Open*
Manuscript ID	bmjopen-2020-048506
Article Type:	Original research
Date Submitted by the Author:	30-Dec-2020

Complete List of Authors:	Umoren, Rachel; University of Washington<br>Bucher, Sherri; Indiana University School of Medicine<br>Hippe, Daniel; University of Washington<br>Ezenwa, Beatrice; University of Lagos College of Medicine<br>Fajolu, Iretiola; University of Lagos College of Medicine<br>Okwako, Felicitas; Alupe University College<br>Feltner, John; University of Washington<br>Nafula, Mary; Alupe University College<br>Musale, Annet; Alupe University College<br>Olawuyi, Olubukola; University of Lagos College of Medicine<br>Adeboboye, Christianah; University of Lagos College of Medicine<br>Asangansi, Ime; eHealth4everyone<br>Paton, Chris; Nuffield Department of Clinical Medicine, Centre for Tropical Medicine<br>Purkayastha, Saptarshi; Indiana University Purdue University at Indianapolis<br>Ezeaka, Chinyere; University of Lagos College of Medicine<br>Esamai, Fabian; Alupe University College

Keywords:	PAEDIATRICS, Information technology < BIOTECHNOLOGY & BIOINFORMATICS, EDUCATION & TRAINING (see Medical Education & Training), Neonatal intensive & critical care < INTENSIVE & CRITICAL CARE, MEDICAL EDUCATION & TRAINING, NEONATOLOGY

SCHOLARONE™
Manuscripts

I, the Submitting Author has the right to grant and does grant on behalf of all authors of the Work (as defined in the below author licence), an exclusive licence and/or a non-exclusive licence for contributions from authors who are: i) UK Crown employees; ii) where BMJ has agreed a CC-BY licence shall apply, and/or iii) in accordance with the terms applicable for US Federal Government officers or employees acting as part of their official duties; on a worldwide, perpetual, irrevocable, royalty-free basis to BMJ Publishing Group Ltd ("BMJ") its licensees and where the relevant Journal is co-owned by BMJ to the co-owners of the Journal, to publish the Work in this journal and any other BMJ products and to exploit all rights, as set out in our licence.

The Submitting Author accepts and understands that any supply made under these terms is made by BMJ to the Submitting Author unless you are acting as an employee on behalf of your employer or a postgraduate student of an affiliated institution which is paying any applicable article publishing charge ("APC") for Open Access articles. Where the Submitting Author wishes to make the Work available on an Open Access basis (and intends to pay the relevant APC), the terms of reuse of such Open Access shall be governed by a Creative Commons licence – details of these licences and which Creative Commons licence will apply to this Work are set out in our licence referred to above.

Other than as permitted in any relevant BMJ Author's Self Archiving Policies, I confirm this Work has not been accepted for publication elsewhere, is not being considered for publication elsewhere and does not duplicate material already published. I confirm all authors consent to publication of this Work and authorise the granting of this licence.

For peer review only

eHBB: A randomized controlled trial of virtual reality or video for neonatal resuscitation refresher training in healthcare workers

Rachel A. Umoren*, MB BCh, MS,[a] Sherri Bucher*, PhD, MA,[b] Daniel S. Hippe MS, [a] Beatrice Ezenwa, MBBS, MSc, MD,[c] Iretiola B. Fajolu, MBBS, [c] Felicitas M. Okwako, MB.ChB, MMED,[d] John Feltner, MS,[a] Mary Nafula, BSN, [d] Annet Musale, [d] Olubukola A. Olawuyi, BNSc, MSc, [c] Christianah O. Adeboboye, BNSc, [c] Ime Asangansi MBBS, PhD,[e] Chris Paton, BMBS, BMedSci, MBA,[f] Saptarshi Purkayastha, PhD,[g] Chinyere Ezeaka§, MBBS, MPH, [c] Fabian Esamai§, MB ChB.[d]

**Contributed equally as co-first authors*
§Contributed equally as co-senior authors

Affiliations:
[a]University of Washington, Seattle, Washington, U.S.A.
[b]Indiana University School of Medicine, Indianapolis, Indiana, U.S.A.
[c]College of Medicine, University of Lagos, Lagos, Nigeria
[d]Alupe University College, Busia, Kenya
[e]eHealth4everyone, Abuja, Nigeria
[f]University of Oxford, Oxford, U.K.
[g]Indiana University – Purdue University, Indianapolis, Indiana, U.S.A

Address correspondence to: Rachel A. Umoren, Department of Pediatrics, Division of Neonatology, 1959 NE Pacific St, Box 356320, Seattle, Washington, 98195, U.S.A. Telephone: 206-543-3200. Email address: rumoren@uw.edu

Text word count: 3422

Keywords: neonatology, nursing, pediatrics, medical education, information technology

For peer review only - http://bmjopen.bmj.com/site/about/guidelines.xhtml

ABSTRACT

Objective: To assess the impact of mobile virtual reality (VR) simulations using electronic HBB (eHBB) or video for the maintenance of neonatal resuscitation skills in healthcare workers.

Design: Randomized controlled trial with 6-month follow-up (2018-2020).

Setting: Secondary and tertiary healthcare facilities.

Participants: 274 nurses and midwives assigned to labor and delivery, operating room, and newborn care units were recruited from 20 healthcare facilities in Nigeria and Kenya and randomized to one of three groups: VR (eHBB + digital guide), Video (video + digital guide), or Control (digital guide only) groups before an in-person HBB course.

Intervention(s): eHBB VR simulation or neonatal resuscitation video.

Main outcome(s): Healthcare worker neonatal resuscitation skills using standardized checklists in a simulated setting at 1 month, 3 months and 6 months.

Results: Neonatal resuscitation skills pass-rates were similar among the groups at 6-month follow-up for bag-and-mask ventilation (BMV) skills check (VR 28%, Video 25%, Control 22%, p=0.71), OSCE A (VR 76%, Video 76%, Control 72%, p=0.78) and OSCE B (VR 62%, Video 60, Control 49%, p=0.18). Relative to the immediate post-course assessments, there was greater retention of BMV skills at 6 months in the VR group (-15% VR, p=0.10; -21% video, p<0.01, -27% control, p=0.001). OSCE B pass-rates in the VR group were numerically higher at 3 months (+4%, p=0.64) and 6 months (+3%, p=0.74) and lower in the Video (-21% at 3 months, p<0.001; -14% at 6 months, p=0.066) and Control groups (-7% at 3 months, p=0.43; -14% at 6 months, p=0.10). On follow-up survey, 95%(n=65) of respondents in the VR group and 98% (n=82) in the Video group would use their assigned intervention again.

Conclusion: eHBB VR training was highly acceptable to healthcare workers in low-middle income countries and may provide additional support for neonatal resuscitation skills retention compared to other digital interventions.

ARTICLE SUMMARY

Strengths and limitations of this study

- This study was a multi-center, randomized controlled trial of mobile virtual reality or video to support neonatal resuscitation skills retention in nurses and midwives who provide neonatal resuscitation in two low-middle income countries.
- The study used an evidence-based Helping Babies Breathe 2nd edition curriculum designed for neonatal resuscitation training in resource-scarce settings.
- Healthcare workers accessed the digital interventions on mobile phones in the 6 months following in-person Helping Babies Breathe training.
- Healthcare workers were recruited from secondary and tertiary healthcare settings in urban and semi-urban areas, so the study findings may not apply to healthcare workers in primary healthcare settings or in rural areas.

For peer review only

For peer review only - http://bmjopen.bmj.com/site/about/guidelines.xhtml

INTRODUCTION

In 2019, there were 2.4 million deaths among infants under 28 days of age.[1 2] These neonatal deaths now account for 47% of global under 5-years child mortality,[2] and most cases are preventable.[3-5] The majority of these deaths occur in low-middle income countries (LMICs). Nigeria, with a neonatal mortality rate of 36 deaths per 1000 live births and Kenya at 21 deaths per 1000 live births in 2019,[1] are at significant risk of failing to meet the United Nations Sustainable Development Goal 3 (SDG 3) to reduce neonatal mortality to 12 per 1,000 live births by 2030.[8]

Intrapartum asphyxia or lack of breathing at birth is a leading cause of neonatal mortality.[6 7] Training healthcare workers in neonatal resuscitation using the *Helping Babies Breathe* (HBB) curriculum builds competency and reduces newborn morbidity and mortality.[8-13] However, neonatal resuscitation skills are quickly lost after trainings that utilize traditional methods.[14-18] For this reason, periodic refresher training with “low-dose high frequency” manikin-based simulations are recommended to support neonatal resuscitation skills retention.[19 20] Unfortunately, access to manikin-based simulation is limited by trainer, space, and equipment availability.[21 22] Yet, the high penetration of smartphones and cellular network connectivity in urban and rural areas in LMICs, makes innovative simulation training feasible using mobile virtual reality (VR) simulations for healthcare workers who provide care in health facility and community-based settings.

VR simulations are effective educational tools and can be engaged at the learner’s convenience, on their own smartphone, with game-based automated feedback that is ideal for episodic learning.[23-25] However little is known of their feasibility, acceptability, or effectiveness for NR skills retention in LMICs. We hypothesized that mobile VR simulation refresher training would address challenges to the quality of newborn resuscitation related to the maintenance of HCW

For peer review only

For peer review only - http://bmjopen.bmj.com/site/about/guidelines.xhtml

knowledge and skills over time addressing the lack of standardized dissemination of updates to recommended practice and high rates of staff turnover. The objective of this study was to evaluate the impact of eHBB VR used with in-person neonatal resuscitation training on neonatal resuscitation educational indicators and performance outcomes, in comparison to other digital refresher training modalities.

METHODS

Study setting: The study was conducted in Lagos, Nigeria and Busia, Western Kenya. Twelve healthcare facilities (9 secondary and 3 tertiary) were located in Nigeria while 8 facilities were located in Kenya. The healthcare facilities were located in urban and semi-urban areas and all have maternal and newborn services with newborn bed capacity ranging from 2-80 beds and delivery and neonatal unit staffing capacity from 7-124 nurses. The study was approved by the University of Washington Institutional Review Board (IRB), the Indiana University IRB, the Moi University, Health Research Ethics Committee and the University of Lagos College of Medicine Health Research Ethics Committee.

Participants: Study participants consisted of nurses and nurse-midwives assigned to labor and delivery, operating room, and newborn care units. Site coordinators or research assistants requested contact numbers, units, and wards of potential participants from head nurses at identified facilities. Research assistants contacted individuals to determine eligibility and obtained consent.

Inclusion Criteria: Nurses and midwives who participate in deliveries and who provide neonatal resuscitation to inborn or outborn infants and provide study consent.

Exclusion Criteria: Those who had attended a NR training course in the 1 year preceding the study; individuals who did not provide neonatal resuscitation as part of their duties or would be

For peer review only - http://bmjopen.bmj.com/site/about/guidelines.xhtml

unavailable or unwilling to participate in follow-up study activities throughout the 6-month post-initial training period.

Randomization: Study IDs generated for each country site were randomly assigned via a computer-generated algorithm to the VR, Video, and Control groups by a U.S. based study coordinator. Participants were enrolled and assigned a study ID before the HBB course.by local study coordinators. Each participant received an Android study phone, pre-loaded with permission-based access linked to their study ID, via the mobile Helping Babies Survive powered by DHIS2 app (mHBS/DHIS2), to the participant's assigned digital intervention. The data analysis team was blinded to the study assignments.

HBB course structure: The HBB provider course (Second Edition)[26] was taught by study HBB master trainers as one-day, 8-hour long sessions from December 2018-August 2019. A 30-minute orientation was provided on use of the mHBS/DHIS2 app, including how to access the assigned digital intervention. The features of eHBB VR have been previously described.[25 27]A total of 274 healthcare workers participated in the in-person Helping Babies Breathe training.

Pre- and post-course assessments: Standardized knowledge and skills assessments were conducted by trained research assistants. The HBB Knowledge Check (15 of 18 multiple-choice questions, ≥80% required to pass) and Bag-and Mask Ventilation Skill Check (BMV; 14 of 14 items required to pass) were conducted pre- and post-course along with the Objective Structured Clinical Examination (OSCE) A checklist on preparation for delivery and initial steps of resuscitation (9 out of 12 items and 3 required items to pass). In addition, the post-course

For peer review only - http://bmjopen.bmj.com/site/about/guidelines.xhtml

assessment included the OSCE B checklist on prolonged newborn resuscitation (17 out of 23 items and 6 required items to pass). A demographic survey was completed. See Figure 1.

Figure 1. Study Design

Post-course interventions and follow-up: Following the course, participants were encouraged to use their assigned digital intervention weekly and to engage in standard bag-and-mask skills practice with a manikin at the HBB practice corner set up at their facility. Post-course assessments were repeated at 1, 3, and 6 months after the class. A follow-up survey was completed.

Data collection: Data were collected in person by trained study staff using the mHBS/DHIS2 Tracker app for offline data collection[25]. The Tracker app contained digitized HBB knowledge check, BMV skill check, and OSCE A and OSCE B checklist. The mHBS Trainer app and eHBB app separately tracked access and use. To standardize data collection and feedback to study participants, an enhanced neonatal simulator, called *NeoNatalie Live* [Laerdal Medical] was used for bag-and-mask ventilation. Compared to the low-fidelity *NeoNatalie* simulators used for HBB training (including the HBB practice corners in this study), *NeoNatalie Live* manikin can be programmed to simulate key physiological parameters, such as variable rates of lung stiffness and heart and provides auditory and visual cues, in the form of “crying” and increased heart rate when the end-user provides bag-and-mask ventilation[28]. In addition, brief automated feedback for the end-user is provided using a Bluetooth-connected tablet device at the end of the assessment as “well done” or “needs improvement” based on bag and mask performance. The automated

For peer review only - http://bmjopen.bmj.com/site/about/guidelines.xhtml

feedback provided by *Neonatalie Live* was the only feedback provided following each assessment.[28]

Patients or public involvement: Patients or the public were not involved in the design, or conduct, or reporting, or dissemination plans of our research.

Sample size calculations: We hypothesized that there will be at least a 20% difference in the proportion of subjects who pass OSCE B at the 6-month evaluation between the VR group or Video group and the Control group. A sample size of 83 subjects per group would provide 80% power to detect a difference in pass rates between groups if the true pass rates were 85% and 65%, respectively, based on a two-sided $\alpha = 0.05$. The required total sample size for the three groups (VR, Video, and Control) was 249. We recruited 274 participants total to allow for 10% dropout over the 6-month follow-up period.

Data Analysis: An intention-to-treat analysis was performed, where participants were grouped according to their randomly allocated experimental group (VR, Video, or Control) regardless of their actual exposure. Fisher's exact test was used to test for any differences in pass rates among the three groups for each of the study evaluations: Bag-and-mask ventilation skills assessment (BMV), and standardized simulations of routine care and initial resuscitation (OSCE A) and prolonged resuscitation (OSCE B). Post-hoc pairwise comparisons and comparisons between demographic groups were also performed using Fisher's exact test. Within-group comparisons of evaluation results between timepoints were performed using the Sign test. Participant exposure to the interventions (time in the mHBS Trainer app) and self-reported clinical activity during the follow-up period were compared between experimental groups using the Kruskal-Wallis test and

For peer review only

For peer review only - http://bmjopen.bmj.com/site/about/guidelines.xhtml

Wilcoxon rank-sum test. All statistical calculations were conducted with the statistical computing language R (version 4.0.0; R Foundation for Statistical Computing, Vienna, Austria). Throughout, two-sided tests were used, with statistical significance defined as $p<0.05$.

RESULTS

Recruitment, training and follow-up were conducted concurrently at participating sites from December 2018 to August 2019 with follow-up continuing until February 2020. Of the 394 nurses and nurse-midwives identified who attended deliveries at the participating sites, 274 consented to participate in the study. Of the 274 participants, 265 (97% completed a 6-month assessment, with a similar dropout rate in each group (p=0.52 for the difference between groups, Figure 2).

Figure 2. CONSORT Diagram

Most participants were female (91%), nurse-midwives (51%), who worked in the labor and delivery ward (72%). Nearly all owned a smartphone (Table 1). Neonatal resuscitation knowledge and skills assessments were conducted immediately after the in-person course. There were no differences in Knowledge check scores [VR 18(17-18), Video 18(17-18), Control 18(17-18), p 0.76] or pass-rates on the BMV [VR 46%(n=83), Video 46%(n=84), Control 52%(n=79), p=0.72], OSCE A [VR 76%(n=91), 78%(n=95), 72%(n=88), p=0.63] and OSCE B [VR 59%(n=91), Video 73%(n=95), Control 62%(n=88), 0.13] assessments across groups on the immediate post-course assessments.

For peer review only

For peer review only - http://bmjopen.bmj.com/site/about/guidelines.xhtml

Table 1 Demographics of study participants

Demographics	All<br>n = 274<br>n(%)	VR + Digital Guide<br>n=91<br>n(%)	Video + Digital Guide<br>n=95<br>n (%)	Digital HBB Guide only<br>n=88<br>n (%)
**Age,** years, mean(SD)	38 (9)	37(9)	41(10)	37 (9)
**Gender**				
Female	250 (91.2)	82 (90.1)	88 (92.6)	80 (90.9)
**Profession**				
Nurse	133 (48.5)	43 (47.3)	46 (48.4)	44 (50.0)
Nurse-midwife	135 (49.3)	48 (52.7)	44 (46.3)	43 (48.9)
Midwife	5 (1.8)	0 (0.0)	5 (5.3)	0 (0.0)
**Country**				
Kenya	128 (46.7)	44 (48.4)	42 (44.2)	42 (47.7)
Nigeria	146 (53.3)	47 (51.6)	53 (55.8)	46 (52.3)
***Ward**				
Labor/delivery ward	188 (71.8)	62 (71.3)	63 (70.8)	63 (73.3)
Postnatal ward	48 (18.3)	15 (17.2)	15 (16.9)	18 (20.9)
Newborn unit/NICU	17 (6.5)	6 (6.9)	7 (7.9)	4 (4.7)
Operating theatre	9 (3.4)	4 (4.5)	4 (4.5)	1 (1.2)
****Post-training experience**				
< 5 years	65(23.9)	22(24.4)	17(18.1)	26(29.5)
5-10 years	83(30.5)	34(37.8)	24(25.5)	25(28.4)
11-15 years	40(14.7)	11(12.2)	16(17.0)	13(14.8)
16-20 years	32(11.8)	11(12.2)	13(13.8)	8(9.1)
> 20 years	52(19.1)	12(13.3)	24(25.5)	16(18.2)
**Prior HBB training**	52 (19.0)	16 (17.6)	23 (24.2)	13 (14.8)
****Owns a smartphone**	222 (92.5)	72 (91.2)	75 (91.5)	74 (94.9)

*Legend: *missing value=12; **missing value=34;*
NBU Newborn Unit; NICU Neonatal Intensive Care Unit

For peer review only - http://bmjopen.bmj.com/site/about/guidelines.xhtml

Neonatal resuscitation knowledge and skills on follow-up assessments

Neonatal resuscitation skills assessments were conducted at 1, 3 and 6 months after the in-person course. Differences in pass-rates on the BMV, OSCE A, and OSCE B checklists across groups on the 6-month post-course assessments were not statistically significant. Table 2.

Table 2. Comparison of 6-month post-course pass rates between groups

	Group n (%)				VR vs. Control			Video vs. Control		
**Variable**	**VR (n=87)**	**Video (n=92)**	**Control (N=86)**	**P-value***	**Δ**	**(95% CI)**	**P-value†**	**Δ**	**(95% CI)**	**P-value†**
BMV skill check	24 (28)	23 (25)	19 (22)	0.71	5.5%	(-8.5, 19.5%)	0.48	2.9%	(-10.7, 16.5%)	0.72
OSCE A	66 (76)	70 (76)	62 (72)	0.78	3.8%	(-10.5, 18.0%)	0.61	4.0%	(-10.0, 18.0%)	0.61
OSCE B	54 (62)	55 (60)	42 (49)	0.18	13.2 %	(-2.6, 29.1%)	0.093	10.9 %	(-4.7, 26.6%)	0.18

Legend: *Fisher's exact test comparing pass rates among the three groups;
†Fisher's exact test comparing pass rates of the VR or video group with the control group.

To determine whether pass-rates were impacted by years of experience, age, ward or prior HBB training, BMV skills, OSCE A and OSCE B pass-rates were compared between groups, one at a time. Participants with < 5 years of experience performed better on the OSCE A and OSCE B immediate post-course assessments (p=0.022 and p=0.034 respectively). Ward assignment to newborn unit (NBU/neonatal intensive care unit (NICU) or postnatal ward was also associated with greater immediate post-course pass-rate on BMV skills (p<0.001), OSCE A (p=0.001) and OSCE B assessments (p<0.001). At the 6-month follow-up assessment, there were no significant differences in BMV skills, OSCE A or OSCE B pass-rates by country site, years of experience, age, ward and HBB training > 1 year prior to the study. Table 3.

For peer review only

For peer review only - http://bmjopen.bmj.com/site/about/guidelines.xhtml

Table 3. Pass rates compared between demographic groups.

Variable	BMV Skills Check*		OSCE A*		OSCE B	
	Immediate	6-Month	Immediate	6-Month	Immediate	6-Month
**Years of Experience**						
<5 years	36 (62.1)	18 (28.6)	57 (87.7)	49 (77.8)	50 (76.9)	37 (58.7)
5-10 years	27 (41.5)	11 (16.4)	47 (65.3)	52 (77.6)	41 (56.9)	42 (62.7)
11-20 years	20 (44.4)	17 (32.7)	39 (75.0)	38 (73.1)	29 (55.8)	25 (48.1)
20+ years	35 (45.5)	20 (24.7)	62 (74.7)	58 (71.6)	57 (68.7)	46 (56.8)
**p-value†**	0.11	0.18	0.022	0.79	0.034	0.45
**Age**						
20-30 years	39 (60.0)	22 (31.0)	60 (83.3)	54 (76.1)	53 (73.6)	42 (59.2)
31-40 years	36 (45.0)	14 (16.7)	66 (75.9)	59 (70.2)	59 (67.8)	44 (52.4)
41-50 years	29 (42.0)	20 (26.0)	54 (66.7)	61 (79.2)	50 (61.7)	49 (63.6)
51+ years	14 (43.8)	10 (30.3)	26 (76.5)	24 (72.7)	16 (47.1)	16 (48.5)
**p-value†**	0.16	0.16	0.13	0.60	0.053	0.36
**Ward**						
Labor/delivery ward or operating theatre	77 (42.8)	49 (24.6)	147 (71.7)	150 (75.4)	120 (58.5)	113 (56.8)
NBU/NICU or postnatal ward	39 (70.9)	13 (24.1)	53 (93.0)	37 (68.5)	51 (89.5)	30 (55.6)
**p-value†**	<0.001	>0.99	0.001	0.30	<0.001	0.88
**Prior HBB training**						
Yes	22 (51.2)	14 (28.6)	40 (76.9)	38 (77.6)	29 (55.8)	29 (59.2)
No	96 (47.3)	52 (24.1)	166 (74.8)	160 (74.1)	149 (67.1)	122 (56.5)
**p-value†**	0.74	0.58	0.86	0.72	0.15	0.75

*Values are no (%) if not otherwise specified;
†Fisher's exact test comparing pass rates between groups.
NBU: Newborn Unit; NICU: Neonatal Intensive Care Unit

Neonatal resuscitation performance changes over time

There was a decline in performance on neonatal resuscitation skills assessments at 1 month across all groups with a variable degree of recovery of skills by the 3 and 6-month assessments. Figure 3.

Figure 3. Pass rates of BMV skills check, OSCE A and OSCE B assessments over time

For peer review only

For peer review only - http://bmjopen.bmj.com/site/about/guidelines.xhtml

BMV skill pass rates: BMV skills showed a decline at the 1-month assessment and remained significantly lower than the immediate post-course baseline in all groups at 3 months (-23% VR, p=0.001, -25% Video, p<0.001, -31%, p<0.001) and in the Video and Control groups at 6 months (-15% VR, p=0.10, -21% video, p<0.01, -27% control, p=0.001).

OSCE A pass rates: While pass rates decreased on the OSCE A assessments across all groups at 1-month, the groups improved over time and OSCE A pass-rates were close to the immediate post-course baseline at 6 months (-1% VR, -1% video, 0% control, p=0.83), with the VR group demonstrating an earlier recovery of skills (-2% VR, -9% video, -7% control, p=0.52 at 3 months). At 6 months, the VR group showed good performance on questions: *Prepares the area for ventilation* and *checks function of bag, mask and suction device* [VR 92%(n=87), Video 89%(n=92), Control 84%(n=86), p=0.25), *Recognizes baby is crying and breathing well* [VR 100%(n=87), Video 99%(n=92), Control 95%(n=86), p=0.07] and *Communicates with mother* [VR 94%(n=87), Video 86%(n=92), Control 86%(n=86), p=0.12] although these differences were not statistically significant.

OSCE B pass rates: OSCE B pass-rates were higher than the immediate post-course baseline at 3 and 6 months in the VR group (+4% at 3 months, p=0.64; +3% at 6 months, p=0.74) and lower in the Video (-21% at 3 months, p<0.001; -14% at 6 months, p=0.07) and Control groups (-7% at 3 months p=0.43; -14% at 6 months, p=0.10). Across groups, the performance was sustained on some items of the OSCE B skills checklist that are necessary to improve ventilation such as *Reapply mask* and *Reposition head*, while other recommended steps such as *Clear mouth and nose of secretions*, *Open the mouth and Squeeze bag harder*, showed a greater decline in

For peer review only - http://bmjopen.bmj.com/site/about/guidelines.xhtml

performance. See Figure 4. On post-hoc analysis of OSCE B assessments at the 6-month follow up, there was a statistically significant difference between the VR and Control groups on the frequency of performing the steps: *Opens mouth slightly* (54% vs. 37%, p=0.03) and *Squeezes bag harder* (75% vs. 59%, p=0.04). Differences in performance between the Video and Control group were not statistically significant: *Opens mouth slightly* (47% vs. 37%, p=0.23) and *Squeezes bag harder* (73% vs. 59%, p=0.06). The VR group performance on providing the target ventilation rate of 30-50 breaths per minute was better than the Control group (86% VR vs. 73% Control p=0.04). There was no statistically significant difference between the Video and Control groups on these metrics.

Figure 4. OSCE B skills performance on critical steps of ventilation

Participant exposure to interventions and clinical activities: Participants were assigned access to study interventions through the mobile Helping Babies Survive (mHBS) app and were encouraged to self-report HBB corner practice and deliveries assisted during the follow up period. The median user time spent in the mHBS Trainer app was 103(85-126) minutes. There was no difference between groups in number of minutes spent using the mHBS trainer 103(85-126) over the 6-month follow up period[VR 101(81-120), Video 108(87-133), Control 102(87-126), p=0.36]. There was no significant difference in self-reported clinical activities with median number of deliveries assisted [VR 25(5-64), Video 25(12-75), Control 28(7-108), p=0.51] and median deliveries requiring bag and mask ventilation [VR 9(3-20), Video 10(4-24), Control 9(4-26), p=0.67]. The median HBB corner practice days were also similar across groups [VR 16(6-42), Video 20(7-38), Control 16(7-51), p=0.86)].

For peer review only - http://bmjopen.bmj.com/site/about/guidelines.xhtml

Participant feedback: Participant feedback indicated overall positive impressions of the VR and video refresher training interventions. On a 6-month follow up survey with a Likert scale of 1-5 with 1 being strongly disagree and 5 being strongly agree, VR group participants agreed/strongly agreed that eHBB VR was easy to use (92%, n=82), realistic (90%, n=81) provided valuable clinical practice (92%, n=81) and feedback (90%, n=68). A majority of the Video group participants also agreed/strongly agreed that the video was easy to use (88%, n=83), realistic (96%, n=81) and valuable for clinical practice (85%, n=81). If given the opportunity, 95% (n=65) of VR and 98% (n=82) of video respondents would use their assigned intervention again.

DISCUSSION

This is the first randomized controlled trial that assesses the impact of mobile VR training for neonatal resuscitation skills retention in healthcare workers in a resource-scarce setting after standard in-person HBB training. Mobile VR training was highly feasible and acceptable to healthcare workers in a low- and middle-income country setting. Previous reports on neonatal resuscitation training using the HBB curriculum have demonstrated a decline in skills within months of training which may interfere with transfer of skills to clinical practice.[14-16 29-31] In this study of digital interventions for neonatal resuscitation skills retention, we found that digital interventions such as VR and video used for refresher training supported the retention of neonatal resuscitation skills in healthcare workers in Nigeria and Kenya. While the 6-month post-course performance was similar across groups, when compared with immediate post-course performance, the decreases in BMV skills pass-rates over time were significant in the video and control groups but not in the VR group. Also, contrary to the expected decline in performance

For peer review only - http://bmjopen.bmj.com/site/about/guidelines.xhtml

over time, OSCE B pass rates were higher at 3 and 6 months than immediately post-course in the VR group, suggesting that the VR training may provide additional support for the skills needed for prolonged neonatal resuscitation.

Both VR and video have been described for training in healthcare workers.[23 32-38] Video is a familiar medium but has been long considered a passive learning modality which should be combined with an active learning modality such as manikin-based practice or simulation.[21 39] Virtual simulation, an active learning modality, is thought to support learning through repetition, user engagement, and identity formation.[40 41] Although the overall performance of the video and VR groups was similar at the 6-month assessment, the VR group demonstrated an increased performance over time on some NR skills that have been demonstrated gaps in educational simulation-based settings such as the steps to improve bag and mask ventilation.[42] In settings where in-person refresher training is costly and potentially challenging, and may be even more difficult within the context of COVID-19 concerns, digital and telehealth interventions may adequately support the retention of neonatal resuscitation knowledge and skills.[43]

Little is known about the feasibility and acceptability of VR as a novel educational modality for training healthcare workers in resource-scarce settings. Previous descriptions of mobile VR use with school-age children in LMICs suggest that mobile VR simulations can be used to demonstrate real-world phenomena, illustrate abstract concepts, and motivate learners.[44] After using VR, students asked questions that reflected a deeper level of curiosity, engagement, and reflection on lesson topics. They also took ownership of the program by recharging mobile devices and creating their cardboard viewers.[44] Digital interventions such as VR may provide engaging, individualized, and incentivized practice opportunities.[37] A survey of healthcare workers' perspectives on simulation-based training in Nigeria showed a lack of awareness of VR

For peer review only - http://bmjopen.bmj.com/site/about/guidelines.xhtml

training, but willingness to utilize VR simulations if they were available.[45] Computer-based simulations have been used in high-resource settings as an adjunct to in-person neonatal and pediatric resuscitation training.[21] VR may support the transfer of knowledge to practice through interactive learning, problem-solving, and standardized feedback.[46 47] Connections that emerge between the participant's offline and in-game identity, and the actual interactions with virtual newborns and mothers within the VR simulation, may modify attitudes and behaviors that relate to clinical practice.[48-51] The healthcare workers in our study responded positively to mobile VR training.

Recently, Erdsal et al. described recommendations to improve the implementation of training programs like HBB by establishing a system for training HCWs and conducting low-dose high frequency (LDHF) practice that is tailored to needs, incentivized, and self-reflective.[52] This practice should emphasize both cognitive and psychomotor skills important for successful neonatal resuscitation. For practicing healthcare providers, the preparation of delivery and initial steps of resuscitation covered by OSCE A checklist are frequently performed in clinical practice, as 10% of babies will require some resuscitation. A number of digital innovations have been developed, over the past decade, to support *Helping Babies Breathe* education and training.[25 53 54] Based on our findings, after initial training, basic neonatal resuscitation skills may be supported by a range of digital training including VR, video, and even digital guide only. However, prolonged resuscitation (represented by the OSCE B scenario) occurs in only 1% of deliveries, so the performance of these skills is less common in clinical practice, particularly in low-volume healthcare facilities. Simulation practice is important for skills retention in healthcare workers at these facilities.[55] Pass rates on bag and mask skills were higher at 6 months in the VR group. Improvements in BMV performance over time were specifically seen in the critical skills needed

For peer review only - http://bmjopen.bmj.com/site/about/guidelines.xhtml

to improve ventilation in a baby who is not responding. Resuscitation actions to improve ventilation like *Opening the mouth* of the baby are notably hard to reinforce on manikin-based training because the manikin's mouth is designed to be always open.[56]

This study had several limitations. We only recruited participants from secondary and tertiary healthcare facilities. The study findings may not apply to HCWs who work at primary healthcare facilities or in community settings. Although a majority of the study participants reported owning a mobile phone, to ensure uniform access to the study interventions, all participants were provided a study phone to enable access to the digital resources. VR applications are compatible with a wide range of low-cost mobile phones, but not all phones can run VR applications. Participants were asked to access the digital interventions weekly, but the average frequency of access to the application was monthly. This likely reflects the difficulty of busy healthcare workers in finding time for in-service training, but it may have impacted the study findings.

CONCLUSION

Digital interventions supported the retention of neonatal resuscitation knowledge and skills for healthcare workers in Nigeria and Kenya. eHBB VR training was highly feasible and acceptable to healthcare workers in low-middle income countries. eHBB VR may provide additional support for neonatal resuscitation skills retention when compared to other digital interventions.

Acknowledgments: We would like to acknowledge the senior administrative and nursing staff at healthcare facilities that participated in the study. We acknowledge the input of Dr. Susan Niermeyer, Dr. Michael Visik and Dr. David Bolnick in developing the HBB 2nd edition video. We acknowledge the members of the University of Oxford LIFE Project who assisted in development of the eHBB VR application: Prof. Michael English, Dr. Niall Winters, Dr. Hilary Edgcombe, Mr. Jakob Rossner, Ms. Naomi Muinga (KEMRI-Wellcome Trust, Kenya) and the staff at National Hospital Abuja who assisted with eHBB VR testing: Dr. Amsa Mairami, Dr. Fatima Mairami, Dr. Adekunle Otuneye, Dr. Lamidi Audu, and Dr. Mariya Mukhtar-Yola.

For peer review only - http://bmjopen.bmj.com/site/about/guidelines.xhtml

Research assistants were provided with standardized *Helping Babies Breathe* 2nd Edition Facilitator training by Mr. Sammy O. Barasa and Mr. Geoffrey Mwai, of the Kenya *Helping Babies Survive* Master Trainer Corps. We would also like to acknowledge the study team members and collaborators who provided administrative, technical, and other support for the study: Ms. Oyin Akinrinola, Mr. Dillon Afenir, Mr. Bhavani Agnikula Kshatriya, Dr. Benjamin Al-Haddad, Mr. Iman Asangansi, Mr. Prem Avanigadda, Mr. Ariyo Ayokunle, Dr. Brian Bresnahan, Mr. Uchechukwu Chinedu, Mr. Emeka Chukwu, Mr. Chima Chukwudi, Ms. Bailey Clopp, Mr. Domnan Diretnan, Mr. Aniekan Ebito, Ms. Sakina Ginary, Ms. Chioma Ginikanwa, Mr. Oystein Gomo, Mr. Alex McGee, Mr. Manasseh Mmadu, Ms. Ajayi Motunrayo, Mr. Alvin Ogbonna, Mr. Kevin Otieno, Ms. Shruti Patel, Ms. Amanda Stiffler, Dr. Amy Thompson; *in memorium*, Dr. Charles Spiekerman and Ms. Elisabeth Meyers.

Contributorship Statement:

RU and SB conceptualized and designed the study, drafted the initial manuscript, designed the data collection tools, and reviewed and revised the manuscript.

CP and SP designed the data collection tools and reviewed and revised the manuscript.

BE, IF, FM, IA, MN, OO, CA, and AM collected data, and reviewed and revised the manuscript.

JF and DH carried out the initial analyses and reviewed and revised the manuscript.

CE and EE conceptualized and designed the study, collected data, and critically reviewed the manuscript for important intellectual content. All authors participated in the revision of the manuscript, approved the final version and agree to be accountable for all aspects of the work.

Funding/Support: This work was supported by the Bill & Melinda Gates Foundation, grant number OPP1169873.

Competing Interest: Dr. Umoren and Dr. Paton developed the eHBB VR application. Dr. Bucher and Dr. Purkayastha developed the mHBS/DHIS2 application. The other co-authors have no conflicts of interest relevant to this article to disclose.

Ethics approval: The study was approved by the University of Washington Institutional Review Board (IRB) approval number STUDY00005297, the Indiana University IRB approval number 1807371465, the Moi University, Health Research Ethics Committee approval number 0003109 and the University of Lagos College of Medicine Health Research Ethics Committee approval number CMUL/HREC/09/18/445.

Data availability statement: Data are available on request from Dr. Rachel Umoren at nestprog@uw.edu.

Clinical Trial Registration: Not required as the purpose of this study was to examine the effect only on the providers and not patients.

For peer review only - http://bmjopen.bmj.com/site/about/guidelines.xhtml

REFERENCES

1. UNICEF. Neonatal mortality: UNICEF; 2020 [Available from: https://data.unicef.org/topic/child-survival/neonatal-mortality/ accessed September 19 2020.
2. UNCF. United Nations Inter-agency Group for Child Mortality Estimation (UN IGME) Levels & Trends in Child Mortality: Report 2020, Estimates developed by the United Nations Inter-agency Group for Child Mortality Estimation. New York, 2020.
3. Chou D, Daelmans B, Jolivet RR, et al. Ending preventable maternal and newborn mortality and stillbirths. *BMJ* 2015;351:h4255. doi: 10.1136/bmj.h4255 [published Online First: 2015/09/16]
4. Sharma G, Mathai M, Dickson KE, et al. Quality care during labour and birth: a multi-country analysis of health system bottlenecks and potential solutions. *BMC Pregnancy Childbirth* 2015;15 Suppl 2(Suppl 2):S2. doi: 10.1186/1471-2393-15-S2-S2 [published Online First: 2015/09/24]
5. Enweronu-Laryea C, Dickson KE, Moxon SG, et al. Basic newborn care and neonatal resuscitation: a multi-country analysis of health system bottlenecks and potential solutions. *BMC Pregnancy Childbirth* 2015;15 Suppl 2(Suppl 2):S4. doi: 10.1186/1471-2393-15-S2-S4 [published Online First: 2015/09/24]
6. Lawn JE, Blencowe H, Oza S, et al. Every Newborn: progress, priorities, and potential beyond survival. *Lancet* 2014;384(9938):189-205. doi: 10.1016/S0140-6736(14)60496-7 [published Online First: 2014/05/24]
7. WHO. Standards for improving the quality of care for small and sick newborns in health facilities. 2020
8. Hedstrom A, Perez K, Umoren R, et al. Recent progress in global newborn health: thinking beyond acute to strategic care? *J Perinatol* 2019;39(8):1031-41. doi: 10.1038/s41372-019-0384-z [published Online First: 2019/06/12]
9. Hoban R, Bucher S, Neuman I, et al. 'Helping babies breathe' training in sub-saharan Africa: educational impact and learner impressions. *J Trop Pediatr* 2013;59(3):180-6. doi: 10.1093/tropej/fms077 [published Online First: 2013/01/22]
10. Kc A, Wrammert J, Clark RB, et al. Reducing Perinatal Mortality in Nepal Using Helping Babies Breathe. *Pediatrics* 2016;137(6) doi: 10.1542/peds.2015-0117 [published Online First: 2016/05/27]
11. Singhal N, Lockyer J, Fidler H, et al. Helping Babies Breathe: global neonatal resuscitation program development and formative educational evaluation. *Resuscitation* 2012;83(1):90-96. doi: 10.1016/j.resuscitation.2011.07.010 [published Online First: 07/19]
12. Msemo G, Massawe A, Mmbando D, et al. Newborn mortality and fresh stillbirth rates in Tanzania after helping babies breathe training. *Pediatrics* 2013;131(2):e353-60. doi: 10.1542/peds.2012-1795 [published Online First: 2013/01/23]
13. Versantvoort JMD, Kleinhout MY, Ockhuijsen HDL, et al. Helping Babies Breathe and its effects on intrapartum-related stillbirths and neonatal mortality in low-resource settings: a systematic review. *Arch Dis Child* 2020;105(2):127-33. doi: 10.1136/archdischild-2018-316319 [published Online First: 2019/07/07]

For peer review only - http://bmjopen.bmj.com/site/about/guidelines.xhtml

14. Eblovi D, Kelly P, Afua G, et al. Retention and use of newborn resuscitation skills following a series of helping babies breathe trainings for midwives in rural Ghana. *Glob Health Action* 2017;10(1):1387985. doi: 10.1080/16549716.2017.1387985 [published Online First: 2017/10/24]
15. Musafili A, Essen B, Baribwira C, et al. Evaluating Helping Babies Breathe: training for healthcare workers at hospitals in Rwanda. *Acta Paediatr* 2013;102(1):e34-8. doi: 10.1111/apa.12034 [published Online First: 2012/11/02]
16. Arlington L, Kairuki AK, Isangula KG, et al. Implementation of "Helping Babies Breathe": A 3-Year Experience in Tanzania. *Pediatrics* 2017;139(5) doi: 10.1542/peds.2016-2132 [published Online First: 2017/05/31]
17. Goudar SS, Somannavar MS, Clark R, et al. Stillbirth and newborn mortality in India after helping babies breathe training. *Pediatrics* 2013;131(2):e344-52. doi: 10.1542/peds.2012-2112 [published Online First: 2013/01/23]
18. Bookman L, Engmann C, Srofenyoh E, et al. Educational impact of a hospital-based neonatal resuscitation program in Ghana. *Resuscitation* 2010;81(9):1180-82.
19. Bang A, Patel A, Bellad R, et al. Helping Babies Breathe (HBB) training: What happens to knowledge and skills over time? *BMC Pregnancy Childbirth* 2016;16(1):364.
20. Atukunda IT, Conecker GA. Effect of a low-dose, high-frequency training approach on stillbirths and early neonatal deaths: a before-and-after study in 12 districts of Uganda. *The Lancet Global Health* 2017;5:S12.
21. Sawyer T, Umoren RA, Gray MM. Neonatal resuscitation: advances in training and practice. *Advances in medical education and practice* 2017;8:11-19. doi: 10.2147/AMEP.S109099 [published Online First: 2017/01/18]
22. Weinberg S, Jones D, Worku B, et al. Helping Babies Survive Training Programs: Evaluating a Teaching Cascade in Ethiopia. *Ethiop J Health Sci* 2019;29(6):669-76. doi: 10.4314/ejhs.v29i6.3 [published Online First: 2019/11/20]
23. Umoren R, Stadler DJ, Gasior SL, et al. Global collaboration and team-building through 3D virtual environments. *Innovations in Global Medical and Health Education* 2014:1.
24. Sitzmann T. A meta-analytic examination of the instructional effectiveness of computer-based simulation games. *Personnel psychology* 2011;64(2):489-528.
25. Bucher SL, Cardellichio P, Muinga N, et al. Digital health innovations, tools, and resources to support Helping Babies Survive programs. *Pediatrics* 2020;146(Supplement 2):S165-S82.
26. Kamath-Rayne BD, Thukral A, Visick MK, et al. Helping Babies Breathe, Second Edition: A Model for Strengthening Educational Programs to Increase Global Newborn Survival. *Glob Health Sci Pract* 2018;6(3):538-51. doi: 10.9745/GHSP-D-18-00147 [published Online First: 2018/10/06]
27. Umoren R. eHBB VR and eHBB mobile applications on Google play store, 2020.
28. Patterson JK, Girnary S, North K, et al. Innovations in cardiorespiratory monitoring to improve resuscitation with Helping Babies Breathe. *Pediatrics* 2020;146(Supplement 2):S155-S64.
29. Bang A, Patel A, Bellad R, et al. Helping Babies Breathe (HBB) training: What happens to knowledge and skills over time? *BMC Pregnancy Childbirth* 2016;16(1):364. doi: 10.1186/s12884-016-1141-3 [published Online First: 2016/11/24]
30. Cordova E, Al-Rousan T, Castillo-Angeles M, et al. Effect of low-cost interventions on the retention of knowledge and skills following Helping Babies Breathe training. *Int J*

For peer review only - http://bmjopen.bmj.com/site/about/guidelines.xhtml

Gynaecol Obstet 2018;142(2):248-54. doi: 10.1002/ijgo.12512 [published Online First: 2018/04/25]

31. Tabangin ME, Josyula S, Taylor KK, et al. Resuscitation skills after Helping Babies Breathe training: a comparison of varying practice frequency and impact on retention of skills in different types of providers. *Int Health* 2018;10(3):163-71. doi: 10.1093/inthealth/ihy017 [published Online First: 2018/04/05]
32. Tsai P-F, Kitch S, Beck C, et al. Using an Interactive Video Simulator to Improve Certified Nursing Assistants' Dressing Assistance and Nursing Home Residents' Dressing Performance: A Pilot Study. *Comput Inform Nurs* 2018;36(4):183-92. doi: 10.1097/CIN.0000000000000415
33. Curtis HA, Trang K, Chason KW, et al. Video-Based Learning vs Traditional Lecture for Instructing Emergency Medicine Residents in Disaster Medicine Principles of Mass Triage, Decontamination, and Personal Protective Equipment. *Prehosp Disaster Med* 2018;33(1):7-12. doi: 10.1017/S1049023X1700718X [published Online First: 01/10]
34. Coyne E, Frommolt V, Rands H, et al. Simulation videos presented in a blended learning platform to improve Australian nursing students' knowledge of family assessment. *Nurse Educ Today* 2018;66:96-102. doi: 10.1016/j.nedt.2018.04.012 [published Online First: 04/18]
35. Umoren RA, Gray MM, Schooley N, et al. Effect of Video-based Telemedicine on Transport Management of Simulated Newborns. *Air Med J* 2018;37(5):317-20. doi: 10.1016/j.amj.2018.05.007 [published Online First: 07/27]
36. Sweigart LI, Umoren RA, Scott PJ, et al. Virtual TeamSTEPPS((R)) Simulations Produce Teamwork Attitude Changes Among Health Professions Students. *J Nurs Educ* 2016;55(1):31-5. doi: 10.3928/01484834-20151214-08 [published Online First: 2016/01/27]
37. Sheik-Ali S, Edgcombe H, Paton C. Next-Generation virtual and augmented reality in surgical education: a narrative review. *Surg Technol Int* 2019;33
38. Muinga N, Paton C. Virtual reality for medical and nursing training in low-and middle-income countries. 2019
39. Komasawa N, Atagi K, Minami T. Combination of video and manikin hybrid simulation for rapid response emergency airway management training in medical students. *J Clin Anesth* 2018;46:75-76. doi: 10.1016/j.jclinane.2018.02.003 [published Online First: 02/03]
40. Umoren R, Rybas N. Who am I as a Healthcare Provider? *Integrating an Awareness of Selfhood and Society into Virtual Learning* 2017:166.
41. Gray MM, Thomas AA, Burns B, et al. Evacuation of Vulnerable and Critical Patients: Multimodal Simulation for Nurse-Led Patient Evacuation. *Simul Healthc* 2020 doi: 10.1097/SIH.0000000000000451 [published Online First: 2020/05/07]
42. Gaps in NRP Provider Performance on Standardized Simulations: A report from the NRP eSim™ Study. Pediatric Academic Societies Conference; 2019; Baltimore, MD.
43. Jones-Bamman C, Niermeyer S, McConnell K, et al. Teaching Helping Babies Breathe via Telehealth: A New Application in Rural Guatemala. *Biomed Hub* 2019;4(3):1-6. doi: 10.1159/000502934 [published Online First: 2020/01/30]
44. Examining Low-Cost Virtual Reality for Learning in Low-Resource Environments. Proceedings of the 2017 Conference on Designing Interactive Systems; 2017. ACM.
45. Umoren R, Ezeaka VC, Fajolu IB, et al. Perspectives on simulation-based training from paediatric healthcare providers in Nigeria: a national survey. *BMJ Open* 2020;10(2)

For peer review only - http://bmjopen.bmj.com/site/about/guidelines.xhtml

46. Sweigart LI, Umoren RA, Scott PJ, et al. Virtual TeamSTEPPS® Simulations Produce Teamwork Attitude Changes Among Health Professions Students. *Journal of Nursing Education* 2016;55(1):31-35.
47. Joynes C. Distance learning for health: what works-A global review of accredited post-qualification training programmes for health workers in low and middle Income Countries. *London Int Dev Cent* 2011;1
48. Waggoner Z. My avatar, my self: Identity in video role-playing games: McFarland 2009.
49. Parsons TD, Rizzo AA. Affective outcomes of virtual reality exposure therapy for anxiety and specific phobias: A meta-analysis. *Journal of behavior therapy and experimental psychiatry* 2008;39(3):250-61.
50. Seymour NE. VR to OR: a review of the evidence that virtual reality simulation improves operating room performance. *World J Surg* 2008;32(2):182-88.
51. Robb A, White C, Cordar A, et al. A comparison of speaking up behavior during conflict with real and virtual humans. *Comput Hum Behav* 2015;52(C):12-21. doi: 10.1016/j.chb.2015.05.043
52. Ersdal HL, Singhal N, Msemo G, et al. Successful implementation of Helping Babies Survive and Helping Mothers Survive programs—An Utstein formula for newborn and maternal survival. *PLoS One* 2017;12(6):e0178073.
53. Merali HS, Chan NH, Mistry N, et al. Designing and evaluating a novel mobile application for Helping Babies Breathe skills retention in Uganda: comparative study protocol. *BMJ Paediatr Open* 2019;3(1):e000561. doi: 10.1136/bmjpo-2019-000561 [published Online First: 2019/09/25]
54. Somannavar MS, Goudar SS, Revankar AP, et al. Evaluating time between birth to cry or bag and mask ventilation using mobile delivery room timers in India: the NICHD Global Network's Helping Babies Breathe Trial. *BMC Pediatr* 2015;15:93. doi: 10.1186/s12887-015-0408-6 [published Online First: 2015/08/08]
55. Lopreiato JO, Sawyer T. Simulation-based medical education in pediatrics. *Acad Pediatr* 2015;15(2):134-42. doi: 10.1016/j.acap.2014.10.010
56. Sawyer T, Strandjord T, Johnson K, et al. Neonatal airway simulators, how good are they? A comparative study of physical and functional fidelity. *Journal of Perinatology* 2016;36(2):151-56.

Figure 1. Study Diagram

Figure 2. CONSORT Diagram

Figure 3. Pass rates of BMV skills check, OSCE A and OSCE B assessments over time

Figure 3 Legend: Pass rates of BMV skills check, OSCE A, and OSCE B assessments over time. Immediate post-course, 1 month, 3 months, and 6 months assessments indicated by solid circles. *Statistically significant changes within each experimental group (VR, video, and control) from the immediate post-course assessment
†Significant changes from the 1-month assessment

Figure 4. OSCE B skills performance on critical steps of ventilation

For peer review only - http://bmjopen.bmj.com/site/about/guidelines.xhtml

Study group	Pre-course period			Follow-up period			
	Baseline assessment	Randomization to study intervention	Pre-course assessment	Immediate post-course	1 month post-course assessment	3 month post-course assessment	6 month post-course assessment
**VR**	Knowledge test	**VR + Digital HBB provider's guide**	Knowledge test, simulation performance	Knowledge test, simulation performance	Knowledge test, simulation performance	Knowledge test, simulation performance	Knowledge test, simulation performance
					**VR + LDHF manikin practice**		
**Video**	Knowledge test	**Video + Digital HBB provider's guide**	Knowledge test, simulation performance	Knowledge test, simulation performance	Knowledge test, simulation performance	Knowledge test, simulation performance	Knowledge test, simulation performance
					**Video + LDHF manikin practice**		
**Standard practice (control)**	Knowledge test	**Digital HBB provider's guide only**	Knowledge test, simulation performance	Knowledge test, simulation performance	Knowledge test, simulation performance	Knowledge test, simulation performance	Knowledge test, simulation performance
					**LDHF manikin practice only**		

VR: Virtual reality
BMV: bag-mask ventilation
LDHF: Low-dose high frequency

Figure 1. Study Diagram

165x100mm (300 x 300 DPI)

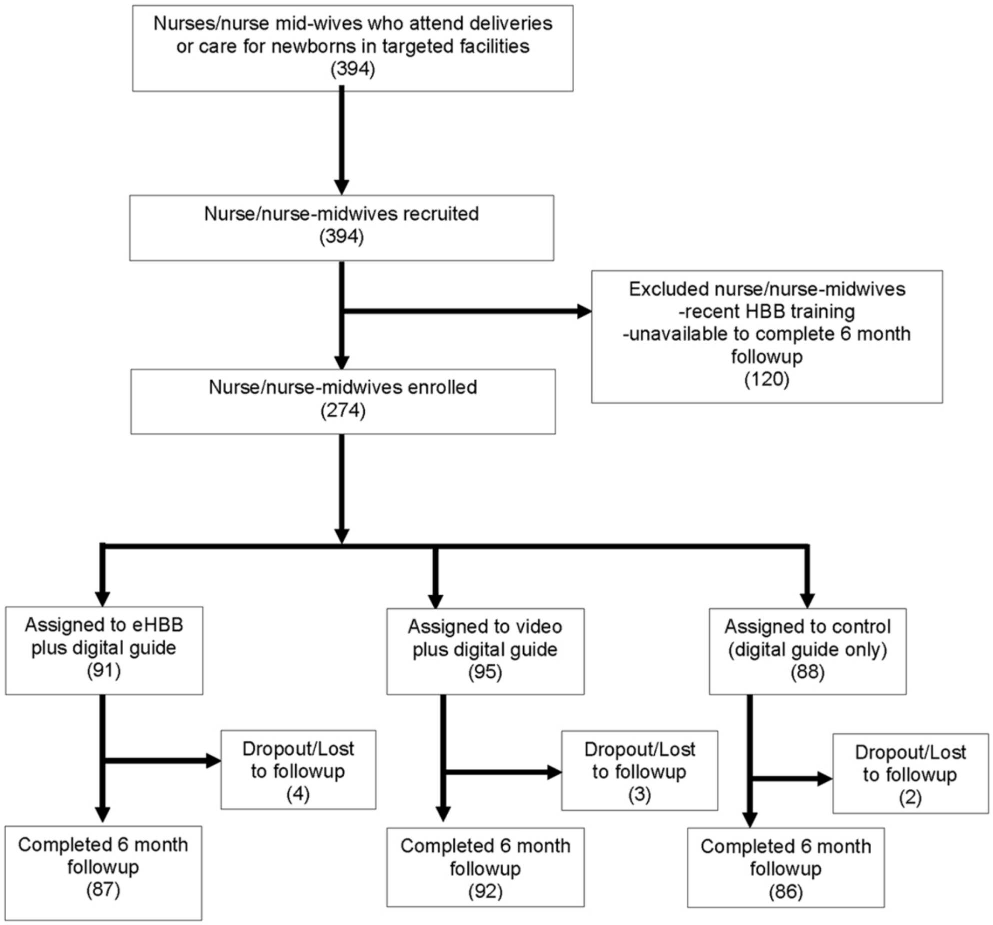

Figure 2. CONSORT Diagram

165x156mm (300 x 300 DPI)

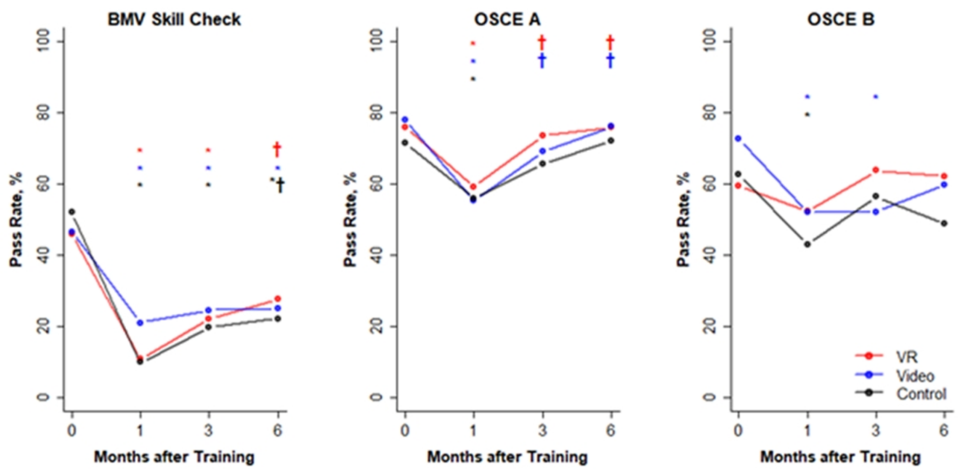

Figure 3. Pass-rates on BMV skills check, OSCE A and OSCE B assessments over time

165x81mm (300 x 300 DPI)

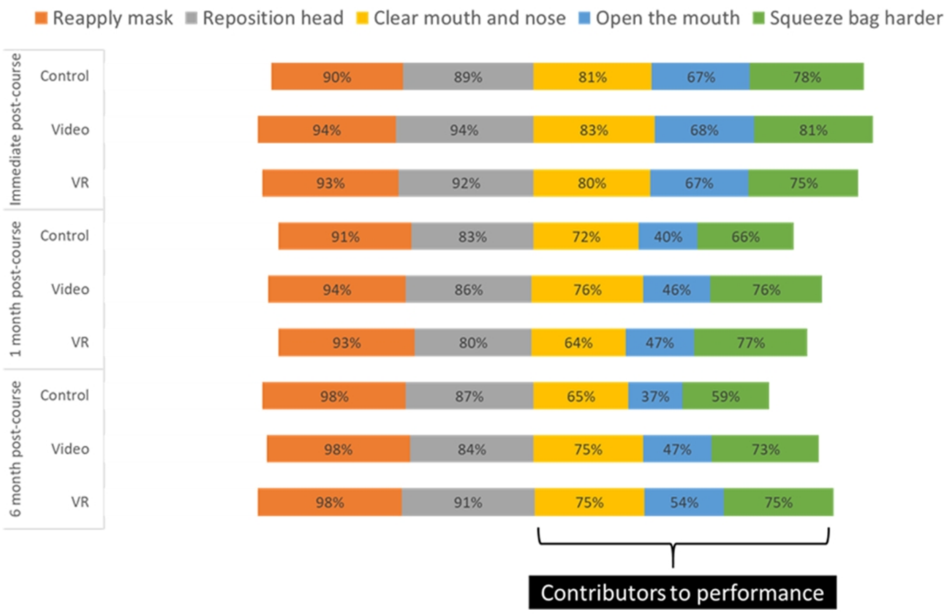

Figure 4. OSCE B skills performance on critical steps of ventilation

150x94mm (300 x 300 DPI)

CONSORT 2010 checklist of information to include when reporting a randomised trial*

Section/Topic	Item No	Checklist item	Reported on page No
**Title and abstract**			
	1a	Identification as a randomised trial in the title	1
	1b	Structured summary of trial design, methods, results, and conclusions (for specific guidance see CONSORT for abstracts)	2
**Introduction**			
Background and objectives	2a	Scientific background and explanation of rationale	3
	2b	Specific objectives or hypotheses	4
**Methods**			
Trial design	3a	Description of trial design (such as parallel, factorial) including allocation ratio	4
	3b	Important changes to methods after trial commencement (such as eligibility criteria), with reasons	N/A
Participants	4a	Eligibility criteria for participants	4
	4b	Settings and locations where the data were collected	4
Interventions	5	The interventions for each group with sufficient details to allow replication, including how and when they were actually administered	5-6
Outcomes	6a	Completely defined pre-specified primary and secondary outcome measures, including how and when they were assessed	5-6
	6b	Any changes to trial outcomes after the trial commenced, with reasons	N/A
Sample size	7a	How sample size was determined	7
	7b	When applicable, explanation of any interim analyses and stopping guidelines	N/A
Randomisation:			
Sequence generation	8a	Method used to generate the random allocation sequence	5
	8b	Type of randomisation; details of any restriction (such as blocking and block size)	5
Allocation concealment mechanism	9	Mechanism used to implement the random allocation sequence (such as sequentially numbered containers), describing any steps taken to conceal the sequence until interventions were assigned	5
Implementation	10	Who generated the random allocation sequence, who enrolled participants, and who assigned participants to interventions	5
Blinding	11a	If done, who was blinded after assignment to interventions (for example, participants, care providers, those	5

For peer review only

For peer review only - http://bmjopen.bmj.com/site/about/guidelines.xhtml

		assessing outcomes) and how	
	11b	If relevant, description of the similarity of interventions	N/A
Statistical methods	12a	Statistical methods used to compare groups for primary and secondary outcomes	7-8
	12b	Methods for additional analyses, such as subgroup analyses and adjusted analyses	7-8
**Results**			
Participant flow (a diagram is strongly recommended)	13a	For each group, the numbers of participants who were randomly assigned, received intended treatment, and were analysed for the primary outcome	8
	13b	For each group, losses and exclusions after randomisation, together with reasons	8
Recruitment	14a	Dates defining the periods of recruitment and follow-up	8
	14b	Why the trial ended or was stopped	8
Baseline data	15	A table showing baseline demographic and clinical characteristics for each group	9
Numbers analysed	16	For each group, number of participants (denominator) included in each analysis and whether the analysis was by original assigned groups	8
Outcomes and estimation	17a	For each primary and secondary outcome, results for each group, and the estimated effect size and its precision (such as 95% confidence interval)	9-14
	17b	For binary outcomes, presentation of both absolute and relative effect sizes is recommended	9-14
Ancillary analyses	18	Results of any other analyses performed, including subgroup analyses and adjusted analyses, distinguishing pre-specified from exploratory	9-14
Harms	19	All important harms or unintended effects in each group (for specific guidance see CONSORT for harms)	9-14
**Discussion**			
Limitations	20	Trial limitations, addressing sources of potential bias, imprecision, and, if relevant, multiplicity of analyses	17
Generalisability	21	Generalisability (external validity, applicability) of the trial findings	17
Interpretation	22	Interpretation consistent with results, balancing benefits and harms, and considering other relevant evidence	15-17
**Other information**			
Registration	23	Registration number and name of trial registry	23
Protocol	24	Where the full trial protocol can be accessed, if available	23
Funding	25	Sources of funding and other support (such as supply of drugs), role of funders	23

*We strongly recommend reading this statement in conjunction with the CONSORT 2010 Explanation and Elaboration for important clarifications on all the items. If relevant, we also recommend reading CONSORT extensions for cluster randomised trials, non-inferiority and equivalence trials, non-pharmacological treatments, herbal interventions, and pragmatic trials. Additional extensions are forthcoming: for those and for up to date references relevant to this checklist, see www.consort-statement.org.

For peer review only - http://bmjopen.bmj.com/site/about/guidelines.xhtml

CONSORT 2010 Flow Diagram

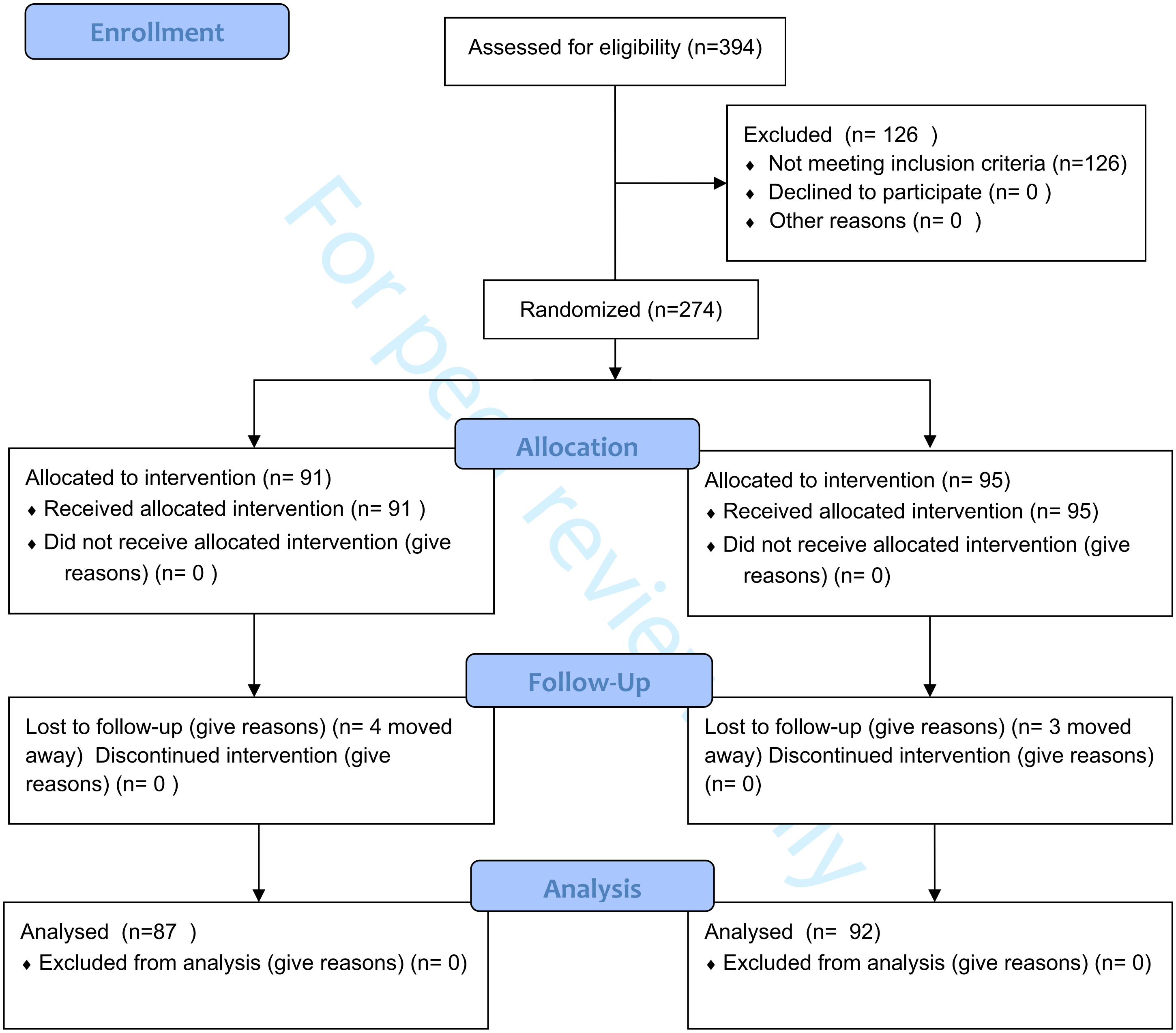

BMJ Open

eHBB: A randomized controlled trial of virtual reality or video for neonatal resuscitation refresher training in healthcare workers in resource scarce settings

Journal:	*BMJ Open*
Manuscript ID	bmjopen-2020-048506.R1
Article Type:	Original research
Date Submitted by the Author:	15-May-2021

Complete List of Authors:	Umoren, Rachel; University of Washington<br>Bucher, Sherri; Indiana University School of Medicine<br>Hippe, Daniel; University of Washington<br>Ezenwa, Beatrice; University of Lagos College of Medicine<br>Fajolu, Iretiola; University of Lagos College of Medicine<br>Okwako, Felicitas; Alupe University College<br>Feltner, John; University of Washington<br>Nafula, Mary; Alupe University College<br>Musale, Annet; Alupe University College<br>Olawuyi, Olubukola; University of Lagos College of Medicine<br>Adeboboye, Christianah; University of Lagos College of Medicine<br>Asangansi, Ime; eHealth4everyone<br>Paton, Chris; Nuffield Department of Clinical Medicine, Centre for Tropical Medicine<br>Purkayastha, Saptarshi; Indiana University Purdue University at Indianapolis<br>Ezeaka, Chinyere; University of Lagos College of Medicine<br>Esamai, Fabian; Alupe University College

**Primary Subject Heading**:	Medical education and training
Secondary Subject Heading:	Global health, Nursing, Paediatrics
Keywords:	PAEDIATRICS, Information technology < BIOTECHNOLOGY & BIOINFORMATICS, EDUCATION & TRAINING (see Medical Education & Training), Neonatal intensive & critical care < INTENSIVE & CRITICAL CARE, MEDICAL EDUCATION & TRAINING, NEONATOLOGY

SCHOLARONE™ Manuscripts

I, the Submitting Author has the right to grant and does grant on behalf of all authors of the Work (as defined in the below author licence), an exclusive licence and/or a non-exclusive licence for contributions from authors who are: i) UK Crown employees; ii) where BMJ has agreed a CC-BY licence shall apply, and/or iii) in accordance with the terms applicable for US Federal Government officers or employees acting as part of their official duties; on a worldwide, perpetual, irrevocable, royalty-free basis to BMJ Publishing Group Ltd ("BMJ") its licensees and where the relevant Journal is co-owned by BMJ to the co-owners of the Journal, to publish the Work in this journal and any other BMJ products and to exploit all rights, as set out in our licence.

The Submitting Author accepts and understands that any supply made under these terms is made by BMJ to the Submitting Author unless you are acting as an employee on behalf of your employer or a postgraduate student of an affiliated institution which is paying any applicable article publishing charge ("APC") for Open Access articles. Where the Submitting Author wishes to make the Work available on an Open Access basis (and intends to pay the relevant APC), the terms of reuse of such Open Access shall be governed by a Creative Commons licence – details of these licences and which Creative Commons licence will apply to this Work are set out in our licence referred to above.

Other than as permitted in any relevant BMJ Author's Self Archiving Policies, I confirm this Work has not been accepted for publication elsewhere, is not being considered for publication elsewhere and does not duplicate material already published. I confirm all authors consent to publication of this Work and authorise the granting of this licence.

For peer review only

eHBB: A randomized controlled trial of virtual reality or video for neonatal resuscitation refresher training in healthcare workers in resource scarce settings.

Rachel A. Umoren*, MB BCh, MS,[a] Sherri Bucher*, PhD, MA,[b] Daniel S. Hippe MS, [a] Beatrice Ezenwa, MBBS, MSc, MD,[c] Iretiola B. Fajolu, MBBS, [c] Felicitas M. Okwako, MB.ChB, MMED,[d] John Feltner, MS,[a] Mary Nafula, BSN, [d] Annet Musale, [d] Olubukola A. Olawuyi, BNSc, MSc, [c] Christianah O. Adeboboye, BNSc, [c] Ime Asangansi MBBS, PhD,[e] Chris Paton, BMBS, BMedSci, MBA,[f] Saptarshi Purkayastha, PhD,[g] Chinyere Ezeaka§, MBBS, MPH, [c] Fabian Esamai§, MB ChB.[d]

**Contributed equally as co-first authors*
§Contributed equally as co-senior authors

Affiliations:
[a]University of Washington, Seattle, Washington, U.S.A.
[b]Indiana University School of Medicine, Indianapolis, Indiana, U.S.A.
[c]College of Medicine, University of Lagos, Lagos, Nigeria
[d]Alupe University College, Busia, Kenya
[e]eHealth4everyone, Abuja, Nigeria
[f]University of Oxford, Oxford, U.K.
[g]Indiana University – Purdue University, Indianapolis, Indiana, U.S.A

Address correspondence to: Rachel A. Umoren, Department of Pediatrics, Division of Neonatology, 1959 NE Pacific St, Box 356320, Seattle, Washington, 98195, U.S.A. Telephone: 206-543-3200. Email address: rumoren@uw.edu

Text word count: 3639

Keywords: neonatology, nursing, pediatrics, medical education, information technology, virtual reality

For peer review only

ABSTRACT

Objective: To assess the impact of mobile virtual reality (VR) simulations using electronic Helping Babies Breathe (eHBB) or video for the maintenance of neonatal resuscitation skills in healthcare workers in resource scarce settings.

Design: Randomized controlled trial with 6-month follow-up (2018-2020).

Setting: Secondary and tertiary healthcare facilities.

Participants: 274 nurses and midwives assigned to labor and delivery, operating room, and newborn care units were recruited from 20 healthcare facilities in Nigeria and Kenya and randomized to one of three groups: VR (eHBB + digital guide), Video (video + digital guide), or Control (digital guide only) groups before an in-person HBB course.

Intervention(s): eHBB VR simulation or neonatal resuscitation video.

Main outcome(s): Healthcare worker neonatal resuscitation skills using standardized checklists in a simulated setting at 1 month, 3 months and 6 months.

Results: Neonatal resuscitation skills pass-rates were similar among the groups at 6-month follow-up for bag-and-mask ventilation (BMV) skills check (VR 28%, Video 25%, Control 22%, p=0.71), OSCE A (VR 76%, Video 76%, Control 72%, p=0.78) and OSCE B (VR 62%, Video 60%, Control 49%, p=0.18). Relative to the immediate post-course assessments, there was greater retention of BMV skills at 6 months in the VR group (-15% VR, p=0.10; -21% video, p<0.01, -27% control, p=0.001). OSCE B pass-rates in the VR group were numerically higher at 3 months (+4%, p=0.64) and 6 months (+3%, p=0.74) and lower in the Video (-21% at 3 months, p<0.001; -14% at 6 months, p=0.066) and Control groups (-7% at 3 months, p=0.43; -14% at 6 months, p=0.10). On follow-up survey, 95%(n=65) of respondents in the VR group and 98% (n=82) in the Video group would use their assigned intervention again.

Conclusion: eHBB VR training was highly acceptable to healthcare workers in low-middle income countries and may provide additional support for neonatal resuscitation skills retention compared to other digital interventions.

ARTICLE SUMMARY

Strengths and limitations of this study

- This study was a multi-center, randomized controlled trial of mobile virtual reality or video to support neonatal resuscitation skills retention in nurses and midwives who provide neonatal resuscitation in two low-middle income countries.
- The study used an evidence-based Helping Babies Breathe 2nd edition curriculum designed for neonatal resuscitation training in resource-scarce settings.
- Healthcare workers accessed the digital interventions on mobile phones in the 6 months following in-person Helping Babies Breathe training.
- Healthcare workers were recruited from secondary and tertiary healthcare settings in urban and semi-urban resource-scarce settings, so the study findings may not apply to healthcare workers in high resource, primary healthcare, or rural settings.

For peer review only - http://bmjopen.bmj.com/site/about/guidelines.xhtml

INTRODUCTION

In 2019, there were 2.4 million deaths among infants under 28 days of age.[1 2] These neonatal deaths now account for 47% of global under 5-years child mortality,[2] and most cases are preventable.[3-5] The majority of these deaths occur in low-middle income countries (LMICs). Nigeria, with a neonatal mortality rate of 36 deaths per 1000 live births and Kenya at 21 deaths per 1000 live births in 2019,[1] are at significant risk of failing to meet the United Nations Sustainable Development Goal 3 (SDG 3) to reduce neonatal mortality to 12 per 1,000 live births by 2030.[8]

Intrapartum asphyxia or lack of breathing at birth is a leading cause of neonatal mortality.[6 7] Training healthcare workers in neonatal resuscitation using the *Helping Babies Breathe* (HBB) curriculum builds competency and reduces newborn morbidity and mortality.[8-13] However, neonatal resuscitation skills are quickly lost after trainings that utilize the traditional approach of small group facilitated classroom training.[14-19] For this reason, periodic refresher training with "low-dose high frequency" manikin-based simulations are recommended to support neonatal resuscitation skills retention.[20 21] Unfortunately, access to manikin-based simulation is limited by trainer, space, and equipment availability.[22 23] Yet, the high penetration of smartphones and cellular network connectivity in urban and rural areas in LMICs, makes innovative simulation training feasible using mobile virtual reality (VR) simulations for healthcare workers (HCW) who provide care in health facility and community-based settings.

VR simulations are effective educational tools and can be engaged at the learner's convenience, on their own smartphone, with game-based automated feedback that is ideal for episodic learning.[24-26] However little is known of their feasibility, acceptability, or effectiveness for

For peer review only - http://bmjopen.bmj.com/site/about/guidelines.xhtml

neonatal resuscitation skills retention in LMICs. We hypothesized that mobile VR simulation refresher training would address challenges to the quality of newborn resuscitation related to the maintenance of HCW knowledge and skills over time addressing the lack of standardized dissemination of updates to recommended practice and high rates of staff turnover. The objective of this study was to evaluate the impact of eHBB VR used with in-person neonatal resuscitation training on neonatal resuscitation educational indicators and performance outcomes, in comparison to other digital refresher training modalities.

METHODS

Study setting: The study was conducted in Lagos, Nigeria and Busia, Western Kenya. Twelve healthcare facilities (9 secondary and 3 tertiary) were located in Nigeria while 8 facilities were located in Kenya. The healthcare facilities were located in urban and semi-urban areas and all have maternal and newborn services with newborn bed capacity ranging from 2-80 beds and delivery and neonatal unit staffing capacity from 7-124 nurses. The study was approved by the University of Washington Institutional Review Board (IRB), the Indiana University IRB, the Moi University, Health Research Ethics Committee and the University of Lagos College of Medicine Health Research Ethics Committee.

Participants: Study participants consisted of nurses and nurse-midwives assigned to labor and delivery, operating room, and newborn care units. Site coordinators or research assistants requested contact numbers, units, and wards of potential participants from head nurses at identified facilities. Research assistants contacted individuals to determine eligibility and obtained consent.

Inclusion Criteria: Nurses and midwives who participate in deliveries and who provide neonatal resuscitation to inborn or outborn infants and provide study consent.

For peer review only

For peer review only - http://bmjopen.bmj.com/site/about/guidelines.xhtml

Exclusion Criteria: Those who had attended a neonatal resuscitation training course in the 1 year preceding the study; individuals who did not provide neonatal resuscitation as part of their duties or would be unavailable or unwilling to participate in follow-up study activities throughout the 6-month post-initial training period.

Randomization: Study IDs generated for each country site were randomly assigned via a computer-generated algorithm to the VR, Video, and Control groups by a U.S. based study coordinator. Participants were enrolled and assigned a study ID before the HBB course by local study coordinators. Each participant received an Android study phone, pre-loaded with permission-based access linked to their study ID, via the mobile Helping Babies Survive powered by District Health Information System (DHIS2) app (mHBS/DHIS2), to the participant's assigned digital intervention. The data analysis team was blinded to the study assignments.

HBB course structure: The HBB provider course (Second Edition)[27] was taught by study HBB master trainers as one-day, 8-hour long sessions from December 2018-August 2019. A 30-minute orientation was provided on use of the mHBS/DHIS2 app, including how to access the assigned digital intervention. All participants had access to a digitized HBB provider manual through the mHBS/DHIS2 app. The VR group in addition accessed the eHBB VR simulations which consisted of three interactive 3D simulation scenarios representing care of a newborn requiring routine care, some resuscitation and prolonged resuscitation with positive pressure ventilation. The features of eHBB VR have been previously described and the application is available for free download.[26 28] The neonatal resuscitation video used by the video group

For peer review only - http://bmjopen.bmj.com/site/about/guidelines.xhtml

featured preparation for delivery and the resuscitation of a newborn requiring positive pressure ventilation.[29] None of the interventions required internet for use. A total of 274 healthcare workers participated in the in-person Helping Babies Breathe training.

Pre- and post-course assessments: Standardized knowledge and skills assessments were conducted by trained research assistants. The HBB Knowledge Check (15 of 18 multiple-choice questions, ≥80% required to pass) and Bag-and Mask Ventilation Skill Check (BMV; 14 of 14 items required to pass) were conducted pre- and post-course along with the Objective Structured Clinical Examination (OSCE) A checklist on preparation for delivery and initial steps of resuscitation (9 out of 12 items and 3 required items to pass). In addition, the post-course assessment included the OSCE B checklist on prolonged newborn resuscitation (17 out of 23 items and 6 required items to pass). HBB checklists are available for free download from the American Academy of Pediatrics.[30] A demographic survey was completed. See Figure 1.

Figure 1. Study Design

Post-course interventions and follow-up: Following the course, participants were encouraged to use their assigned digital intervention weekly and to engage in standard bag-and-mask skills practice with a manikin at the HBB practice corner set up at their facility. Post-course assessments were repeated at 1, 3, and 6 months after the class. A follow-up survey was completed.

For peer review only - http://bmjopen.bmj.com/site/about/guidelines.xhtml

Data collection: Data were collected in person by study staff who had completed a HBB 2nd edition master trainer course by experienced HBB master trainers. Staff used the mHBS/DHIS2 Tracker app for offline data collection[26]. The mHBS Tracker app contained digitized HBB knowledge check, BMV skill check, and OSCE A and OSCE B checklist and was used by the participants to report their HBB corner practice. The mHBS Trainer app separately tracked educational interventions access and use. To standardize data collection and feedback to study participants, an enhanced neonatal simulator, called *NeoNatalie Live* [Laerdal Medical] was used for bag-and-mask ventilation. Compared to the low-fidelity *NeoNatalie* simulators used for HBB training (including the HBB practice corners in this study), *NeoNatalie Live* manikin can be programmed to simulate key physiological parameters, such as variable rates of lung stiffness and heart rate and provides auditory and visual cues, in the form of "crying" and increased heart rate when the end-user provides bag-and-mask ventilation[31]. In addition, brief automated feedback for the end-user is provided using a Bluetooth-connected tablet device at the end of the assessment as "well done" or "needs improvement" based on bag and mask performance. The use of the *NeoNatalie Live* manikin software enabled the correlation of observer collected metrics with manikin collected data. The automated feedback provided by *Neonatalie Live* was the only feedback provided following each assessment.[31]

Patients or public involvement: Patients or the public were not involved in the design, or conduct, or reporting, or dissemination plans of our research.

Sample size calculations: We hypothesized that there will be at least a 20% difference in the proportion of subjects who pass OSCE B at the 6-month evaluation between the VR group or Video group and the Control group. A sample size of 83 subjects per group would provide 80%

For peer review only

For peer review only - http://bmjopen.bmj.com/site/about/guidelines.xhtml

power to detect a difference in pass rates between groups if the true pass rates were 85% and 65%, respectively, based on a two-sided $\alpha = 0.05$. The required total sample size for the three groups (VR, Video, and Control) was 249. We recruited 274 participants total to allow for 10% dropout over the 6-month follow-up period.

Data Analysis: An intention-to-treat analysis was performed, where participants were grouped according to their randomly allocated experimental group (VR, Video, or Control) regardless of their actual exposure. Fisher's exact test was used to test for any differences in pass rates among the three groups for each of the study evaluations: Bag-and-mask ventilation skills assessment (BMV), and standardized simulations of routine care and initial resuscitation (OSCE A) and prolonged resuscitation (OSCE B). Post-hoc pairwise comparisons and comparisons between demographic groups were also performed using Fisher's exact test. Within-group comparisons of evaluation results between timepoints were performed using the Sign test. Participant exposure to the interventions (time in the mHBS Trainer app) and self-reported clinical activity during the follow-up period were compared between experimental groups using the Kruskal-Wallis test and Wilcoxon rank-sum test. All statistical calculations were conducted with the statistical computing language R (version 4.0.0; R Foundation for Statistical Computing, Vienna, Austria). Throughout, two-sided tests were used, with statistical significance defined as $p<0.05$.

RESULTS

Recruitment, training and follow-up were conducted concurrently at participating sites from December 2018 to August 2019 with follow-up continuing until February 2020. Of the 394 nurses and nurse-midwives identified who attended deliveries at the participating sites, 274

For peer review only - http://bmjopen.bmj.com/site/about/guidelines.xhtml

consented to participate in the study. Of the 274 participants, 265 (97%) completed a 6-month assessment, with a similar dropout rate in each group (p=0.52 for the difference between groups, Figure 2).

Figure 2. CONSORT Diagram

Most participants were female (91%), nurse-midwives or midwives (51%), who worked in the labor and delivery ward (72%). Nearly all owned a smartphone (Table 1). Neonatal resuscitation knowledge and skills assessments were conducted immediately after the in-person course. There were no differences in Knowledge check scores [VR 18(17-18), Video 18(17-18), Control 18(17-18), p 0.76] or pass-rates on the BMV [VR 46%(n=83), Video 46%(n=84), Control 52%(n=79), p=0.72], OSCE A [VR 76%(n=91), 78%(n=95), 72%(n=88), p=0.63] and OSCE B [VR 59%(n=91), Video 73%(n=95), Control 62%(n=88), 0.13] assessments across groups on the immediate post-course assessments.

Table 1 Demographics of study participants

**Demographics**	**All**<br>n = 274<br>n (%)	**VR + Digital Guide**<br>n=91<br>n (%)	**Video + Digital Guide**<br>n=95<br>n (%)	**Digital HBB Guide only**<br>n=88<br>n (%)
**Age,** years, mean(SD)	38 (9)	37(9)	41(10)	37 (9)
**Gender**				
Female	250 (91.2)	82 (90.1)	88 (92.6)	80 (90.9)
***Profession**				
Nurse	133 (48.5)	43 (47.3)	46 (48.4)	44 (50.0)
Nurse-midwife	135 (49.3)	48 (52.7)	44 (46.3)	43 (48.9)
Midwife	5 (1.8)	0 (0.0)	5 (5.3)	0 (0.0)

For peer review only - http://bmjopen.bmj.com/site/about/guidelines.xhtml

**Country**				
Kenya	128 (46.7)	44 (48.4)	42 (44.2)	42 (47.7)
Nigeria	146 (53.3)	47 (51.6)	53 (55.8)	46 (52.3)
****Ward**				
Labor/delivery ward	188 (71.8)	62 (71.3)	63 (70.8)	63 (73.3)
Postnatal ward	48 (18.3)	15 (17.2)	15 (16.9)	18 (20.9)
Newborn unit/NICU	17 (6.5)	6 (6.9)	7 (7.9)	4 (4.7)
Operating theatre	9 (3.4)	4 (4.5)	4 (4.5)	1 (1.2)
*****Post-training experience**				
< 5 years	65(23.9)	22(24.4)	17(18.1)	26(29.5)
5-10 years	83(30.5)	34(37.8)	24(25.5)	25(28.4)
11-15 years	40(14.7)	11(12.2)	16(17.0)	13(14.8)
16-20 years	32(11.8)	11(12.2)	13(13.8)	8(9.1)
> 20 years	52(19.1)	12(13.3)	24(25.5)	16(18.2)
**Prior HBB training**	52 (19.0)	16 (17.6)	23 (24.2)	13 (14.8)
****Owns a smartphone**	222 (92.5)	72 (91.2)	75 (91.5)	74 (94.9)

*Legend: *missing value =1; **missing value=12; ***missing value=34; NICU Neonatal Intensive Care Unit*

Neonatal resuscitation knowledge and skills on follow-up assessments

Neonatal resuscitation skills assessments were conducted at 1, 3 and 6 months after the in-person course. Differences in pass-rates on the BMV, OSCE A, and OSCE B checklists across groups on the 6-month post-course assessments were not statistically significant. Table 2.

Table 2. Comparison of 6-month post-course pass rates between groups

	Group n (%)				VR vs. Control			Video vs. Control		
**Variable**	**VR (n=87)**	**Video (n=92)**	**Control (N=86)**	**P-value***	**Δ**	**(95% CI)**	**P-value†**	**Δ**	**(95% CI)**	**P-value†**
BMV skill check	24 (28)	23 (25)	19 (22)	0.71	5.5%	(-8.5, 19.5%)	0.48	2.9%	(-10.7, 16.5%)	0.72

For peer review only

OSCE A	66 (76)	70 (76)	62 (72)	0.78	3.8%	(-10.5, 18.0%)	0.61	4.0%	(-10.0, 18.0%)	0.61
OSCE B	54 (62)	55 (60)	42 (49)	0.18	13.2%	(-2.6, 29.1%)	0.09	10.9 %	(-4.7, 26.6%)	0.18

Legend: *Fisher's exact test comparing pass rates among the three groups;
†Fisher's exact test comparing pass rates of the VR or video group with the control group.

To determine whether pass-rates were impacted by years of experience, age, profession, ward or prior HBB training, BMV skills, OSCE A and OSCE B pass-rates were compared between groups, one at a time. Participants with < 5 years of experience performed better on the OSCE A and OSCE B immediate post-course assessments (p=0.022 and p=0.034 respectively). Nurse-midwives performed better on BMV skills (p<0.001), OSCE A (p<0.001), and OSCE B immediate post-course assessments (p=0.011) compared to nurses, though pass rates were similar for all three tests at 6 months (p = 0.14 - 0.89). Ward assignment to newborn unit, neonatal intensive care unit (NICU) or postnatal ward was also associated with greater immediate post-course pass-rate on BMV skills (p<0.001), OSCE A (p=0.001) and OSCE B assessments (p<0.001). At the 6-month follow-up assessment, there were no significant differences in BMV skills, OSCE A or OSCE B pass-rates by country site, years of experience, age, ward and HBB training > 1 year prior to the study. Table 3.

Table 3. Pass rates compared between demographic groups.

Demographic	BMV Skills Check*		OSCE A*		OSCE B*	
	Immediate n (%)	6-Month n (%)	Immediate n (%)	6-Month n (%)	Immediate n (%)	6-Month n (%)
**Years of Experience**						
<5 years	36 (62.1)	18 (28.6)	57 (87.7)	49 (77.8)	50 (76.9)	37 (58.7)

For peer review only

For peer review only - http://bmjopen.bmj.com/site/about/guidelines.xhtml

5-10 years	27 (41.5)	11 (16.4)	47 (65.3)	52 (77.6)	41 (56.9)	42 (62.7)
11-20 years	20 (44.4)	17 (32.7)	39 (75.0)	38 (73.1)	29 (55.8)	25 (48.1)
20+ years	35 (45.5)	20 (24.7)	62 (74.7)	58 (71.6)	57 (68.7)	46 (56.8)
**p-value†**	0.11	0.18	0.02	0.79	0.03	0.45
**Age**						
20-30 years	39 (60.0)	22 (31.0)	60 (83.3)	54 (76.1)	53 (73.6)	42 (59.2)
31-40 years	36 (45.0)	14 (16.7)	66 (75.9)	59 (70.2)	59 (67.8)	44 (52.4)
41-50 years	29 (42.0)	20 (26.0)	54 (66.7)	61 (79.2)	50 (61.7)	49 (63.6)
51+ years	14 (43.8)	10 (30.3)	26 (76.5)	24 (72.7)	16 (47.1)	16 (48.5)
**p-value†**	0.16	0.16	0.13	0.60	0.05	0.36
Profession						
Nurse	31 (27.7)	34 (26.6)	87 (65.4)	95 (74.2)	76 (57.1)	79 (61.7)
Nurse-midwife	87 (64.9)	32 (23.4)	119 (84.4)	103 (75.2)	102 (72.3)	72 (52.6)
**p-value†**	<0.001	0.57	<0.001	0.89	0.01	0.14
**Ward**						
Labor/delivery ward or operating theatre	77 (42.8)	49 (24.6)	147 (71.7)	150 (75.4)	120 (58.5)	113 (56.8)
NBU/NICU or postnatal ward	39 (70.9)	13 (24.1)	53 (93.0)	37 (68.5)	51 (89.5)	30 (55.6)
**p-value†**	<0.001	>0.99	0.001	0.30	<0.001	0.88
**Prior HBB training**						
Yes	22 (51.2)	14 (28.6)	40 (76.9)	38 (77.6)	29 (55.8)	29 (59.2)
No	96 (47.3)	52 (24.1)	166 (74.8)	160 (74.1)	149 (67.1)	122 (56.5)
**p-value†**	0.74	0.58	0.86	0.72	0.15	0.75

*Values are no (%) if not otherwise specified;
†Fisher's exact test comparing pass rates between groups.
NBU: Newborn Unit; NICU: Neonatal Intensive Care Unit

Neonatal resuscitation performance changes over time

There was a decline in performance on neonatal resuscitation skills assessments at 1 month across all groups with a variable degree of recovery of skills by the 3 and 6-month assessments. Figure 3.

Figure 3. Pass rates of BMV skills check, OSCE A and OSCE B assessments over time

For peer review only

For peer review only - http://bmjopen.bmj.com/site/about/guidelines.xhtml

BMV skill pass rates: BMV skills showed a decline at the 1-month assessment and remained significantly lower than the immediate post-course baseline in all groups at 3 months (-23% VR, p=0.001, -25% Video, p<0.001, -31%, p<0.001) and in the Video and Control groups at 6 months (-15% VR, p=0.10, -21% video, p<0.01, -27% control, p=0.001).

OSCE A pass rates: While pass rates decreased on the OSCE A assessments across all groups at 1-month, the groups improved over time and OSCE A pass-rates were close to the immediate post-course baseline at 6 months (-1% VR, -1% video, 0% control, p=0.83), with the VR group demonstrating an earlier recovery of skills (-2% VR, -9% video, -7% control, p=0.52 at 3 months). At 6 months, the VR group showed good performance on questions: *Prepares the area for ventilation* and *checks function of bag, mask and suction device* [VR 92%(n=87), Video 89%(n=92), Control 84%(n=86), p=0.25), *Recognizes baby is crying and breathing well* [VR 100%(n=87), Video 99%(n=92), Control 95%(n=86), p=0.07] and *Communicates with mother* [VR 94%(n=87), Video 86%(n=92), Control 86%(n=86), p=0.12] although these differences were not statistically significant.

OSCE B pass rates: OSCE B pass-rates were higher than the immediate post-course baseline at 3 and 6 months in the VR group (+4% at 3 months, p=0.64; +3% at 6 months, p=0.74) and lower in the Video (-21% at 3 months, p<0.001; -14% at 6 months, p=0.07) and Control groups (-7% at 3 months p=0.43; -14% at 6 months, p=0.10). Across groups, the performance was sustained on some items of the OSCE B skills checklist that are necessary to improve ventilation such as *Reapply mask* and *Reposition head*, while other recommended steps such as *Clear mouth and nose of secretions*, *Open the mouth and Squeeze bag harder*, showed a greater decline in

For peer review only

For peer review only - http://bmjopen.bmj.com/site/about/guidelines.xhtml

performance. See Figure 4. On post-hoc analysis of OSCE B assessments at the 6-month follow up, there was a statistically significant difference between the VR and Control groups on the frequency of performing the steps: *Opens mouth slightly* (54% vs. 37%, p=0.03) and *Squeezes bag harder* (75% vs. 59%, p=0.04) and providing the target *ventilation rate of 30-50 breaths per minute* (86% VR vs. 73% Control, p=0.04). Differences in performance between the Video and Control group were not statistically significant on these metrics: *Opens mouth slightly* (47% vs. 37%, p=0.23),*Squeezes bag harder* (73% vs. 59%, p=0.06), and *ventilation rate of 30-50 breaths per minute* (85% Video vs. 73% Control, p=0.06).

Figure 4. OSCE B skills performance on critical steps of ventilation

Participant exposure to interventions and clinical activities: Participants were assigned access to study interventions through the mobile Helping Babies Survive (mHBS) app and were encouraged to self-report HBB corner practice and deliveries assisted during the follow up period. The median user time spent in the mHBS Trainer app was 103(85-126) minutes. This software reported metric reflected the time spent accessing the educational interventions in all groups. There was no difference between groups in number of minutes spent using the mHBS trainer 103(85-126) over the 6-month follow up period [VR 101(81-120), Video 108(87-133), Control 102(87-126), p=0.36]. There was no significant difference in self-reported clinical activities with median number of deliveries assisted [VR 25(5-64), Video 25(12-75), Control 28(7-108), p=0.51] and median deliveries requiring bag and mask ventilation [VR 9(3-20), Video 10(4-24), Control 9(4-26), p=0.67]. The median HBB corner practice days were also similar across groups [VR 16(6-42), Video 20(7-38), Control 16(7-51), p=0.86)].

For peer review only - http://bmjopen.bmj.com/site/about/guidelines.xhtml

Participant feedback: Participant feedback indicated overall positive impressions of the VR and video refresher training interventions. On a 6-month follow up survey with a Likert scale of 1-5 with 1 being strongly disagree and 5 being strongly agree, VR group participants agreed/strongly agreed that eHBB VR was easy to use (92%, n=82), realistic (90%, n=81) provided valuable clinical practice (92%, n=81) and feedback (90%, n=68). A majority of the Video group participants also agreed/strongly agreed that the video was easy to use (88%, n=83), realistic (96%, n=81) and valuable for clinical practice (85%, n=81). If given the opportunity, 95% (n=65) of VR and 98% (n=82) of video respondents would use their assigned intervention again.

DISCUSSION

This is the first randomized controlled trial that assesses the impact of mobile VR training for neonatal resuscitation skills retention in healthcare workers in a resource-scarce setting after standard in-person HBB training. Mobile VR training was highly feasible and acceptable to healthcare workers in a low- and middle-income country setting. Previous reports on neonatal resuscitation training using the HBB curriculum have demonstrated a decline in skills within months of training which may interfere with transfer of skills to clinical practice.[14-16 32 33] In this study of digital interventions for neonatal resuscitation skills retention, we found that digital interventions such as VR and video used for refresher training supported the retention of neonatal resuscitation skills in healthcare workers in Nigeria and Kenya. While the 6-month post-course performance was similar across groups, when compared with immediate post-course performance, the decreases in BMV skills pass-rates over time were significant in the video and control groups but not in the VR group. Also, contrary to the expected decline in performance

For peer review only

For peer review only - http://bmjopen.bmj.com/site/about/guidelines.xhtml

over time, OSCE B pass rates were higher at 3 and 6 months than immediately post-course in the VR group, suggesting that the VR training may provide additional support for the skills needed for prolonged neonatal resuscitation.

Both VR and video have been described for training in healthcare workers.[24 34-40] Video is a familiar medium but has been long considered a passive learning modality which should be combined with an active learning modality such as manikin-based practice or simulation.[22 41] Virtual simulation, an active learning modality, is thought to support learning through repetition, user engagement, and identity formation.[42 43] Although the overall performance of the video and VR groups was similar at the 6-month assessment, the VR group demonstrated an increased performance over time on some neonatal resuscitation skills that have been demonstrated gaps in educational simulation-based settings such as the steps to improve bag and mask ventilation.[44] In settings where in-person refresher training is costly and potentially challenging, and may be even more difficult within the context of COVID-19 concerns, digital and telehealth interventions may adequately support the retention of neonatal resuscitation knowledge and skills.[45]

Little is known about the feasibility and acceptability of VR as a novel educational modality for training healthcare workers in resource-scarce settings. Previous descriptions of mobile VR use with school-age children in LMICs suggest that mobile VR simulations can be used to demonstrate real-world phenomena, illustrate abstract concepts, and motivate learners.[46] After using VR, students asked questions that reflected a deeper level of curiosity, engagement, and reflection on lesson topics. They also took ownership of the program by recharging mobile devices and creating their cardboard viewers.[46] Digital interventions such as VR may provide engaging, individualized, and incentivized practice opportunities.[39] A survey of healthcare workers' perspectives on simulation-based training in Nigeria showed a lack of awareness of VR

For peer review only

For peer review only - http://bmjopen.bmj.com/site/about/guidelines.xhtml

training, but willingness to utilize VR simulations if they were available.[47] Computer-based simulations have been used in high-resource settings as an adjunct to in-person neonatal and pediatric resuscitation training.[22] VR may support the transfer of knowledge to practice through interactive learning, problem-solving, and standardized feedback.[48 49] Connections that emerge between the participant's offline and in-game identity, and the actual interactions with virtual newborns and mothers within the VR simulation, may modify attitudes and behaviors that relate to clinical practice.[50-53] The healthcare workers in our study responded positively to mobile VR training.

Recently, Erdsal et al. described recommendations to improve the implementation of training programs like HBB by establishing a system for training HCWs and conducting low-dose high frequency (LDHF) practice that is tailored to needs, incentivized, and self-reflective.[54] This practice should emphasize both cognitive and psychomotor skills important for successful neonatal resuscitation. For practicing healthcare providers, the preparation of delivery and initial steps of resuscitation covered by OSCE A checklist are frequently performed in clinical practice, as approximately 10% of all babies born require some resuscitation[22]. A number of digital innovations have been developed, over the past decade, to support *Helping Babies Breathe* education and training.[26 55 56] Based on our findings, after initial training, basic neonatal resuscitation skills may be supported by a range of digital training including VR, video, and even digital guide only. However, prolonged resuscitation (represented by the OSCE B scenario) occurs in only 1% of deliveries, so the performance of these skills is less common in clinical practice, particularly in low-volume healthcare facilities. Simulation practice is important for skills retention in healthcare workers at these facilities and low-dose high frequency practice at a facility-based HBB corner is recommended.[57] Pass rates on bag and mask skills were higher at 6

For peer review only

For peer review only - http://bmjopen.bmj.com/site/about/guidelines.xhtml

months in the VR group. Improvements in BMV performance over time were specifically seen in the critical skills needed to improve ventilation in a baby who is not responding. Resuscitation actions to improve ventilation like *Opening the mouth* of the baby are notably hard to reinforce on manikin-based training because the manikin's mouth is designed to be always open.[58]

This study had several limitations. We only recruited participants from secondary and tertiary healthcare facilities urban and semi-urban resource scarce settings. The study findings may not apply to HCWs who work in high resource settings, at primary healthcare facilities or in rural settings. Although a majority of the study participants reported owning a mobile phone, to ensure uniform access to the study interventions, all participants were provided a study phone to enable access to the digital resources. VR applications are compatible with a wide range of low-cost mobile phones, but not all phones can run VR applications. Participants were asked to access the digital interventions weekly, but the average frequency of access to the application was monthly and may have impacted study findings. The optimal frequency of access is unknown and is an opportunity for future study.

CONCLUSION

Digital interventions supported the retention of neonatal resuscitation knowledge and skills for healthcare workers in Nigeria and Kenya. eHBB VR training was highly feasible and acceptable to healthcare workers in low-middle income countries. eHBB VR may provide additional support for neonatal resuscitation skills retention when compared to other digital interventions.

Acknowledgments: We would like to acknowledge the senior administrative and nursing staff at healthcare facilities that participated in the study. We acknowledge the input of Dr. Susan Niermeyer, Dr. Michael Visik and Dr. David Bolnick in developing the HBB 2nd edition video. We acknowledge the members of the University of Oxford LIFE Project who assisted in

For peer review only - http://bmjopen.bmj.com/site/about/guidelines.xhtml

For peer review only

development of the eHBB VR application: Prof. Michael English, Dr. Niall Winters, Dr. Hilary Edgcombe, Mr. Jakob Rossner, Ms. Naomi Muinga (KEMRI-Wellcome Trust, Kenya) and the staff at National Hospital Abuja who assisted with eHBB VR testing: Dr. Amsa Mairami, Dr. Fatima Mairami, Dr. Adekunle Otuneye, Dr. Lamidi Audu, and Dr. Mariya Mukhtar-Yola. Research assistants were provided with standardized *Helping Babies Breathe* 2nd Edition Facilitator training by Mr. Sammy O. Barasa and Mr. Geoffrey Mwai, of the Kenya *Helping Babies Survive* Master Trainer Corps. We would also like to acknowledge the study team members and collaborators who provided administrative, technical, and other support for the study: Ms. Oyin Akinrinola, Mr. Dillon Afenir, Mr. Bhavani Agnikula Kshatriya, Dr. Benjamin Al-Haddad, Mr. Iman Asangansi, Mr. Prem Avanigadda, Mr. Ariyo Ayokunle, Dr. Brian Bresnahan, Mr. Uchechukwu Chinedu, Mr. Emeka Chukwu, Mr. Chima Chukwudi, Ms. Bailey Clopp, Mr. Domnan Diretnan, Mr. Aniekan Ebito, Ms. Sakina Ginary, Ms. Chioma Ginikanwa, Mr. Oystein Gomo, Mr. Alex McGee, Mr. Manasseh Mmadu, Ms. Ajayi Motunrayo, Mr. Alvin Ogbonna, Mr. Kevin Otieno, Ms. Shruti Patel, Ms. Amanda Stiffler, Dr. Amy Thompson; *in memorium*, Dr. Charles Spiekerman and Ms. Elisabeth Meyers.

Contributorship Statement:
RU and SB conceptualized and designed the study, drafted the initial manuscript, designed the data collection tools, and reviewed and revised the manuscript.
CP and SP designed the data collection tools and reviewed and revised the manuscript.
BE, IF, FM, IA, MN, OO, CA, and AM collected data, and reviewed and revised the manuscript.
JF and DH carried out the initial analyses and reviewed and revised the manuscript.
CE and EE conceptualized and designed the study, collected data, and critically reviewed the manuscript for important intellectual content. All authors participated in the revision of the manuscript, approved the final version and agree to be accountable for all aspects of the work.

Funding/Support: This work was supported by the Bill & Melinda Gates Foundation, grant number OPP1169873.

Competing Interest: Dr. Umoren and Dr. Paton developed the eHBB VR application. Dr. Bucher and Dr. Purkayastha developed the mHBS/DHIS2 application. The other co-authors have no conflicts of interest relevant to this article to disclose.

Ethics approval: The study was approved by the University of Washington Institutional Review Board (IRB) approval number STUDY00005297, the Indiana University IRB approval number 1807371465, the Moi University, Health Research Ethics Committee approval number 0003109 and the University of Lagos College of Medicine Health Research Ethics Committee approval number CMUL/HREC/09/18/445.

Data availability statement: Data are available on request from Dr. Rachel Umoren at nestprog@uw.edu.

Clinical Trial Registration: Not required as the purpose of this study was to examine the effect only on the providers and not patients.

For peer review only

For peer review only - http://bmjopen.bmj.com/site/about/guidelines.xhtml

REFERENCES

1. UNICEF. Neonatal mortality: UNICEF; 2020 [Available from: https://data.unicef.org/topic/child-survival/neonatal-mortality/ accessed September 19 2020.
2. UNCF. United Nations Inter-agency Group for Child Mortality estimation (UN IGME) levels & trends in child mortality: Report 2020, Estimates developed by the United Nations Inter-agency Group for Child Mortality Estimation. New York, 2020.
3. Chou D, Daelmans B, Jolivet RR, et al. Ending preventable maternal and newborn mortality and stillbirths. *BMJ* 2015;351:h4255. doi: 10.1136/bmj.h4255 [published Online First: 2015/09/16]
4. Sharma G, Mathai M, Dickson KE, et al. Quality care during labour and birth: a multi-country analysis of health system bottlenecks and potential solutions. *BMC Pregnancy Childbirth* 2015;15 Suppl 2(Suppl 2):S2. doi: 10.1186/1471-2393-15-S2-S2 [published Online First: 2015/09/24]
5. Enweronu-Laryea C, Dickson KE, Moxon SG, et al. Basic newborn care and neonatal resuscitation: a multi-country analysis of health system bottlenecks and potential solutions. *BMC Pregnancy Childbirth* 2015;15 Suppl 2(Suppl 2):S4. doi: 10.1186/1471-2393-15-S2-S4 [published Online First: 2015/09/24]
6. Lawn JE, Blencowe H, Oza S, et al. Every Newborn: progress, priorities, and potential beyond survival. *Lancet* 2014;384(9938):189-205. doi: 10.1016/S0140-6736(14)60496-7 [published Online First: 2014/05/24]
7. WHO. Standards for improving the quality of care for small and sick newborns in health facilities. 2020
8. Hedstrom A, Perez K, Umoren R, et al. Recent progress in global newborn health: thinking beyond acute to strategic care? *J Perinatol* 2019;39(8):1031-41. doi: 10.1038/s41372-019-0384-z [published Online First: 2019/06/12]
9. Hoban R, Bucher S, Neuman I, et al. 'Helping babies breathe' training in sub-saharan Africa: educational impact and learner impressions. *J Trop Pediatr* 2013;59(3):180-6. doi: 10.1093/tropej/fms077 [published Online First: 2013/01/22]
10. Ashish K, Wrammert J, Clark RB, et al. Reducing perinatal mortality in Nepal using helping babies breathe. *Pediatrics* 2016;137(6)
11. Singhal N, Lockyer J, Fidler H, et al. Helping Babies Breathe: global neonatal resuscitation program development and formative educational evaluation. *Resuscitation* 2012;83(1):90-96. doi: 10.1016/j.resuscitation.2011.07.010 [published Online First: 07/19]
12. Msemo G, Massawe A, Mmbando D, et al. Newborn mortality and fresh stillbirth rates in Tanzania after helping babies breathe training. *Pediatrics* 2013;131(2):e353-60. doi: 10.1542/peds.2012-1795 [published Online First: 2013/01/23]
13. Versantvoort JMD, Kleinhout MY, Ockhuijsen HDL, et al. Helping Babies Breathe and its effects on intrapartum-related stillbirths and neonatal mortality in low-resource settings: a systematic review. *Arch Dis Child* 2020;105(2):127-33. doi: 10.1136/archdischild-2018-316319 [published Online First: 2019/07/07]
14. Eblovi D, Kelly P, Afua G, et al. Retention and use of newborn resuscitation skills following a series of helping babies breathe trainings for midwives in rural Ghana. *Glob Health*

For peer review only - http://bmjopen.bmj.com/site/about/guidelines.xhtml

Action 2017;10(1):1387985. doi: 10.1080/16549716.2017.1387985 [published Online First: 2017/10/24]

15. Musafili A, Essen B, Baribwira C, et al. Evaluating Helping Babies Breathe: training for healthcare workers at hospitals in Rwanda. *Acta Paediatr* 2013;102(1):e34-8. doi: 10.1111/apa.12034 [published Online First: 2012/11/02]
16. Arlington L, Kairuki AK, Isangula KG, et al. Implementation of "Helping Babies Breathe": A 3-year experience in Tanzania. *Pediatrics* 2017;139(5) doi: 10.1542/peds.2016-2132 [published Online First: 2017/05/31]
17. Goudar SS, Somannavar MS, Clark R, et al. Stillbirth and newborn mortality in India after helping babies breathe training. *Pediatrics* 2013;131(2):e344-52. doi: 10.1542/peds.2012-2112 [published Online First: 2013/01/23]
18. Bookman L, Engmann C, Srofenyoh E, et al. Educational impact of a hospital-based neonatal resuscitation program in Ghana. *Resuscitation* 2010;81(9):1180-82.
19. Reisman J, Arlington L, Jensen L, et al. Newborn resuscitation training in resource-limited settings: A systematic literature review. *Pediatrics* 2016;138(2):e20154490. doi: 10.1542/peds.2015-4490 [published Online First: 2016/07/09]
20. Bang A, Patel A, Bellad R, et al. Helping Babies Breathe (HBB) training: What happens to knowledge and skills over time? *BMC Pregnancy Childbirth* 2016;16(1):364.
21. Atukunda IT, Conecker GA. Effect of a low-dose, high-frequency training approach on stillbirths and early neonatal deaths: a before-and-after study in 12 districts of Uganda. *The Lancet Global Health* 2017;5:S12.
22. Sawyer T, Umoren RA, Gray MM. Neonatal resuscitation: advances in training and practice. *Advances in medical education and practice* 2017;8:11-19. doi: 10.2147/AMEP.S109099 [published Online First: 2017/01/18]
23. Weinberg S, Jones D, Worku B, et al. Helping Babies Survive training programs: Evaluating a teaching cascade in Ethiopia. *Ethiop J Health Sci* 2019;29(6):669-76. doi: 10.4314/ejhs.v29i6.3 [published Online First: 2019/11/20]
24. Umoren R, Stadler DJ, Gasior SL, et al. Global collaboration and team-building through 3D virtual environments. *Innovations in Global Medical and Health Education* 2014:1.
25. Sitzmann T. A meta-analytic examination of the instructional effectiveness of computer-based simulation games. *Personnel psychology* 2011;64(2):489-528.
26. Bucher SL, Cardellichio P, Muinga N, et al. Digital health innovations, tools, and resources to support Helping Babies Survive programs. *Pediatrics* 2020;146(Supplement 2):S165-S82.
27. Kamath-Rayne BD, Thukral A, Visick MK, et al. Helping Babies Breathe, Second Edition: A Model for Strengthening Educational Programs to Increase Global Newborn Survival. *Glob Health Sci Pract* 2018;6(3):538-51. doi: 10.9745/GHSP-D-18-00147 [published Online First: 2018/10/06]
28. Umoren R. eHBB VR and eHBB mobile applications on Google play store, 2020.
29. Umoren R. Helping Babies Breathe 2nd edition neonatal resuscitation video 2019 [Available from: https://nestprogram.wistia.com/medias/89c4riojae accessed May 14 2021.
30. Pediatrics AAo. Helping Babies Survive: American Academy of Pediatrics; 2021 [Available from: https://www.aap.org/en-us/advocacy-and-policy/aap-health-initiatives/helping-babies-survive/Pages/Helping-Babies-Breathe-Edition.aspx accessed May 14 2021.

For peer review only

For peer review only - http://bmjopen.bmj.com/site/about/guidelines.xhtml

31. Patterson JK, Girnary S, North K, et al. Innovations in cardiorespiratory monitoring to improve resuscitation with Helping Babies Breathe. *Pediatrics* 2020;146(Supplement 2):S155-S64.
32. Cordova E, Al-Rousan T, Castillo-Angeles M, et al. Effect of low-cost interventions on the retention of knowledge and skills following Helping Babies Breathe training. *Int J Gynaecol Obstet* 2018;142(2):248-54. doi: 10.1002/ijgo.12512 [published Online First: 2018/04/25]
33. Tabangin ME, Josyula S, Taylor KK, et al. Resuscitation skills after Helping Babies Breathe training: a comparison of varying practice frequency and impact on retention of skills in different types of providers. *Int Health* 2018;10(3):163-71. doi: 10.1093/inthealth/ihy017 [published Online First: 2018/04/05]
34. Tsai P-F, Kitch S, Beck C, et al. Using an interactive video simulator to improve certified nursing assistants' dressing assistance and nursing home residents' dressing performance: A pilot study. *Comput Inform Nurs* 2018;36(4):183-92. doi: 10.1097/CIN.0000000000000415
35. Curtis HA, Trang K, Chason KW, et al. Video-Based Learning vs Traditional Lecture for Instructing Emergency Medicine Residents in Disaster Medicine Principles of Mass Triage, Decontamination, and Personal Protective Equipment. *Prehosp Disaster Med* 2018;33(1):7-12. doi: 10.1017/S1049023X1700718X [published Online First: 01/10]
36. Coyne E, Frommolt V, Rands H, et al. Simulation videos presented in a blended learning platform to improve Australian nursing students' knowledge of family assessment. *Nurse Educ Today* 2018;66:96-102. doi: 10.1016/j.nedt.2018.04.012 [published Online First: 04/18]
37. Umoren RA, Gray MM, Schooley N, et al. Effect of video-based telemedicine on transport management of simulated newborns. *Air Med J* 2018;37(5):317-20. doi: 10.1016/j.amj.2018.05.007 [published Online First: 07/27]
38. Sweigart LI, Umoren RA, Scott PJ, et al. Virtual TeamSTEPPS(R) simulations produce teamwork attitude changes among health professions students. *J Nurs Educ* 2016;55(1):31-5. doi: 10.3928/01484834-20151214-08 [published Online First: 2016/01/27]
39. Sheik-Ali S, Edgcombe H, Paton C. Next-Generation virtual and augmented reality in surgical education: a narrative review. *Surg Technol Int* 2019;33
40. Muinga N, Paton C. Virtual reality for medical and nursing training in low-and middle-income countries. 2019
41. Komasawa N, Atagi K, Minami T. Combination of video and manikin hybrid simulation for rapid response emergency airway management training in medical students. *J Clin Anesth* 2018;46:75-76. doi: 10.1016/j.jclinane.2018.02.003 [published Online First: 02/03]
42. Umoren R, Rybas N. Who am I as a healthcare provider? *Integrating an awareness of selfhood and society into virtual learning* 2017:166.
43. Gray MM, Thomas AA, Burns B, et al. Evacuation of Vulnerable and Critical Patients: Multimodal Simulation for Nurse-Led Patient Evacuation. *Simul Healthc* 2020 doi: 10.1097/SIH.0000000000000451 [published Online First: 2020/05/07]
44. Gaps in NRP Provider Performance on Standardized Simulations: A report from the NRP eSim™ Study. Pediatric Academic Societies Conference; 2019; Baltimore, MD.

For peer review only - http://bmjopen.bmj.com/site/about/guidelines.xhtml

45. Jones-Bamman C, Niermeyer S, McConnell K, et al. Teaching Helping Babies Breathe via Telehealth: A New Application in Rural Guatemala. *Biomed Hub* 2019;4(3):1-6. doi: 10.1159/000502934 [published Online First: 2020/01/30]
46. Examining low-cost virtual reality for learning in low-resource environments. Proceedings of the 2017 Conference on Designing Interactive Systems; 2017. ACM.
47. Umoren R, Ezeaka VC, Fajolu IB, et al. Perspectives on simulation-based training from paediatric healthcare providers in Nigeria: a national survey. *BMJ Open* 2020;10(2)
48. Sweigart LI, Umoren RA, Scott PJ, et al. Virtual TeamSTEPPS® Simulations Produce Teamwork Attitude Changes Among Health Professions Students. *Journal of Nursing Education* 2016;55(1):31-35.
49. Joynes C. Distance learning for health: what works-A global review of accredited post-qualification training programmes for health workers in low and middle Income Countries. *London Int Dev Cent* 2011;1
50. Waggoner Z. My avatar, my self: Identity in video role-playing games: McFarland 2009.
51. Parsons TD, Rizzo AA. Affective outcomes of virtual reality exposure therapy for anxiety and specific phobias: A meta-analysis. *Journal of behavior therapy and experimental psychiatry* 2008;39(3):250-61.
52. Seymour NE. VR to OR: a review of the evidence that virtual reality simulation improves operating room performance. *World J Surg* 2008;32(2):182-88.
53. Robb A, White C, Cordar A, et al. A comparison of speaking up behavior during conflict with real and virtual humans. *Comput Hum Behav* 2015;52(C):12-21. doi: 10.1016/j.chb.2015.05.043
54. Ersdal HL, Singhal N, Msemo G, et al. Successful implementation of Helping Babies Survive and Helping Mothers Survive programs—An Utstein formula for newborn and maternal survival. *PLoS One* 2017;12(6):e0178073.
55. Merali HS, Chan NH, Mistry N, et al. Designing and evaluating a novel mobile application for Helping Babies Breathe skills retention in Uganda: comparative study protocol. *BMJ Paediatr Open* 2019;3(1):e000561. doi: 10.1136/bmjpo-2019-000561 [published Online First: 2019/09/25]
56. Somannavar MS, Goudar SS, Revankar AP, et al. Evaluating time between birth to cry or bag and mask ventilation using mobile delivery room timers in India: the NICHD Global Network's Helping Babies Breathe Trial. *BMC Pediatr* 2015;15:93. doi: 10.1186/s12887-015-0408-6 [published Online First: 2015/08/08]
57. Lopreiato JO, Sawyer T. Simulation-based medical education in pediatrics. *Acad Pediatr* 2015;15(2):134-42. doi: 10.1016/j.acap.2014.10.010
58. Sawyer T, Strandjord T, Johnson K, et al. Neonatal airway simulators, how good are they? A comparative study of physical and functional fidelity. *Journal of Perinatology* 2016;36(2):151-56.

Figure 1. Study Diagram

Figure 2. CONSORT Diagram

Figure 3. Pass rates of BMV skills check, OSCE A and OSCE B assessments over time

For peer review only - http://bmjopen.bmj.com/site/about/guidelines.xhtml

635 *Figure 3 Legend:* Pass rates of BMV skills check, OSCE A, and OSCE B assessments over time. Immediate post-course, 1 month, 3 months, and 6 months assessments indicated by solid circles. *Statistically significant changes within each experimental group (VR, video, and control) from the immediate post-course assessment
639 †Significant changes from the 1-month assessment

641 **Figure 4.** OSCE B skills performance on critical steps of ventilation

For peer review only

For peer review only - http://bmjopen.bmj.com/site/about/guidelines.xhtml

Study group	Pre-course period			Follow-up period			
	Baseline assessment	Randomization to study intervention	Pre-course assessment	Immediate post-course	1 month post-course assessment	3 month post-course assessment	6 month post-course assessment
**VR**	Knowledge test	**VR + Digital HBB provider's guide**	Knowledge test, simulation performance	Knowledge test, simulation performance	Knowledge test, simulation performance	Knowledge test, simulation performance	Knowledge test, simulation performance
					**VR + LDHF manikin practice**		
**Video**	Knowledge test	**Video + Digital HBB provider's guide**	Knowledge test, simulation performance	Knowledge test, simulation performance	Knowledge test, simulation performance	Knowledge test, simulation performance	Knowledge test, simulation performance
					**Video + LDHF manikin practice**		
**Standard practice (control)**	Knowledge test	**Digital HBB provider's guide only**	Knowledge test, simulation performance	Knowledge test, simulation performance	Knowledge test, simulation performance	Knowledge test, simulation performance	Knowledge test, simulation performance
					**LDHF manikin practice only**		

VR: Virtual reality
BMV: bag-mask ventilation
LDHF: Low-dose high frequency

Figure 1. Study Diagram

165x100mm (300 x 300 DPI)

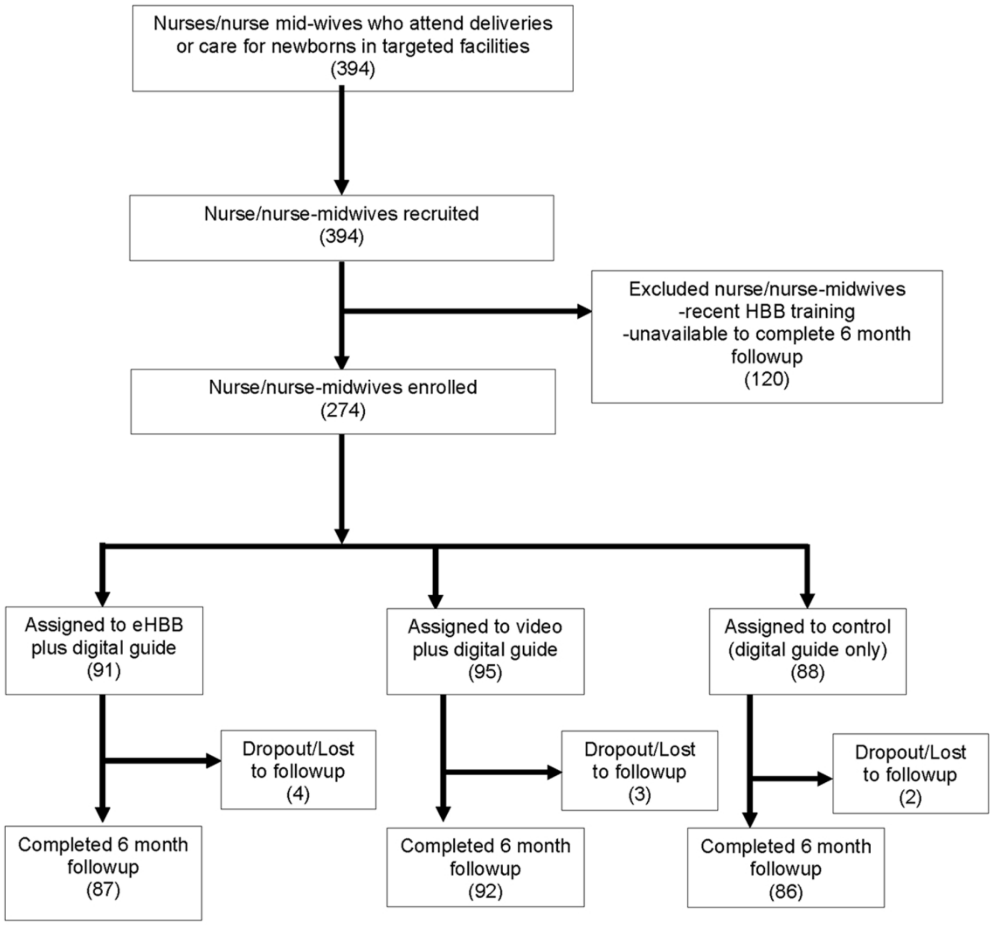

Figure 2. CONSORT Diagram

165x156mm (300 x 300 DPI)

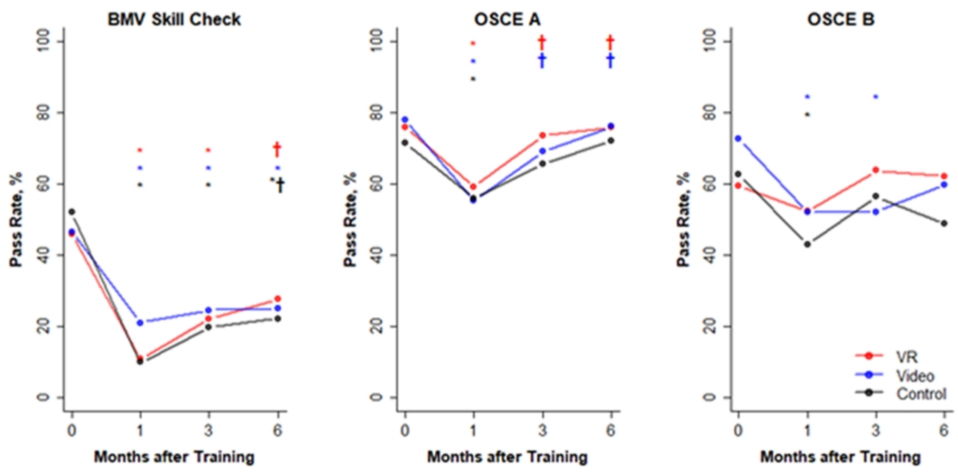

Figure 3. Pass-rates on BMV skills check, OSCE A and OSCE B assessments over time

165x81mm (300 x 300 DPI)

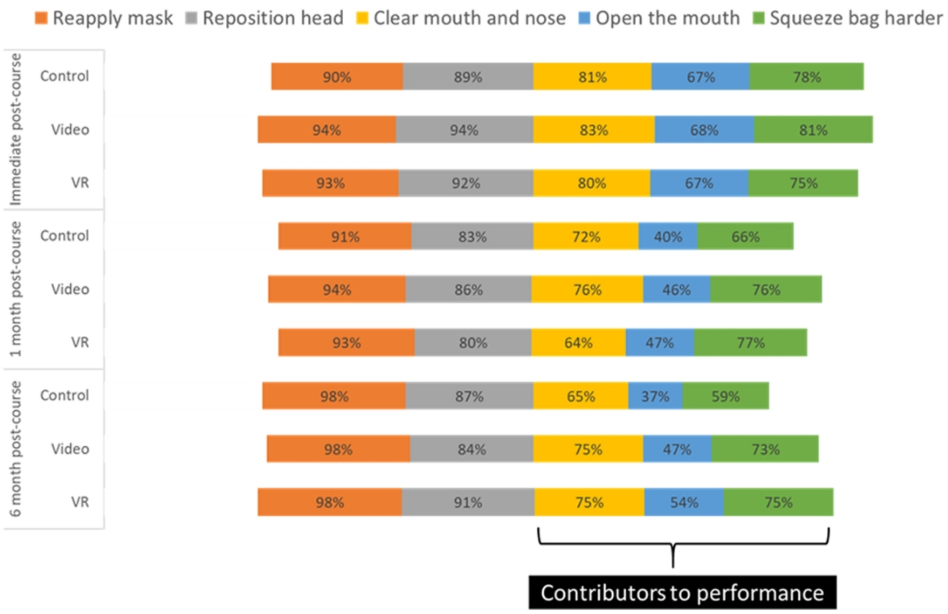

Figure 4. OSCE B skills performance on critical steps of ventilation

150x94mm (300 x 300 DPI)

CONSORT 2010 checklist of information to include when reporting a randomised trial*

Section/Topic	Item No	Checklist item	Reported on page No
**Title and abstract**			
	1a	Identification as a randomised trial in the title	1
	1b	Structured summary of trial design, methods, results, and conclusions (for specific guidance see CONSORT for abstracts)	2
**Introduction**			
Background and objectives	2a	Scientific background and explanation of rationale	3
	2b	Specific objectives or hypotheses	4
**Methods**			
Trial design	3a	Description of trial design (such as parallel, factorial) including allocation ratio	4
	3b	Important changes to methods after trial commencement (such as eligibility criteria), with reasons	N/A
Participants	4a	Eligibility criteria for participants	4
	4b	Settings and locations where the data were collected	4
Interventions	5	The interventions for each group with sufficient details to allow replication, including how and when they were actually administered	5-6
Outcomes	6a	Completely defined pre-specified primary and secondary outcome measures, including how and when they were assessed	5-6
	6b	Any changes to trial outcomes after the trial commenced, with reasons	N/A
Sample size	7a	How sample size was determined	7
	7b	When applicable, explanation of any interim analyses and stopping guidelines	N/A
Randomisation:			
Sequence generation	8a	Method used to generate the random allocation sequence	5
	8b	Type of randomisation; details of any restriction (such as blocking and block size)	5
Allocation concealment mechanism	9	Mechanism used to implement the random allocation sequence (such as sequentially numbered containers), describing any steps taken to conceal the sequence until interventions were assigned	5
Implementation	10	Who generated the random allocation sequence, who enrolled participants, and who assigned participants to interventions	5
Blinding	11a	If done, who was blinded after assignment to interventions (for example, participants, care providers, those	5

For peer review only - http://bmjopen.bmj.com/site/about/guidelines.xhtml

		assessing outcomes) and how	
	11b	If relevant, description of the similarity of interventions	N/A
Statistical methods	12a	Statistical methods used to compare groups for primary and secondary outcomes	7-8
	12b	Methods for additional analyses, such as subgroup analyses and adjusted analyses	7-8
**Results**			
Participant flow (a diagram is strongly recommended)	13a	For each group, the numbers of participants who were randomly assigned, received intended treatment, and were analysed for the primary outcome	8
	13b	For each group, losses and exclusions after randomisation, together with reasons	8
Recruitment	14a	Dates defining the periods of recruitment and follow-up	8
	14b	Why the trial ended or was stopped	8
Baseline data	15	A table showing baseline demographic and clinical characteristics for each group	9
Numbers analysed	16	For each group, number of participants (denominator) included in each analysis and whether the analysis was by original assigned groups	8
Outcomes and estimation	17a	For each primary and secondary outcome, results for each group, and the estimated effect size and its precision (such as 95% confidence interval)	9-14
	17b	For binary outcomes, presentation of both absolute and relative effect sizes is recommended	9-14
Ancillary analyses	18	Results of any other analyses performed, including subgroup analyses and adjusted analyses, distinguishing pre-specified from exploratory	9-14
Harms	19	All important harms or unintended effects in each group (for specific guidance see CONSORT for harms)	9-14
**Discussion**			
Limitations	20	Trial limitations, addressing sources of potential bias, imprecision, and, if relevant, multiplicity of analyses	17
Generalisability	21	Generalisability (external validity, applicability) of the trial findings	17
Interpretation	22	Interpretation consistent with results, balancing benefits and harms, and considering other relevant evidence	15-17
**Other information**			
Registration	23	Registration number and name of trial registry	23
Protocol	24	Where the full trial protocol can be accessed, if available	23
Funding	25	Sources of funding and other support (such as supply of drugs), role of funders	23

*We strongly recommend reading this statement in conjunction with the CONSORT 2010 Explanation and Elaboration for important clarifications on all the items. If relevant, we also recommend reading CONSORT extensions for cluster randomised trials, non-inferiority and equivalence trials, non-pharmacological treatments, herbal interventions, and pragmatic trials. Additional extensions are forthcoming: for those and for up to date references relevant to this checklist, see www.consort-statement.org.

For peer review only - http://bmjopen.bmj.com/site/about/guidelines.xhtml

CONSORT 2010 Flow Diagram

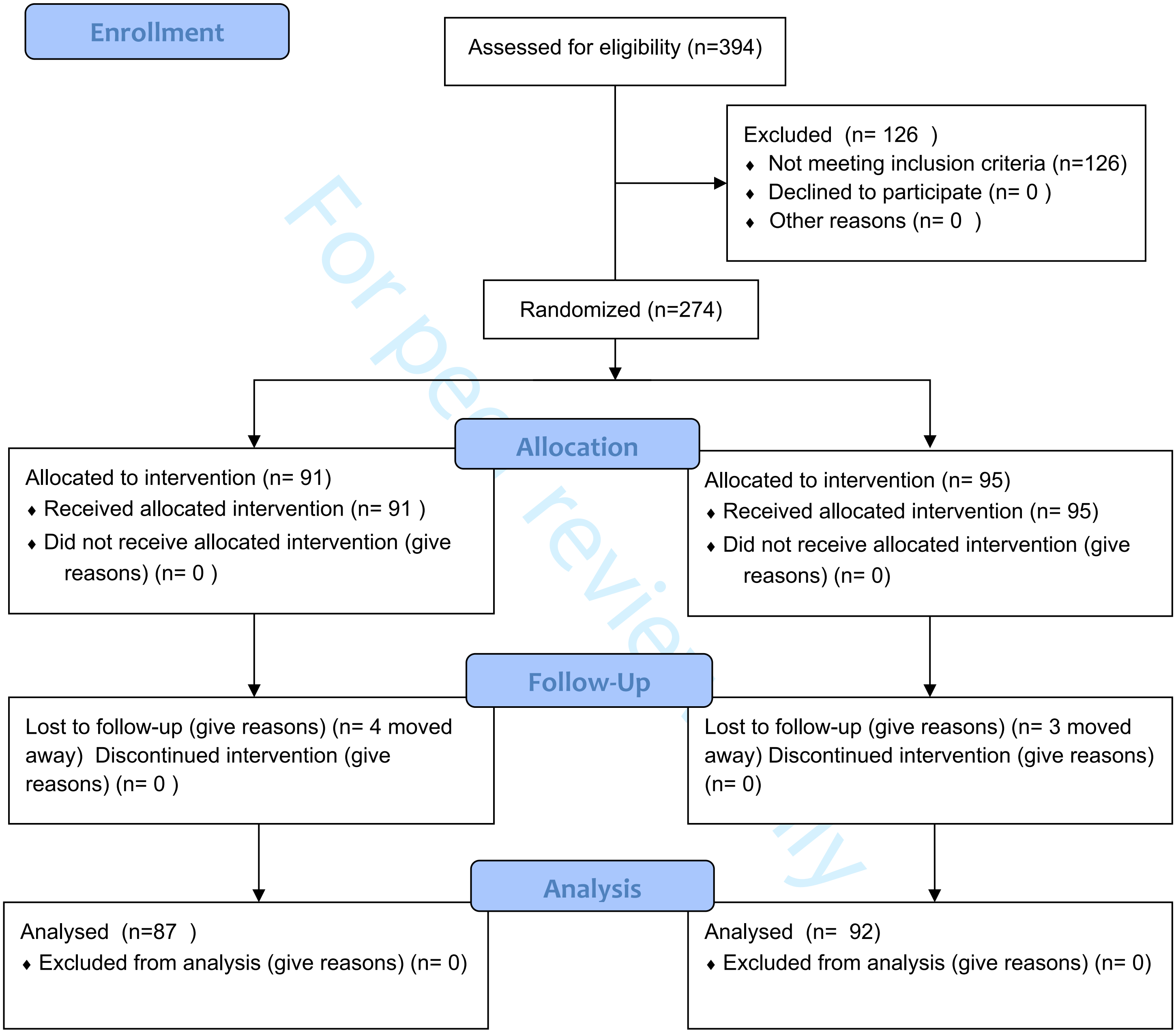

For peer review only

BMJ Open

eHBB: A randomized controlled trial of virtual reality or video for neonatal resuscitation refresher training in healthcare workers in resource scarce settings

Journal:	*BMJ Open*
Manuscript ID	bmjopen-2020-048506.R2
Article Type:	Original research
Date Submitted by the Author:	29-Jul-2021
Complete List of Authors:	Umoren, Rachel; University of Washington<br>Bucher, Sherri; Indiana University School of Medicine<br>Hippe, Daniel; University of Washington<br>Ezenwa, Beatrice; University of Lagos College of Medicine<br>Fajolu, Iretiola; University of Lagos College of Medicine<br>Okwako, Felicitas; Alupe University College<br>Feltner, John; University of Washington<br>Nafula, Mary; Alupe University College<br>Musale, Annet; Alupe University College<br>Olawuyi, Olubukola; University of Lagos College of Medicine<br>Adeboboye, Christianah; University of Lagos College of Medicine<br>Asangansi, Ime; eHealth4everyone<br>Paton, Chris; Nuffield Department of Clinical Medicine, Centre for Tropical Medicine<br>Purkayastha, Saptarshi; Indiana University Purdue University at Indianapolis<br>Ezeaka, Chinyere; University of Lagos College of Medicine<br>Esamai, Fabian; Alupe University College
**Primary Subject Heading**:	Medical education and training
Secondary Subject Heading:	Global health, Nursing, Paediatrics
Keywords:	PAEDIATRICS, Information technology < BIOTECHNOLOGY & BIOINFORMATICS, EDUCATION & TRAINING (see Medical Education & Training), Neonatal intensive & critical care < INTENSIVE & CRITICAL CARE, MEDICAL EDUCATION & TRAINING, NEONATOLOGY

SCHOLARONE™
Manuscripts

I, the Submitting Author has the right to grant and does grant on behalf of all authors of the Work (as defined in the below author licence), an exclusive licence and/or a non-exclusive licence for contributions from authors who are: i) UK Crown employees; ii) where BMJ has agreed a CC-BY licence shall apply, and/or iii) in accordance with the terms applicable for US Federal Government officers or employees acting as part of their official duties; on a worldwide, perpetual, irrevocable, royalty-free basis to BMJ Publishing Group Ltd ("BMJ") its licensees and where the relevant Journal is co-owned by BMJ to the co-owners of the Journal, to publish the Work in this journal and any other BMJ products and to exploit all rights, as set out in our licence.

The Submitting Author accepts and understands that any supply made under these terms is made by BMJ to the Submitting Author unless you are acting as an employee on behalf of your employer or a postgraduate student of an affiliated institution which is paying any applicable article publishing charge ("APC") for Open Access articles. Where the Submitting Author wishes to make the Work available on an Open Access basis (and intends to pay the relevant APC), the terms of reuse of such Open Access shall be governed by a Creative Commons licence – details of these licences and which Creative Commons licence will apply to this Work are set out in our licence referred to above.

Other than as permitted in any relevant BMJ Author's Self Archiving Policies, I confirm this Work has not been accepted for publication elsewhere, is not being considered for publication elsewhere and does not duplicate material already published. I confirm all authors consent to publication of this Work and authorise the granting of this licence.

For peer review only

eHBB: A randomized controlled trial of virtual reality or video for neonatal resuscitation refresher training in healthcare workers in resource scarce settings.

Rachel A. Umoren[*], MB BCh, MS,[a] Sherri Bucher[*], PhD, MA,[b] Daniel S. Hippe MS, [a] Beatrice Ezenwa, MBBS, MSc, MD,[c] Iretiola B. Fajolu, MBBS, [c] Felicitas M. Okwako, MB.ChB, MMED,[d] John Feltner, MS,[a] Mary Nafula, BSN, [d] Annet Musale, [d] Olubukola A. Olawuyi, BNSc, MSc, [c] Christianah O. Adeboboye, BNSc, [c] Ime Asangansi MBBS, PhD,[e] Chris Paton, BMBS, BMedSci, MBA,[f] Saptarshi Purkayastha, PhD,[g] Chinyere Ezeaka[§], MBBS, MPH, [c] Fabian Esamai[§], MB ChB.[d]

[*]*Contributed equally as co-first authors*
[§]*Contributed equally as co-senior authors*

Affiliations:
[a]University of Washington, Seattle, Washington, U.S.A.
[b]Indiana University School of Medicine, Indianapolis, Indiana, U.S.A.
[c]College of Medicine, University of Lagos, Lagos, Nigeria
[d]Alupe University College, Busia, Kenya
[e]eHealth4everyone, Abuja, Nigeria
[f]University of Oxford, Oxford, U.K.
[g]Indiana University – Purdue University, Indianapolis, Indiana, U.S.A

Address correspondence to: Rachel A. Umoren, Department of Pediatrics, Division of Neonatology, 1959 NE Pacific St, Box 356320, Seattle, Washington, 98195, U.S.A. Telephone: 206-543-3200. Email address: rumoren@uw.edu

Text word count: 3639

Keywords: neonatology, nursing, pediatrics, medical education, information technology, virtual reality

For peer review only

ABSTRACT

Objective: To assess the impact of mobile virtual reality (VR) simulations using electronic Helping Babies Breathe (eHBB) or video for the maintenance of neonatal resuscitation skills in healthcare workers in resource scarce settings.

Design: Randomized controlled trial with 6-month follow-up (2018-2020).

Setting: Secondary and tertiary healthcare facilities.

Participants: 274 nurses and midwives assigned to labor and delivery, operating room, and newborn care units were recruited from 20 healthcare facilities in Nigeria and Kenya and randomized to one of three groups: VR (eHBB + digital guide), Video (video + digital guide), or Control (digital guide only) groups before an in-person HBB course.

Intervention(s): eHBB VR simulation or neonatal resuscitation video.

Main outcome(s): Healthcare worker neonatal resuscitation skills using standardized checklists in a simulated setting at 1 month, 3 months and 6 months.

Results: Neonatal resuscitation skills pass-rates were similar among the groups at 6-month follow-up for bag-and-mask ventilation (BMV) skills check (VR 28%, Video 25%, Control 22%, p=0.71), OSCE A (VR 76%, Video 76%, Control 72%, p=0.78) and OSCE B (VR 62%, Video 60%, Control 49%, p=0.18). Relative to the immediate post-course assessments, there was greater retention of BMV skills at 6 months in the VR group (-15% VR, p=0.10; -21% video, p<0.01, -27% control, p=0.001). OSCE B pass-rates in the VR group were numerically higher at 3 months (+4%, p=0.64) and 6 months (+3%, p=0.74) and lower in the Video (-21% at 3 months, p<0.001; -14% at 6 months, p=0.066) and Control groups (-7% at 3 months, p=0.43; -14% at 6 months, p=0.10). On follow-up survey, 95%(n=65) of respondents in the VR group and 98% (n=82) in the Video group would use their assigned intervention again.

Conclusion: eHBB VR training was highly acceptable to healthcare workers in low-middle income countries and may provide additional support for neonatal resuscitation skills retention compared to other digital interventions.

ARTICLE SUMMARY

Strengths and limitations of this study

- This study was a multi-center, randomized controlled trial of mobile virtual reality or video to support neonatal resuscitation skills retention in nurses and midwives who provide neonatal resuscitation in two low-middle income countries.
- The study used an evidence-based Helping Babies Breathe 2nd edition curriculum designed for neonatal resuscitation training in resource-scarce settings.
- Healthcare workers accessed the digital interventions on mobile phones in the 6 months following in-person Helping Babies Breathe training.
- Healthcare workers were recruited from secondary and tertiary healthcare settings in urban and semi-urban resource-scarce settings, so the study findings may not apply to healthcare workers in high resource, primary healthcare, or rural settings.

For peer review only - http://bmjopen.bmj.com/site/about/guidelines.xhtml

INTRODUCTION

In 2019, there were 2.4 million deaths among infants under 28 days of age.[1 2] These neonatal deaths now account for 47% of global under 5-years child mortality,[2] and most cases are preventable.[3-5] The majority of these deaths occur in low-middle income countries (LMICs). Nigeria, with a neonatal mortality rate of 36 deaths per 1000 live births and Kenya at 21 deaths per 1000 live births in 2019,[1] are at significant risk of failing to meet the United Nations Sustainable Development Goal 3 (SDG 3) to reduce neonatal mortality to 12 per 1,000 live births by 2030.[3]

Intrapartum asphyxia or lack of breathing at birth is a leading cause of neonatal mortality.[6 7] Training healthcare workers in neonatal resuscitation using the *Helping Babies Breathe* (HBB) curriculum builds competency and reduces newborn morbidity and mortality.[8-13] However, neonatal resuscitation skills are quickly lost after trainings that utilize the traditional approach of small group facilitated classroom training.[14-19] For this reason, periodic refresher training with "low-dose high frequency" manikin-based simulations are recommended to support neonatal resuscitation skills retention.[20 21] Unfortunately, access to manikin-based simulation is limited by trainer, space, and equipment availability.[22 23] Yet, the high penetration of smartphones and cellular network connectivity in urban and rural areas in LMICs, makes innovative simulation training feasible using mobile virtual reality (VR) simulations for healthcare workers (HCW) who provide care in health facility and community-based settings.

VR simulations are effective educational tools and can be engaged at the learner's convenience, on their own smartphone, with game-based automated feedback that is ideal for episodic learning.[24-26] However little is known of their feasibility, acceptability, or effectiveness for

For peer review only - http://bmjopen.bmj.com/site/about/guidelines.xhtml

neonatal resuscitation skills retention in LMICs. We hypothesized that mobile VR simulation refresher training would address challenges to the quality of newborn resuscitation related to the maintenance of HCW knowledge and skills over time addressing the lack of standardized dissemination of updates to recommended practice and high rates of staff turnover. The objective of this study was to evaluate the impact of eHBB VR used with in-person neonatal resuscitation training on neonatal resuscitation educational indicators and performance outcomes, in comparison to other digital refresher training modalities.

METHODS

Study setting: The study was conducted in Lagos, Nigeria and Busia, Western Kenya. Twelve healthcare facilities (9 secondary and 3 tertiary) were located in Nigeria while 8 facilities were located in Kenya. The healthcare facilities were located in urban and semi-urban areas and all have maternal and newborn services with newborn bed capacity ranging from 2-80 beds and delivery and neonatal unit staffing capacity from 7-124 nurses. The study protocol was approved by the University of Washington Institutional Review Board (IRB), the Indiana University IRB, the Moi University, Health Research Ethics Committee and the University of Lagos College of Medicine Health Research Ethics Committee. See supplementary file 1.

Participants: Study participants consisted of nurses and nurse-midwives assigned to labor and delivery, operating room, and newborn care units. Site coordinators or research assistants requested contact numbers, units, and wards of potential participants from head nurses at identified facilities. Research assistants contacted individuals to determine eligibility and obtained consent. See supplementary file 2.

Inclusion Criteria: Nurses and midwives who participate in deliveries and who provide neonatal resuscitation to inborn or outborn infants and provide study consent.

For peer review only

For peer review only - http://bmjopen.bmj.com/site/about/guidelines.xhtml

Exclusion Criteria: Those who had attended a neonatal resuscitation training course in the 1 year preceding the study; individuals who did not provide neonatal resuscitation as part of their duties or would be unavailable or unwilling to participate in follow-up study activities throughout the 6-month post-initial training period.

Randomization: Study IDs generated for each country site were randomly assigned via a computer-generated algorithm to the VR, Video, and Control groups by a U.S. based study coordinator. Participants were enrolled and assigned a study ID before the HBB course by local study coordinators. Each participant received an Android study phone, pre-loaded with permission-based access linked to their study ID, via the mobile Helping Babies Survive powered by District Health Information System (DHIS2) app (mHBS/DHIS2), to the participant's assigned digital intervention. The data analysis team was blinded to the study assignments.

HBB course structure: The HBB provider course (Second Edition)[27] was taught by study HBB master trainers as one-day, 8-hour long sessions from December 2018-August 2019. A 30-minute orientation was provided on use of the mHBS/DHIS2 app, including how to access the assigned digital intervention. All participants had access to a digitized HBB provider manual through the mHBS/DHIS2 app. The VR group in addition accessed the eHBB VR simulations which consisted of three interactive 3D simulation scenarios representing care of a newborn requiring routine care, some resuscitation and prolonged resuscitation with positive pressure ventilation. The features of eHBB VR have been previously described and the application is available for free download.[26 28] The neonatal resuscitation video used by the video group

For peer review only

For peer review only - http://bmjopen.bmj.com/site/about/guidelines.xhtml

featured preparation for delivery and the resuscitation of a newborn requiring positive pressure ventilation.[29] None of the interventions required internet for use. A total of 274 healthcare workers participated in the in-person Helping Babies Breathe training.

Pre- and post-course assessments: Standardized knowledge and skills assessments were conducted by trained research assistants. The HBB Knowledge Check (15 of 18 multiple-choice questions, ≥80% required to pass) and Bag-and Mask Ventilation Skill Check (BMV; 14 of 14 items required to pass) were conducted pre- and post-course along with the Objective Structured Clinical Examination (OSCE) A checklist on preparation for delivery and initial steps of resuscitation (9 out of 12 items and 3 required items to pass). In addition, the post-course assessment included the OSCE B checklist on prolonged newborn resuscitation (17 out of 23 items and 6 required items to pass). HBB checklists are available for free download from the American Academy of Pediatrics.[30] A demographic survey was completed. See Figure 1.

Figure 1. Study Design

Post-course interventions and follow-up: Following the course, participants were encouraged to use their assigned digital intervention weekly and to engage in standard bag-and-mask skills practice with a manikin at the HBB practice corner set up at their facility. Post-course assessments were repeated at 1, 3, and 6 months after the class. A follow-up survey was completed.

For peer review only - http://bmjopen.bmj.com/site/about/guidelines.xhtml

Data collection: Data were collected in person by study staff who had completed a HBB 2nd edition master trainer course by experienced HBB master trainers. Staff used the mHBS/DHIS2 Tracker app for offline data collection[26]. The mHBS Tracker app contained digitized HBB knowledge check, BMV skill check, and OSCE A and OSCE B checklist and was used by the participants to report their HBB corner practice. The mHBS Trainer app separately tracked educational interventions access and use. To standardize data collection and feedback to study participants, an enhanced neonatal simulator, called *NeoNatalie Live* [Laerdal Medical] was used for bag-and-mask ventilation. Compared to the low-fidelity *NeoNatalie* simulators used for HBB training (including the HBB practice corners in this study), *NeoNatalie Live* manikin can be programmed to simulate key physiological parameters, such as variable rates of lung stiffness and heart rate and provides auditory and visual cues, in the form of “crying” and increased heart rate when the end-user provides bag-and-mask ventilation[31]. In addition, brief automated feedback for the end-user is provided using a Bluetooth-connected tablet device at the end of the assessment as “well done” or “needs improvement” based on bag and mask performance. The use of the *NeoNatalie Live* manikin software enabled the correlation of observer collected metrics with manikin collected data. The automated feedback provided by *Neonatalie Live* was the only feedback provided following each assessment.[31]

Patients or public involvement: Patients or the public were not involved in the design, or conduct, or reporting, or dissemination plans of our research.

Sample size calculations: We hypothesized that there will be at least a 20% difference in the proportion of subjects who pass OSCE B at the 6-month evaluation between the VR group or Video group and the Control group. A sample size of 83 subjects per group would provide 80%

For peer review only

power to detect a difference in pass rates between groups if the true pass rates were 85% and 65%, respectively, based on a two-sided $\alpha = 0.05$. The required total sample size for the three groups (VR, Video, and Control) was 249. We recruited 274 participants total to allow for 10% dropout over the 6-month follow-up period.

Data Analysis: An intention-to-treat analysis was performed, where participants were grouped according to their randomly allocated experimental group (VR, Video, or Control) regardless of their actual exposure. Fisher's exact test was used to test for any differences in pass rates among the three groups for each of the study evaluations: Bag-and-mask ventilation skills assessment (BMV), and standardized simulations of routine care and initial resuscitation (OSCE A) and prolonged resuscitation (OSCE B). Post-hoc pairwise comparisons and comparisons between demographic groups were also performed using Fisher's exact test. Within-group comparisons of evaluation results between timepoints were performed using the Sign test. Participant exposure to the interventions (time in the mHBS Trainer app) and self-reported clinical activity during the follow-up period were compared between experimental groups using the Kruskal-Wallis test and Wilcoxon rank-sum test. All statistical calculations were conducted with the statistical computing language R (version 4.0.0; R Foundation for Statistical Computing, Vienna, Austria). Throughout, two-sided tests were used, with statistical significance defined as $p<0.05$.

RESULTS

Recruitment, training and follow-up were conducted concurrently at participating sites from December 2018 to August 2019 with follow-up continuing until February 2020. Of the 394 nurses and nurse-midwives identified who attended deliveries at the participating sites, 274

For peer review only - http://bmjopen.bmj.com/site/about/guidelines.xhtml

consented to participate in the study. Of the 274 participants, 265 (97%) completed a 6-month assessment, with a similar dropout rate in each group (p=0.52 for the difference between groups, Figure 2).

Figure 2. CONSORT Diagram

Most participants were female (91%), nurse-midwives or midwives (51%), who worked in the labor and delivery ward (72%). Nearly all owned a smartphone (Table 1). Neonatal resuscitation knowledge and skills assessments were conducted immediately after the in-person course. There were no differences in Knowledge check scores [VR 18(17-18), Video 18(17-18), Control 18(17-18), p 0.76] or pass-rates on the BMV [VR 46%(n=83), Video 46%(n=84), Control 52%(n=79), p=0.72], OSCE A [VR 76%(n=91), 78%(n=95), 72%(n=88), p=0.63] and OSCE B [VR 59%(n=91), Video 73%(n=95), Control 62%(n=88), 0.13] assessments across groups on the immediate post-course assessments.

Table 1 Demographics of study participants

**Demographics**	**All**<br>n = 274<br>n (%)	**VR + Digital Guide**<br>n=91<br>n (%)	**Video + Digital Guide**<br>n=95<br>n (%)	**Digital HBB Guide only**<br>n=88<br>n (%)
**Age,** years, mean(SD)	38 (9)	37(9)	41(10)	37 (9)
**Gender**				
Female	250 (91.2)	82 (90.1)	88 (92.6)	80 (90.9)
***Profession**				
Nurse	133 (48.5)	43 (47.3)	46 (48.4)	44 (50.0)
Nurse-midwife	135 (49.3)	48 (52.7)	44 (46.3)	43 (48.9)
Midwife	5 (1.8)	0 (0.0)	5 (5.3)	0 (0.0)

For peer review only

For peer review only - http://bmjopen.bmj.com/site/about/guidelines.xhtml

**Country**				
Kenya	128 (46.7)	44 (48.4)	42 (44.2)	42 (47.7)
Nigeria	146 (53.3)	47 (51.6)	53 (55.8)	46 (52.3)
****Ward**				
Labor/delivery ward	188 (71.8)	62 (71.3)	63 (70.8)	63 (73.3)
Postnatal ward	48 (18.3)	15 (17.2)	15 (16.9)	18 (20.9)
Newborn unit/NICU	17 (6.5)	6 (6.9)	7 (7.9)	4 (4.7)
Operating theatre	9 (3.4)	4 (4.5)	4 (4.5)	1 (1.2)
*****Post-training experience**				
< 5 years	65(23.9)	22(24.4)	17(18.1)	26(29.5)
5-10 years	83(30.5)	34(37.8)	24(25.5)	25(28.4)
11-15 years	40(14.7)	11(12.2)	16(17.0)	13(14.8)
16-20 years	32(11.8)	11(12.2)	13(13.8)	8(9.1)
> 20 years	52(19.1)	12(13.3)	24(25.5)	16(18.2)
**Prior HBB training**	52 (19.0)	16 (17.6)	23 (24.2)	13 (14.8)
****Owns a smartphone**	222 (92.5)	72 (91.2)	75 (91.5)	74 (94.9)

*Legend: *missing value =1; **missing value=12; ***missing value=34; NICU Neonatal Intensive Care Unit*

Neonatal resuscitation knowledge and skills on follow-up assessments

Neonatal resuscitation skills assessments were conducted at 1, 3 and 6 months after the in-person course. Differences in pass-rates on the BMV, OSCE A, and OSCE B checklists across groups on the 6-month post-course assessments were not statistically significant. Table 2.

Table 2. Comparison of 6-month post-course pass rates between groups

	Group n (%)				VR vs. Control			Video vs. Control		
**Variable**	**VR (n=87)**	**Video (n=92)**	**Control (N=86)**	**P-value***	**Δ**	**(95% CI)**	**P-value†**	**Δ**	**(95% CI)**	**P-value†**
BMV skill check	24 (28)	23 (25)	19 (22)	0.71	5.5%	(-8.5, 19.5%)	0.48	2.9%	(-10.7, 16.5%)	0.72

For peer review only - http://bmjopen.bmj.com/site/about/guidelines.xhtml

OSCE A	66 (76)	70 (76)	62 (72)	0.78	3.8%	(-10.5, 18.0%)	0.61	4.0%	(-10.0, 18.0%)	0.61
OSCE B	54 (62)	55 (60)	42 (49)	0.18	13.2%	(-2.6, 29.1%)	0.09	10.9 %	(-4.7, 26.6%)	0.18

Legend: *Fisher's exact test comparing pass rates among the three groups;
†Fisher's exact test comparing pass rates of the VR or video group with the control group.

To determine whether pass-rates were impacted by years of experience, age, profession, ward or prior HBB training, BMV skills, OSCE A and OSCE B pass-rates were compared between groups, one at a time. Participants with < 5 years of experience performed better on the OSCE A and OSCE B immediate post-course assessments (p=0.022 and p=0.034 respectively). Nurse-midwives performed better on BMV skills (p<0.001), OSCE A (p<0.001), and OSCE B immediate post-course assessments (p=0.011) compared to nurses, though pass rates were similar for all three tests at 6 months (p = 0.14 - 0.89). Ward assignment to newborn unit, neonatal intensive care unit (NICU) or postnatal ward was also associated with greater immediate post-course pass-rate on BMV skills (p<0.001), OSCE A (p=0.001) and OSCE B assessments (p<0.001). At the 6-month follow-up assessment, there were no significant differences in BMV skills, OSCE A or OSCE B pass-rates by country site, years of experience, age, ward and HBB training > 1 year prior to the study. Table 3.

Table 3. Pass rates compared between demographic groups.

	BMV Skills Check*		OSCE A*		OSCE B*	
**Demographic**	**Immediate n (%)**	**6-Month n (%)**	**Immediate n (%)**	**6-Month n (%)**	**Immediate n (%)**	**6-Month n (%)**
**Years of Experience**						
<5 years	36 (62.1)	18 (28.6)	57 (87.7)	49 (77.8)	50 (76.9)	37 (58.7)

For peer review only

For peer review only - http://bmjopen.bmj.com/site/about/guidelines.xhtml

5-10 years	27 (41.5)	11 (16.4)	47 (65.3)	52 (77.6)	41 (56.9)	42 (62.7)
11-20 years	20 (44.4)	17 (32.7)	39 (75.0)	38 (73.1)	29 (55.8)	25 (48.1)
20+ years	35 (45.5)	20 (24.7)	62 (74.7)	58 (71.6)	57 (68.7)	46 (56.8)
**p-value†**	0.11	0.18	0.02	0.79	0.03	0.45
**Age**						
20-30 years	39 (60.0)	22 (31.0)	60 (83.3)	54 (76.1)	53 (73.6)	42 (59.2)
31-40 years	36 (45.0)	14 (16.7)	66 (75.9)	59 (70.2)	59 (67.8)	44 (52.4)
41-50 years	29 (42.0)	20 (26.0)	54 (66.7)	61 (79.2)	50 (61.7)	49 (63.6)
51+ years	14 (43.8)	10 (30.3)	26 (76.5)	24 (72.7)	16 (47.1)	16 (48.5)
**p-value†**	0.16	0.16	0.13	0.60	0.05	0.36
Profession						
Nurse	31 (27.7)	34 (26.6)	87 (65.4)	95 (74.2)	76 (57.1)	79 (61.7)
Nurse-midwife	87 (64.9)	32 (23.4)	119 (84.4)	103 (75.2)	102 (72.3)	72 (52.6)
**p-value†**	<0.001	0.57	<0.001	0.89	0.01	0.14
**Ward**						
Labor/delivery ward or operating theatre	77 (42.8)	49 (24.6)	147 (71.7)	150 (75.4)	120 (58.5)	113 (56.8)
NBU/NICU or postnatal ward	39 (70.9)	13 (24.1)	53 (93.0)	37 (68.5)	51 (89.5)	30 (55.6)
**p-value†**	<0.001	>0.99	0.001	0.30	<0.001	0.88
**Prior HBB training**						
Yes	22 (51.2)	14 (28.6)	40 (76.9)	38 (77.6)	29 (55.8)	29 (59.2)
No	96 (47.3)	52 (24.1)	166 (74.8)	160 (74.1)	149 (67.1)	122 (56.5)
**p-value†**	0.74	0.58	0.86	0.72	0.15	0.75

*Values are no (%) if not otherwise specified;
†Fisher's exact test comparing pass rates between groups.
NBU: Newborn Unit; NICU: Neonatal Intensive Care Unit

Neonatal resuscitation performance changes over time

There was a decline in performance on neonatal resuscitation skills assessments at 1 month across all groups with a variable degree of recovery of skills by the 3 and 6-month assessments. Figure 3.

Figure 3. Pass rates of BMV skills check, OSCE A and OSCE B assessments over time

For peer review only

For peer review only - http://bmjopen.bmj.com/site/about/guidelines.xhtml

BMV skill pass rates: BMV skills showed a decline at the 1-month assessment and remained significantly lower than the immediate post-course baseline in all groups at 3 months (-23% VR, p=0.001, -25% Video, p<0.001, -31%, p<0.001) and in the Video and Control groups at 6 months (-15% VR, p=0.10, -21% video, p<0.01, -27% control, p=0.001).

OSCE A pass rates: While pass rates decreased on the OSCE A assessments across all groups at 1-month, the groups improved over time and OSCE A pass-rates were close to the immediate post-course baseline at 6 months (-1% VR, -1% video, 0% control, p=0.83), with the VR group demonstrating an earlier recovery of skills (-2% VR, -9% video, -7% control, p=0.52 at 3 months). At 6 months, the VR group showed good performance on questions: *Prepares the area for ventilation* and *checks function of bag, mask and suction device* [VR 92%(n=87), Video 89%(n=92), Control 84%(n=86), p=0.25), *Recognizes baby is crying and breathing well* [VR 100%(n=87), Video 99%(n=92), Control 95%(n=86), p=0.07] and *Communicates with mother* [VR 94%(n=87), Video 86%(n=92), Control 86%(n=86), p=0.12] although these differences were not statistically significant.

OSCE B pass rates: OSCE B pass-rates were higher than the immediate post-course baseline at 3 and 6 months in the VR group (+4% at 3 months, p=0.64; +3% at 6 months, p=0.74) and lower in the Video (-21% at 3 months, p<0.001; -14% at 6 months, p=0.07) and Control groups (-7% at 3 months p=0.43; -14% at 6 months, p=0.10). Across groups, the performance was sustained on some items of the OSCE B skills checklist that are necessary to improve ventilation such as *Reapply mask* and *Reposition head*, while other recommended steps such as *Clear mouth and nose of secretions*, *Open the mouth and Squeeze bag harder*, showed a greater decline in

For peer review only - http://bmjopen.bmj.com/site/about/guidelines.xhtml

performance. See Figure 4. On post-hoc analysis of OSCE B assessments at the 6-month follow up, there was a statistically significant difference between the VR and Control groups on the frequency of performing the steps: *Opens mouth slightly* (54% vs. 37%, p=0.03) and *Squeezes bag harder* (75% vs. 59%, p=0.04) and providing the target *ventilation rate of 30-50 breaths per minute* (86% VR vs. 73% Control, p=0.04). Differences in performance between the Video and Control group were not statistically significant on these metrics: *Opens mouth slightly* (47% vs. 37%, p=0.23),*Squeezes bag harder* (73% vs. 59%, p=0.06), and *ventilation rate of 30-50 breaths per minute* (85% Video vs. 73% Control, p=0.06).

Figure 4. OSCE B skills performance on critical steps of ventilation

Participant exposure to interventions and clinical activities: Participants were assigned access to study interventions through the mobile Helping Babies Survive (mHBS) app and were encouraged to self-report HBB corner practice and deliveries assisted during the follow up period. The median user time spent in the mHBS Trainer app was 103(85-126) minutes. This software reported metric reflected the time spent accessing the educational interventions in all groups. There was no difference between groups in number of minutes spent using the mHBS trainer 103(85-126) over the 6-month follow up period [VR 101(81-120), Video 108(87-133), Control 102(87-126), p=0.36]. There was no significant difference in self-reported clinical activities with median number of deliveries assisted [VR 25(5-64), Video 25(12-75), Control 28(7-108), p=0.51] and median deliveries requiring bag and mask ventilation [VR 9(3-20), Video 10(4-24), Control 9(4-26), p=0.67]. The median HBB corner practice days were also similar across groups [VR 16(6-42), Video 20(7-38), Control 16(7-51), p=0.86)].

For peer review only

For peer review only - http://bmjopen.bmj.com/site/about/guidelines.xhtml

Participant feedback: Participant feedback indicated overall positive impressions of the VR and video refresher training interventions. On a 6-month follow up survey with a Likert scale of 1-5 with 1 being strongly disagree and 5 being strongly agree, VR group participants agreed/strongly agreed that eHBB VR was easy to use (92%, n=82), realistic (90%, n=81) provided valuable clinical practice (92%, n=81) and feedback (90%, n=68). A majority of the Video group participants also agreed/strongly agreed that the video was easy to use (88%, n=83), realistic (96%, n=81) and valuable for clinical practice (85%, n=81). If given the opportunity, 95% (n=65) of VR and 98% (n=82) of video respondents would use their assigned intervention again.

DISCUSSION

This is the first randomized controlled trial that assesses the impact of mobile VR training for neonatal resuscitation skills retention in healthcare workers in a resource-scarce setting after standard in-person HBB training. Mobile VR training was highly feasible and acceptable to healthcare workers in a low- and middle-income country setting. Previous reports on neonatal resuscitation training using the HBB curriculum have demonstrated a decline in skills within months of training which may interfere with transfer of skills to clinical practice.[14-16 32 33] In this study of digital interventions for neonatal resuscitation skills retention, we found that digital interventions such as VR and video used for refresher training supported the retention of neonatal resuscitation skills in healthcare workers in Nigeria and Kenya. While the 6-month post-course performance was similar across groups, when compared with immediate post-course performance, the decreases in BMV skills pass-rates over time were significant in the video and control groups but not in the VR group. Also, contrary to the expected decline in performance

For peer review only - http://bmjopen.bmj.com/site/about/guidelines.xhtml

over time, OSCE B pass rates were higher at 3 and 6 months than immediately post-course in the VR group, suggesting that the VR training may provide additional support for the skills needed for prolonged neonatal resuscitation.

Both VR and video have been described for training in healthcare workers.[24 34-40] Video is a familiar medium but has been long considered a passive learning modality which should be combined with an active learning modality such as manikin-based practice or simulation.[22 41] Virtual simulation, an active learning modality, is thought to support learning through repetition, user engagement, and identity formation.[42 43] Although the overall performance of the video and VR groups was similar at the 6-month assessment, the VR group demonstrated an increased performance over time on some neonatal resuscitation skills that have been demonstrated gaps in educational simulation-based settings such as the steps to improve bag and mask ventilation.[44] In settings where in-person refresher training is costly and potentially challenging, and may be even more difficult within the context of COVID-19 concerns, digital and telehealth interventions may adequately support the retention of neonatal resuscitation knowledge and skills.[45]

Little is known about the feasibility and acceptability of VR as a novel educational modality for training healthcare workers in resource-scarce settings. Previous descriptions of mobile VR use with school-age children in LMICs suggest that mobile VR simulations can be used to demonstrate real-world phenomena, illustrate abstract concepts, and motivate learners.[46] After using VR, students asked questions that reflected a deeper level of curiosity, engagement, and reflection on lesson topics. They also took ownership of the program by recharging mobile devices and creating their cardboard viewers.[46] Digital interventions such as VR may provide engaging, individualized, and incentivized practice opportunities.[39] A survey of healthcare workers' perspectives on simulation-based training in Nigeria showed a lack of awareness of VR

For peer review only

For peer review only - http://bmjopen.bmj.com/site/about/guidelines.xhtml

training, but willingness to utilize VR simulations if they were available.[47] Computer-based simulations have been used in high-resource settings as an adjunct to in-person neonatal and pediatric resuscitation training.[22] VR may support the transfer of knowledge to practice through interactive learning, problem-solving, and standardized feedback.[48 49] Connections that emerge between the participant's offline and in-game identity, and the actual interactions with virtual newborns and mothers within the VR simulation, may modify attitudes and behaviors that relate to clinical practice.[50-53] The healthcare workers in our study responded positively to mobile VR training.

Recently, Erdsal et al. described recommendations to improve the implementation of training programs like HBB by establishing a system for training HCWs and conducting low-dose high frequency (LDHF) practice that is tailored to needs, incentivized, and self-reflective.[54] This practice should emphasize both cognitive and psychomotor skills important for successful neonatal resuscitation. For practicing healthcare providers, the preparation of delivery and initial steps of resuscitation covered by OSCE A checklist are frequently performed in clinical practice, as approximately 10% of all babies born require some resuscitation[22]. A number of digital innovations have been developed, over the past decade, to support *Helping Babies Breathe* education and training.[26 55 56] Based on our findings, after initial training, basic neonatal resuscitation skills may be supported by a range of digital training including VR, video, and even digital guide only. However, prolonged resuscitation (represented by the OSCE B scenario) occurs in only 1% of deliveries, so the performance of these skills is less common in clinical practice, particularly in low-volume healthcare facilities. Simulation practice is important for skills retention in healthcare workers at these facilities and low-dose high frequency practice at a facility-based HBB corner is recommended.[57] Pass rates on bag and mask skills were higher at 6

For peer review only - http://bmjopen.bmj.com/site/about/guidelines.xhtml

months in the VR group. Improvements in BMV performance over time were specifically seen in the critical skills needed to improve ventilation in a baby who is not responding. Resuscitation actions to improve ventilation like *Opening the mouth* of the baby are notably hard to reinforce on manikin-based training because the manikin's mouth is designed to be always open.[58]

This study had several limitations. We only recruited participants from secondary and tertiary healthcare facilities urban and semi-urban resource scarce settings. The study findings may not apply to HCWs who work in high resource settings, at primary healthcare facilities or in rural settings. Although a majority of the study participants reported owning a mobile phone, to ensure uniform access to the study interventions, all participants were provided a study phone to enable access to the digital resources. VR applications are compatible with a wide range of low-cost mobile phones, but not all phones can run VR applications. Participants were asked to access the digital interventions weekly, but the average frequency of access to the application was monthly and may have impacted study findings. The optimal frequency of access is unknown and is an opportunity for future study.

For peer review only

CONCLUSION

Digital interventions supported the retention of neonatal resuscitation knowledge and skills for healthcare workers in Nigeria and Kenya. eHBB VR training was highly feasible and acceptable to healthcare workers in low-middle income countries. eHBB VR may provide additional support for neonatal resuscitation skills retention when compared to other digital interventions.

Acknowledgments: We would like to acknowledge the senior administrative and nursing staff at healthcare facilities that participated in the study. We acknowledge the input of Dr. Susan Niermeyer, Dr. Michael Visik and Dr. David Bolnick in developing the HBB 2nd edition video. We acknowledge the members of the University of Oxford LIFE Project who assisted in

For peer review only - http://bmjopen.bmj.com/site/about/guidelines.xhtml

development of the eHBB VR application: Prof. Michael English, Dr. Niall Winters, Dr. Hilary Edgcombe, Mr. Jakob Rossner, Ms. Naomi Muinga (KEMRI-Wellcome Trust, Kenya) and the staff at National Hospital Abuja who assisted with eHBB VR testing: Dr. Amsa Mairami, Dr. Fatima Mairami, Dr. Adekunle Otuneye, Dr. Lamidi Audu, and Dr. Mariya Mukhtar-Yola. Research assistants were provided with standardized *Helping Babies Breathe* 2nd Edition Facilitator training by Mr. Sammy O. Barasa and Mr. Geoffrey Mwai, of the Kenya *Helping Babies Survive* Master Trainer Corps. We would also like to acknowledge the study team members and collaborators who provided administrative, technical, and other support for the study: Ms. Oyin Akinrinola, Mr. Dillon Afenir, Mr. Bhavani Agnikula Kshatriya, Dr. Benjamin Al-Haddad, Mr. Iman Asangansi, Mr. Prem Avanigadda, Mr. Ariyo Ayokunle, Dr. Brian Bresnahan, Mr. Uchechukwu Chinedu, Mr. Emeka Chukwu, Mr. Chima Chukwudi, Ms. Bailey Clopp, Mr. Domnan Diretnan, Mr. Aniekan Ebito, Ms. Sakina Ginary, Ms. Chioma Ginikanwa, Mr. Oystein Gomo, Mr. Alex McGee, Mr. Manasseh Mmadu, Ms. Ajayi Motunrayo, Mr. Alvin Ogbonna, Mr. Kevin Otieno, Ms. Shruti Patel, Ms. Amanda Stiffler, Dr. Amy Thompson; *in memorium*, Dr. Charles Spiekerman and Ms. Elisabeth Meyers.

Contributorship Statement:
RU and SB conceptualized and designed the study, drafted the initial manuscript, designed the data collection tools, and reviewed and revised the manuscript.
CP and SP designed the data collection tools and reviewed and revised the manuscript.
BE, IF, FM, IA, MN, OO, CA, and AM collected data, and reviewed and revised the manuscript.
JF and DH carried out the initial analyses and reviewed and revised the manuscript.
CE and FE conceptualized and designed the study, collected data, and critically reviewed the manuscript for important intellectual content. All authors participated in the revision of the manuscript, approved the final version and agree to be accountable for all aspects of the work.

Funding/Support: This work was supported by the Bill & Melinda Gates Foundation, grant number OPP1169873.

Competing Interest: Dr. Umoren and Dr. Paton developed the eHBB VR application. Dr. Bucher and Dr. Purkayastha developed the mHBS/DHIS2 application. The other co-authors have no conflicts of interest relevant to this article to disclose.

Ethics approval: The study was approved by the University of Washington Institutional Review Board (IRB) approval number STUDY00005297, the Indiana University IRB approval number 1807371465, the Moi University, Health Research Ethics Committee approval number 0003109 and the University of Lagos College of Medicine Health Research Ethics Committee approval number CMUL/HREC/09/18/445.

Data availability statement: Data are available on request from Dr. Rachel Umoren at nestprog@uw.edu.

Clinical Trial Registration: Not required as the purpose of this study was to examine the effect only on the providers and not patients.

For peer review only - http://bmjopen.bmj.com/site/about/guidelines.xhtml

REFERENCES

1. UNICEF. Neonatal mortality: UNICEF; 2020 [Available from: https://data.unicef.org/topic/child-survival/neonatal-mortality/ accessed September 19 2020.
2. UNCF. United Nations Inter-agency Group for Child Mortality estimation (UN IGME) levels & trends in child mortality: Report 2020, Estimates developed by the United Nations Inter-agency Group for Child Mortality Estimation. New York, 2020.
3. Chou D, Daelmans B, Jolivet RR, et al. Ending preventable maternal and newborn mortality and stillbirths. *BMJ* 2015;351:h4255. doi: 10.1136/bmj.h4255 [published Online First: 2015/09/16]
4. Sharma G, Mathai M, Dickson KE, et al. Quality care during labour and birth: a multi-country analysis of health system bottlenecks and potential solutions. *BMC Pregnancy Childbirth* 2015;15 Suppl 2(Suppl 2):S2. doi: 10.1186/1471-2393-15-S2-S2 [published Online First: 2015/09/24]
5. Enweronu-Laryea C, Dickson KE, Moxon SG, et al. Basic newborn care and neonatal resuscitation: a multi-country analysis of health system bottlenecks and potential solutions. *BMC Pregnancy Childbirth* 2015;15 Suppl 2(Suppl 2):S4. doi: 10.1186/1471-2393-15-S2-S4 [published Online First: 2015/09/24]
6. Lawn JE, Blencowe H, Oza S, et al. Every Newborn: progress, priorities, and potential beyond survival. *Lancet* 2014;384(9938):189-205. doi: 10.1016/S0140-6736(14)60496-7 [published Online First: 2014/05/24]
7. WHO. Standards for improving the quality of care for small and sick newborns in health facilities. 2020
8. Hedstrom A, Perez K, Umoren R, et al. Recent progress in global newborn health: thinking beyond acute to strategic care? *J Perinatol* 2019;39(8):1031-41. doi: 10.1038/s41372-019-0384-z [published Online First: 2019/06/12]
9. Hoban R, Bucher S, Neuman I, et al. 'Helping babies breathe' training in sub-saharan Africa: educational impact and learner impressions. *J Trop Pediatr* 2013;59(3):180-6. doi: 10.1093/tropej/fms077 [published Online First: 2013/01/22]
10. Ashish K, Wrammert J, Clark RB, et al. Reducing perinatal mortality in Nepal using helping babies breathe. *Pediatrics* 2016;137(6)
11. Singhal N, Lockyer J, Fidler H, et al. Helping Babies Breathe: global neonatal resuscitation program development and formative educational evaluation. *Resuscitation* 2012;83(1):90-96. doi: 10.1016/j.resuscitation.2011.07.010 [published Online First: 07/19]
12. Msemo G, Massawe A, Mmbando D, et al. Newborn mortality and fresh stillbirth rates in Tanzania after helping babies breathe training. *Pediatrics* 2013;131(2):e353-60. doi: 10.1542/peds.2012-1795 [published Online First: 2013/01/23]
13. Versantvoort JMD, Kleinhout MY, Ockhuijsen HDL, et al. Helping Babies Breathe and its effects on intrapartum-related stillbirths and neonatal mortality in low-resource settings: a systematic review. *Arch Dis Child* 2020;105(2):127-33. doi: 10.1136/archdischild-2018-316319 [published Online First: 2019/07/07]
14. Eblovi D, Kelly P, Afua G, et al. Retention and use of newborn resuscitation skills following a series of helping babies breathe trainings for midwives in rural Ghana. *Glob Health*

For peer review only - http://bmjopen.bmj.com/site/about/guidelines.xhtml

Action 2017;10(1):1387985. doi: 10.1080/16549716.2017.1387985 [published Online First: 2017/10/24]
15. Musafili A, Essen B, Baribwira C, et al. Evaluating Helping Babies Breathe: training for healthcare workers at hospitals in Rwanda. *Acta Paediatr* 2013;102(1):e34-8. doi: 10.1111/apa.12034 [published Online First: 2012/11/02]
16. Arlington L, Kairuki AK, Isangula KG, et al. Implementation of "Helping Babies Breathe": A 3-year experience in Tanzania. *Pediatrics* 2017;139(5) doi: 10.1542/peds.2016-2132 [published Online First: 2017/05/31]
17. Goudar SS, Somannavar MS, Clark R, et al. Stillbirth and newborn mortality in India after helping babies breathe training. *Pediatrics* 2013;131(2):e344-52. doi: 10.1542/peds.2012-2112 [published Online First: 2013/01/23]
18. Bookman L, Engmann C, Srofenyoh E, et al. Educational impact of a hospital-based neonatal resuscitation program in Ghana. *Resuscitation* 2010;81(9):1180-82.
19. Reisman J, Arlington L, Jensen L, et al. Newborn resuscitation training in resource-limited settings: A systematic literature review. *Pediatrics* 2016;138(2):e20154490. doi: 10.1542/peds.2015-4490 [published Online First: 2016/07/09]
20. Bang A, Patel A, Bellad R, et al. Helping Babies Breathe (HBB) training: What happens to knowledge and skills over time? *BMC Pregnancy Childbirth* 2016;16(1):364.
21. Atukunda IT, Conecker GA. Effect of a low-dose, high-frequency training approach on stillbirths and early neonatal deaths: a before-and-after study in 12 districts of Uganda. *The Lancet Global Health* 2017;5:S12.
22. Sawyer T, Umoren RA, Gray MM. Neonatal resuscitation: advances in training and practice. *Advances in medical education and practice* 2017;8:11-19. doi: 10.2147/AMEP.S109099 [published Online First: 2017/01/18]
23. Weinberg S, Jones D, Worku B, et al. Helping Babies Survive training programs: Evaluating a teaching cascade in Ethiopia. *Ethiop J Health Sci* 2019;29(6):669-76. doi: 10.4314/ejhs.v29i6.3 [published Online First: 2019/11/20]
24. Umoren R, Stadler DJ, Gasior SL, et al. Global collaboration and team-building through 3D virtual environments. *Innovations in Global Medical and Health Education* 2014:1.
25. Sitzmann T. A meta-analytic examination of the instructional effectiveness of computer-based simulation games. *Personnel psychology* 2011;64(2):489-528.
26. Bucher SL, Cardellichio P, Muinga N, et al. Digital health innovations, tools, and resources to support Helping Babies Survive programs. *Pediatrics* 2020;146(Supplement 2):S165-S82.
27. Kamath-Rayne BD, Thukral A, Visick MK, et al. Helping Babies Breathe, Second Edition: A Model for Strengthening Educational Programs to Increase Global Newborn Survival. *Glob Health Sci Pract* 2018;6(3):538-51. doi: 10.9745/GHSP-D-18-00147 [published Online First: 2018/10/06]
28. Umoren R. eHBB VR and eHBB mobile applications on Google play store, 2020.
29. Umoren R. Helping Babies Breathe 2nd edition neonatal resuscitation video 2019 [Available from: https://nestprogram.wistia.com/medias/89c4riojae accessed May 14 2021.
30. Pediatrics AAo. Helping Babies Survive: American Academy of Pediatrics; 2021 [Available from: https://www.aap.org/en-us/advocacy-and-policy/aap-health-initiatives/helping-babies-survive/Pages/Helping-Babies-Breathe-Edition.aspx accessed May 14 2021.

For peer review only - http://bmjopen.bmj.com/site/about/guidelines.xhtml

31. Patterson JK, Girnary S, North K, et al. Innovations in cardiorespiratory monitoring to improve resuscitation with Helping Babies Breathe. *Pediatrics* 2020;146(Supplement 2):S155-S64.
32. Cordova E, Al-Rousan T, Castillo-Angeles M, et al. Effect of low-cost interventions on the retention of knowledge and skills following Helping Babies Breathe training. *Int J Gynaecol Obstet* 2018;142(2):248-54. doi: 10.1002/ijgo.12512 [published Online First: 2018/04/25]
33. Tabangin ME, Josyula S, Taylor KK, et al. Resuscitation skills after Helping Babies Breathe training: a comparison of varying practice frequency and impact on retention of skills in different types of providers. *Int Health* 2018;10(3):163-71. doi: 10.1093/inthealth/ihy017 [published Online First: 2018/04/05]
34. Tsai P-F, Kitch S, Beck C, et al. Using an interactive video simulator to improve certified nursing assistants' dressing assistance and nursing home residents' dressing performance: A pilot study. *Comput Inform Nurs* 2018;36(4):183-92. doi: 10.1097/CIN.0000000000000415
35. Curtis HA, Trang K, Chason KW, et al. Video-Based Learning vs Traditional Lecture for Instructing Emergency Medicine Residents in Disaster Medicine Principles of Mass Triage, Decontamination, and Personal Protective Equipment. *Prehosp Disaster Med* 2018;33(1):7-12. doi: 10.1017/S1049023X1700718X [published Online First: 01/10]
36. Coyne E, Frommolt V, Rands H, et al. Simulation videos presented in a blended learning platform to improve Australian nursing students' knowledge of family assessment. *Nurse Educ Today* 2018;66:96-102. doi: 10.1016/j.nedt.2018.04.012 [published Online First: 04/18]
37. Umoren RA, Gray MM, Schooley N, et al. Effect of video-based telemedicine on transport management of simulated newborns. *Air Med J* 2018;37(5):317-20. doi: 10.1016/j.amj.2018.05.007 [published Online First: 07/27]
38. Sweigart LI, Umoren RA, Scott PJ, et al. Virtual TeamSTEPPS(R) simulations produce teamwork attitude changes among health professions students. *J Nurs Educ* 2016;55(1):31-5. doi: 10.3928/01484834-20151214-08 [published Online First: 2016/01/27]
39. Sheik-Ali S, Edgcombe H, Paton C. Next-Generation virtual and augmented reality in surgical education: a narrative review. *Surg Technol Int* 2019;33
40. Muinga N, Paton C. Virtual reality for medical and nursing training in low-and middle-income countries. 2019
41. Komasawa N, Atagi K, Minami T. Combination of video and manikin hybrid simulation for rapid response emergency airway management training in medical students. *J Clin Anesth* 2018;46:75-76. doi: 10.1016/j.jclinane.2018.02.003 [published Online First: 02/03]
42. Umoren R, Rybas N. Who am I as a healthcare provider? *Integrating an awareness of selfhood and society into virtual learning* 2017:166.
43. Gray MM, Thomas AA, Burns B, et al. Evacuation of Vulnerable and Critical Patients: Multimodal Simulation for Nurse-Led Patient Evacuation. *Simul Healthc* 2020 doi: 10.1097/SIH.0000000000000451 [published Online First: 2020/05/07]
44. Gaps in NRP Provider Performance on Standardized Simulations: A report from the NRP eSim™ Study. Pediatric Academic Societies Conference; 2019; Baltimore, MD.

For peer review only - http://bmjopen.bmj.com/site/about/guidelines.xhtml

45. Jones-Bamman C, Niermeyer S, McConnell K, et al. Teaching Helping Babies Breathe via Telehealth: A New Application in Rural Guatemala. *Biomed Hub* 2019;4(3):1-6. doi: 10.1159/000502934 [published Online First: 2020/01/30]

46. Examining low-cost virtual reality for learning in low-resource environments. Proceedings of the 2017 Conference on Designing Interactive Systems; 2017. ACM.

47. Umoren R, Ezeaka VC, Fajolu IB, et al. Perspectives on simulation-based training from paediatric healthcare providers in Nigeria: a national survey. *BMJ Open* 2020;10(2)

48. Sweigart LI, Umoren RA, Scott PJ, et al. Virtual TeamSTEPPS® Simulations Produce Teamwork Attitude Changes Among Health Professions Students. *Journal of Nursing Education* 2016;55(1):31-35.

49. Joynes C. Distance learning for health: what works-A global review of accredited post-qualification training programmes for health workers in low and middle Income Countries. *London Int Dev Cent* 2011;1

50. Waggoner Z. My avatar, my self: Identity in video role-playing games: McFarland 2009.

51. Parsons TD, Rizzo AA. Affective outcomes of virtual reality exposure therapy for anxiety and specific phobias: A meta-analysis. *Journal of behavior therapy and experimental psychiatry* 2008;39(3):250-61.

52. Seymour NE. VR to OR: a review of the evidence that virtual reality simulation improves operating room performance. *World J Surg* 2008;32(2):182-88.

53. Robb A, White C, Cordar A, et al. A comparison of speaking up behavior during conflict with real and virtual humans. *Comput Hum Behav* 2015;52(C):12-21. doi: 10.1016/j.chb.2015.05.043

54. Ersdal HL, Singhal N, Msemo G, et al. Successful implementation of Helping Babies Survive and Helping Mothers Survive programs—An Utstein formula for newborn and maternal survival. *PLoS One* 2017;12(6):e0178073.

55. Merali HS, Chan NH, Mistry N, et al. Designing and evaluating a novel mobile application for Helping Babies Breathe skills retention in Uganda: comparative study protocol. *BMJ Paediatr Open* 2019;3(1):e000561. doi: 10.1136/bmjpo-2019-000561 [published Online First: 2019/09/25]

56. Somannavar MS, Goudar SS, Revankar AP, et al. Evaluating time between birth to cry or bag and mask ventilation using mobile delivery room timers in India: the NICHD Global Network's Helping Babies Breathe Trial. *BMC Pediatr* 2015;15:93. doi: 10.1186/s12887-015-0408-6 [published Online First: 2015/08/08]

57. Lopreiato JO, Sawyer T. Simulation-based medical education in pediatrics. *Acad Pediatr* 2015;15(2):134-42. doi: 10.1016/j.acap.2014.10.010

58. Sawyer T, Strandjord T, Johnson K, et al. Neonatal airway simulators, how good are they? A comparative study of physical and functional fidelity. *Journal of Perinatology* 2016;36(2):151-56.

Figure 1. Study Diagram

Figure 2. CONSORT Diagram

Figure 3. Pass rates of BMV skills check, OSCE A and OSCE B assessments over time

For peer review only - http://bmjopen.bmj.com/site/about/guidelines.xhtml

635 *Figure 3 Legend:* Pass rates of BMV skills check, OSCE A, and OSCE B assessments over time. Immediate post-course, 1 month, 3 months, and 6 months assessments indicated by solid circles. *Statistically significant changes within each experimental group (VR, video, and control) from the immediate post-course assessment
†Significant changes from the 1-month assessment

Figure 4. OSCE B skills performance on critical steps of ventilation

For peer review only

For peer review only - http://bmjopen.bmj.com/site/about/guidelines.xhtml

Study group	Pre-course period			Follow-up period			
	Baseline assessment	Randomization to study intervention	Pre-course assessment	Immediate post-course	1 month post-course assessment	3 month post-course assessment	6 month post-course assessment
**VR**	Knowledge test	**VR + Digital HBB provider's guide**	Knowledge test, simulation performance	Knowledge test, simulation performance	Knowledge test, simulation performance	Knowledge test, simulation performance	Knowledge test, simulation performance
					**VR + LDHF manikin practice**		
**Video**	Knowledge test	**Video + Digital HBB provider's guide**	Knowledge test, simulation performance	Knowledge test, simulation performance	Knowledge test, simulation performance	Knowledge test, simulation performance	Knowledge test, simulation performance
					**Video + LDHF manikin practice**		
**Standard practice (control)**	Knowledge test	**Digital HBB provider's guide only**	Knowledge test, simulation performance	Knowledge test, simulation performance	Knowledge test, simulation performance	Knowledge test, simulation performance	Knowledge test, simulation performance
					**LDHF manikin practice only**		

VR: Virtual reality
BMV: bag-mask ventilation
LDHF: Low-dose high frequency

Figure 1. Study Diagram

165x100mm (300 x 300 DPI)

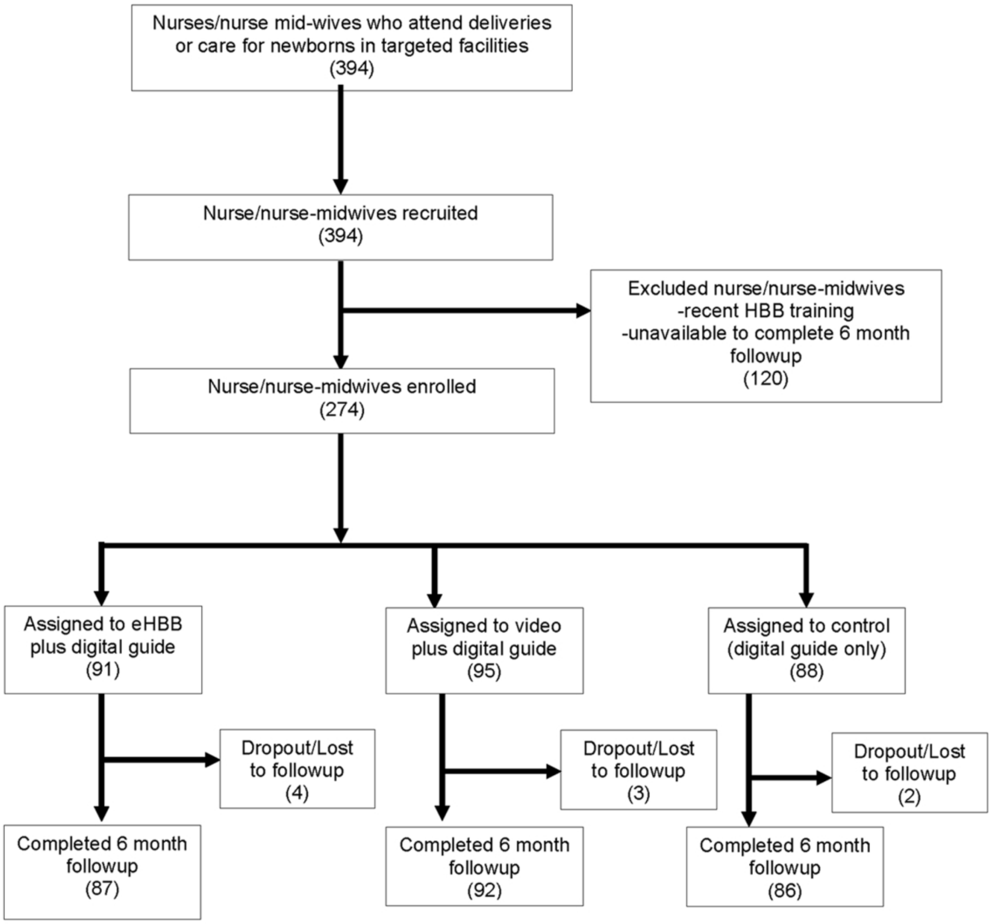

Figure 2. CONSORT Diagram

165x156mm (300 x 300 DPI)

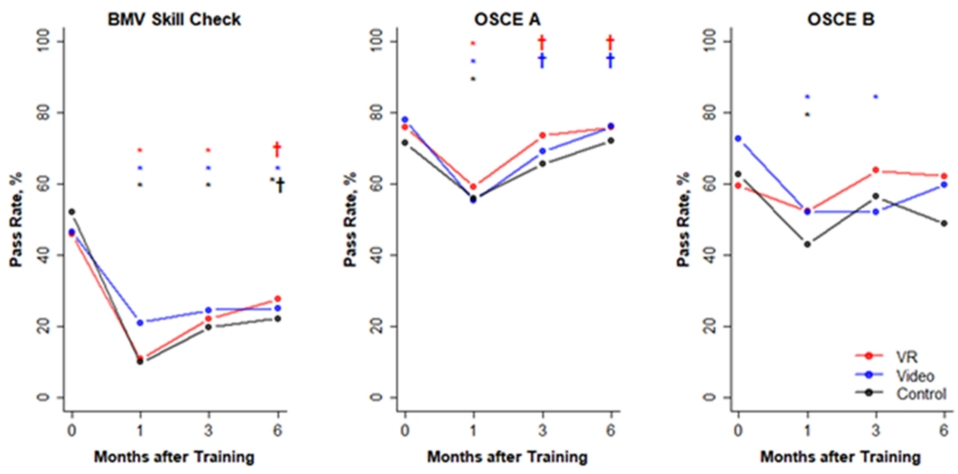

Figure 3. Pass-rates on BMV skills check, OSCE A and OSCE B assessments over time

165x81mm (300 x 300 DPI)

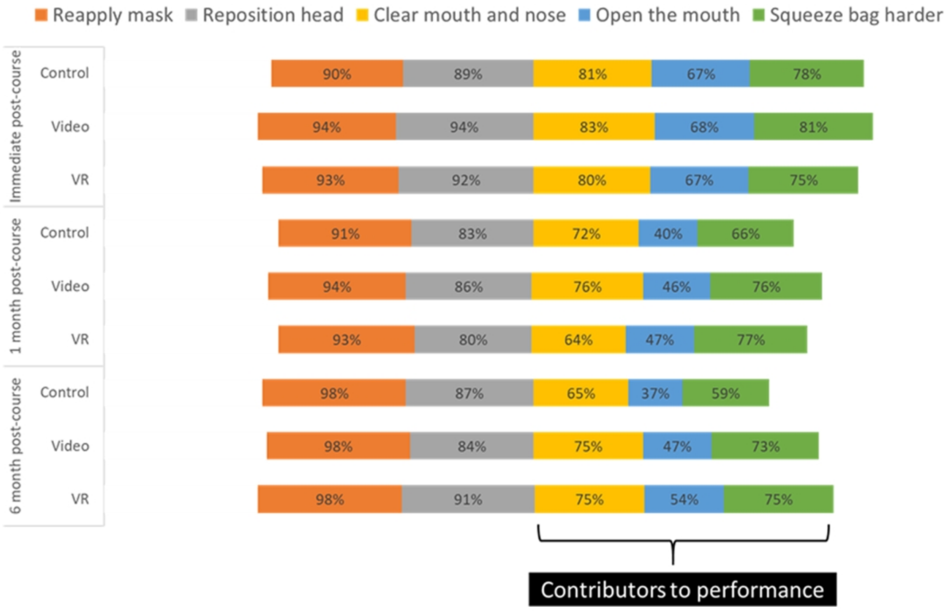

Figure 4. OSCE B skills performance on critical steps of ventilation

150x94mm (300 x 300 DPI)

SUPPLEMENTARY FILE 1

STUDY PROTOCOL

eHBB (Helping Babies Breathe)

+

mHBS (mobile Helping Babies Survive)/DHIS2:

Virtual reality technology and DHIS2 mobile data collection to improve newborn healthcare delivery in low and middle income countries

For peer review only

Study Investigators

Principal Investigators

Dr. Rachel A. Umoren
Associate Professor of Paediatrics, University of Washington School of Medicine, Seattle, Washington, U.S.A.

Dr. Sherri Bucher
Associate Research Professor of Paediatrics, Indiana University School of Medicine, Indianapolis, Indiana, U.S.A.

Site PI (Kenya)
Prof. Fabian Esamai
Professor of Paediatrics, Moi University; Principal, Alupe University College, Kenya

Site PI (Nigeria)
Prof. Chinyere V. Ezeaka
Professor of Paediatrics, College of Medicine, University of Lagos. Head of the Perinatology-Neonatology Division, Lagos University Teaching Hospital. Idi-araba, Lagos, Nigeria.

Co-Investigators

Dr. Ime Asangansi
eHealth4everyone, Abuja, Nigeria
Dr. Brian Bresnahan
University of Washington, Seattle, Washington, U.S.A.
Mr. Daniel Hippe
University of Washington, Seattle, Washington, U.S.A.
Dr. Chris Paton
LIFE Project, Oxford University, Oxford, U.K.
Dr. Saptarshi Purkayastha
Indiana University, Indianapolis, Indiana, U.S.A.

Acknowledgments:

We would like to acknowledge the senior administrative and nursing staff at healthcare facilities that participated in the study. We acknowledge the input of Dr. Susan Niermeyer, Dr. Michael Visik and Dr. David Bolnick in developing the HBB 2nd edition video. We acknowledge the members of the University of Oxford LIFE Project who assisted in development of the eHBB VR application: Prof. Michael English, Dr. Niall Winters, Dr. Hilary Edgcombe, Mr. Jakob Rossner, Ms. Naomi Muinga (KEMRI-Wellcome Trust, Kenya) and the staff at National Hospital Abuja who assisted with eHBB VR testing: Dr. Amsa Mairami, Dr. Fatima Mairami, Dr. Adekunle Otuneye, Dr. Lamidi Audu, and Dr. Mariya Mukhtar-Yola. Research assistants were provided with standardized Helping Babies Breathe 2nd Edition Facilitator training by Mr. Sammy O. Barasa and Mr. Geoffrey Mwai, of the Kenya Helping Babies Survive Master Trainer Corps. We would also like to acknowledge the study team members and collaborators who provided administrative, technical, and other support for the study: Mr. Alex McGee, Dr. Iretiola Fajolu, Dr. Beatrice Ezenwa, Dr. Felicitas Okwako, Mr. John Feltner, Ms. Mary Nafula, Ms. Annet Musale, Ms. Olubukola Olawuyi, Ms. Christianah Adeboboye, Ms. Oyin Akinrinola, Mr. Dillon Afenir, Mr. Bhavani Agnikula Kshatriya, Dr. Benjamin Al-Haddad, Mr. Iman Asangansi, Mr. Prem Avanigadda, Mr. Ariyo Ayokunle, Mr. Uchechukwu Chinedu, Mr. Emeka Chukwu, Mr. Chima Chukwudi, Ms. Bailey Clopp, Mr. Domnan Diretnan, Mr. Aniekan Ebito, Ms. Sakina Ginary, Ms. Chioma Ginikanwa, Mr. Oystein Gomo, Mr. Manasseh Mmadu, Ms. Ajayi Motunrayo, Mr. Alvin Ogbonna, Mr. Kevin Otieno, Ms. Shruti Patel, Ms. Amanda Stiffler, Dr. Amy Thompson; *in memorium,* Dr. Charles Spiekerman and Ms. Elisabeth Meyers.

For peer review only - http://bmjopen.bmj.com/site/about/guidelines.xhtml

Table of Contents

1. Study Overview 5
2. Background 6
3. Study Objective 7
4. Study Procedures 7
4.1 Study Participants 7
4.2 Study Facilities 7
4.3 Inclusion Criteria 7
4.4 Exclusion Criteria 8
4.5 Recruitment 8
4.6 Informed Consent 8
4.7 Randomization/allocation procedure 8
4.8 Outcome Measures 8
5. Study Interventions 9
5.1 Pre-course interventions 9
5.2 Pre-course assessments 10
5.3 Helping Babies Breathe 2nd Edition Course 10
5.4 Immediate post-course assessments 12
5.5 Follow up assessments 12
6. Study Incentives 12
7. Data management 13
8. Data analysis 13
8.1 Sample size calculations 13
9. Qualitative studies 14
10. References 15
APPENDICES 17
Appendix 1. Abbreviations/Definition of Terms 17
Appendix 2. Study Diagram 18
Appendix 3. Recruitment Information 19
Appendix 4. Consent Form 20
Appendix 5. Participant Surveys 24
Appendix 6. HBB Knowledge Check 31

For peer review only - http://bmjopen.bmj.com/site/about/guidelines.xhtml

Appendix 7. Simulation Checklists 34

For peer review only

For peer review only - http://bmjopen.bmj.com/site/about/guidelines.xhtml

eHBB + mHBS/DHIS2: Virtual reality technology and DHIS2 mobile data collection to improve newborn healthcare delivery in low and middle income countries

1. Study Overview

Neonatal mortality, or death among newborns 0-28 days, now accounts for approximately 44% of global under 5-years child mortality.[1] Each year, there are 2.65 million stillbirths,[2,3] and nearly 2.8 million newborn deaths, most of which are preventable. The majority of these deaths occur in low- and middle-income countries (LMICs). Intrapartum asphyxia is one of the three leading causes of neonatal mortality, and also underlies high rates of newborn morbidity[4]. Training in neonatal resuscitation (NR) reduces asphyxia-related newborn mortality and morbidity, but there are barriers to the effective implementation of NR programs in LMICs, which are exacerbated by gaps in data collection of key indicators and outcomes.

However, the high penetration of mobile smartphones and cellular network connectivity in urban areas, along with new mobile graphics processing capabilities, makes innovative simulation training, including mobile-phone based virtual reality (VR), potentially feasible even in LMICs. Mobile VR simulations can be engaged at the learner's convenience, on their own smartphone, with game-based automated feedback that is ideal for episodic learning. Mobile VR training provides an opportunity to address challenges related to (a) maintenance of neonatal resuscitation knowledge and skills over time; (b) inconsistent migration of neonatal resuscitation skills into actual clinical practice; and (c) lack of a standardized dissemination to support neonatal resuscitation in LMICs.

We propose to use mobile VR simulations and a DHIS2-integrated mobile data collection platform as a tool to leapfrog over the current challenges to obtaining and maintaining NR knowledge and skills among health care providers (HCPs) in LMICs including lack of infrastructure, staff shortages, and high rates of staff turnover. Our hypotheses are that mobile phone-based VR training and digital data collection will provide: 1) effective training in neonatal resuscitation 2) an easy method to maintain skills, and 3) a less costly option of neonatal resuscitation training than current methods.

Methodology: To test these hypotheses we will conduct a randomized controlled trial of the VR neonatal resuscitation module in healthcare providers in Nigeria and Kenya to assess the impact of module on neonatal resuscitation performance.

Outcome variables: We will assess healthcare provider knowledge and performance of neonatal resuscitation in educational (simulation) and clinical settings and compare this performance across study groups.

For peer review only - http://bmjopen.bmj.com/site/about/guidelines.xhtml

2. BACKGROUND

Helping Babies Breathe (HBB) is a neonatal resuscitation training program for health care providers (HCPs) in LMICs. Since its global launch in June 2010, HBB has rolled out in over 80 countries worldwide. Helping Babies Breathe (HBB) training is conducted using a dissemination model called “training of trainers” (TOTs), which involves one to three days of in-person training of future trainers or providers of neonatal resuscitation through the use of lectures, small group learning, hands-on practice among paired learners using low-tech manikins, and evaluation of participants’ knowledge and skills using a multiple choice questionnaire, skills checks, and objective structured clinical examinations. However, field studies demonstrate that initial HBB training alone is insufficient for transfer of skills to clinical practice.[5-7] In the absence of continued mentoring and a supportive environment after the course, there is often a failure, among HBB providers, to consolidate and maintain the required knowledge, skills, and performance competencies which are required in order to perform high-quality neonatal resuscitation (NR) during actual clinical practice.[8,9]

Access to mobile phones, including smart phones and devices, has become nearly ubiquitous in LMICs allowing the introduction of e-learning that increases availability, scalability, flexibility and efficiency while decreasing marginal costs for training[10,11]. Evidence shows that e-learning is generally as effective as traditional teaching methods[12]. Under the right conditions, e-learning has the ability to promote innovations in design and distribution[13,14], including interactive learning, problem based learning, and standardized feedback[14-16]. Computer-based simulations, a subset of e-learning strategies, are increasingly used for self-directed learning before, or after attending an in-person course, e.g. HeartCode® ACLS, HeartCode® PALS and NRP eSim® programs.

Mobile VR simulations can be delivered on mobile phones as *screen-based* experiences transformed into *immersive* experiences with the use of a low-cost VR headset. Typically, they are brief, engaging, interactive, experiences that are relevant to clinical practice with the goal of fostering experiential learning that transforms attitudes and behaviors. VR simulations use a strategy of deliberate practice similar to the Ericsson’s deliberate practice paradigm,[17] in which learners deliberately practice their skills with expert coaching and feedback until they are able to perform the skills with minimal coaching. In games, automated feedback is tailored to the learner’s performance to ensure that practice occurs until the learner achieves the skills needed to move on to the next level of difficulty[18].

Integrating mobile VR training and digital data collection with neonatal resuscitation training in LMICs will address the challenges of (a) maintenance of knowledge and skills over time; (b) inconsistent migration of competencies into actual clinical practice; and (c) lack of a standardized, integrated, bi-directional data collection and information dissemination platform to support training in LMICS. The use of mobile VR simulations in HBB programs potentially

For peer review only - http://bmjopen.bmj.com/site/about/guidelines.xhtml

represents increased access to training, opportunities for self-directed learning, and less costly skill maintenance.

Investigators at the University of Washington have co-developed a virtual Helping Babies Breathe (eHBB) training simulation with Oxford University on the Life-Saving Instruction for Emergencies (LIFE) platform. A game-like approach utilizing a 3D game engine allows the user to experience an immersive and engaging training experience that has been adapted for the proposed eHBB (electronic Helping Babies Breathe) module. eHBB will run on a mobile phone but will also include the capability to work with low-cost VR headsets. In addition, investigators at Indiana University are customizing the mHBS (mobile Helping Babies Survive) application, an expanded version of an existing open-source data collection application mHBB to deliver the eHBB training module. The integrated eHBB and mHBS module will be used to augment initial and refresher training on key NR knowledge, skills and competencies among HCPs in LMICs.

3. Study Objective

The primary objective is to assess the impact of mobile VR simulation used before and after initial NR training on neonatal resuscitation educational indicators and performance outcomes in healthcare providers, in comparison to traditional HBB training.

4. Study Procedures

4.1 Study Participants

Our overall target sample size is 250 participants, but we will recruit up to 280 total participants across two international sites with an estimated 140 participants per site to account for attrition. Potential study participants will be identified from individuals registered to take an in-person HBB course or because they work or are assigned rotations in the labor/delivery wards of participating facilities.

4.2 Study Facilities

Participants will be recruited at study sites in Nigeria and Kenya. Potential study sites currently identified include Lagos University Teaching Hospital and referring facilities: Randle General Hospital, Surulere, and Federal Medical Centre, Ebute-Meta, Lagos Island Maternity Hospital, Lagos Island, Harvey Rd. Health Center, Shomolu General Hospital, Mushin General Hospital, Isolo General Hospital, Regina Mundi Hospital, Mushin, R-Jolad Specialist Hospital, Lagos, Nigeria. These facilities were selected on the basis of delivery volumes; number of nursing staff; presence of a neonatal unit; and/or association with a nursing and/or midwifery training program.

4.3 Inclusion Criteria

All HCPs who provide neonatal resuscitation to inborn or outborn infants and provide study consent. Key stakeholders in healthcare administration and community-based stakeholders will

For peer review only - http://bmjopen.bmj.com/site/about/guidelines.xhtml

also be recruited to participate in key informant interviews and focus group discussions.

4.4 Exclusion Criteria

HCPs who have received NR training less than one year before enrollment in the study; individuals who do not provide neonatal resuscitation as part of their duties; or will be unavailable or unwilling to participate in follow-up study activities throughout the 6-month post-initial training period will be excluded.

4.5 Recruitment

Site coordinators or research assistants will identify potential study participants from HCPs working in study facilities that meet eligibility criteria and are enrolled in a study HBB course.

A convenience sample of key stakeholders within Nigeria and Kenya, such as health facility administrators and community-based stakeholders will also be recruited to participate in key informant interviews and focus group discussions. (Appendix 3, Recruitment information)

4.6 Informed Consent

Site coordinators or research assistants will obtain informed consent from potential study participants. Each site is to provide their consent form to each study participant to be signed prior to randomization. (Appendix 4, Consent form).

4.7 Randomization/allocation procedure

Study numbers will be randomly generated, via a computer-generated algorithm for the VR, Educational (NR) video only, or Control (standard HBB TOT) groups. The eHBB study will be using block randomization based on provider experience: novice (no previous HBB training) and non-novice (any previous HBB training) to ensure that recruited participants are evenly distributed into all arms and groups of the study.

4.8 Outcome Measures

The *HBB Knowledge check* is a standardized HBB tool, is an 18-item multiple choice questionnaire (MCQ) which assesses HCP knowledge regarding immediate care after birth, routine care, and basic neonatal resuscitation (Appendix 6, HBB Knowledge Check).

BMV Checklist: The bag-and-mask ventilation skills checklist (BMV) is a standardized HBB tool used to assess HCPs competency on the provision of positive pressure ventilation with a bag-and-mask device (Appendix 7, Simulation Checklists).

Standardized simulations/OSCES: The HBB 2nd edition curriculum has two OSCEs, called OSCE A and OSCE B. These standardized simulations involve a series of time-sensitive tasks which happen in rapid succession and follow the steps of the HBB Action Plan (Appendix 7, Simulation Checklists).

For peer review only - http://bmjopen.bmj.com/site/about/guidelines.xhtml

Delivery Checklist: Routine newborn care provided by participating healthcare providers will be observed by research assistants using a standardized checklist.

5. Study Interventions

5.1 Pre-course interventions

Site coordinators or research assistants will provide links and airtime to download the mHBS/DHIS-2 application to their phone or a study device.

Pre-survey: The baseline HBB knowledge check and a demographic survey with questions regarding age, gender, area on duty, years of experience, previous health care, and NR training, time elapsed since NR previous training, experience/comfort with using computers, experience/comfort with video games, smartphone or tablet device ownership, will be administered through the mHBS application.

Completion of the pre-survey and baseline HBB knowledge check will "unlock" the study group assignment.

Figure 2. Study Timeline

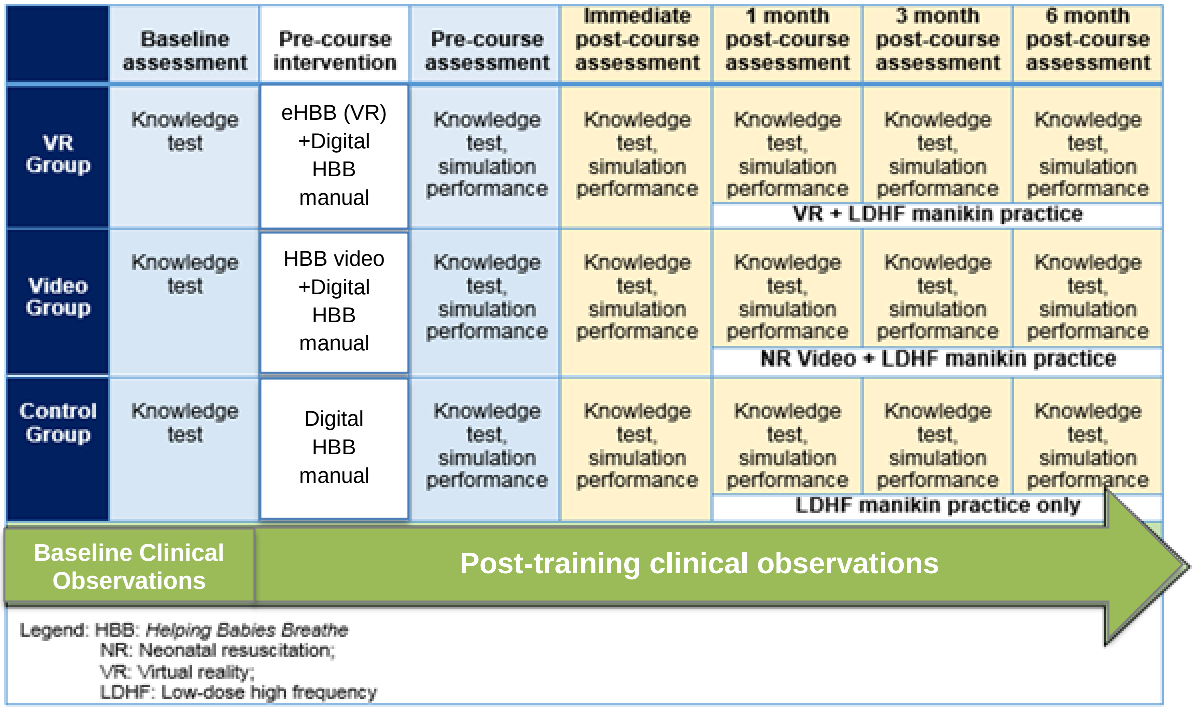

For peer review only - http://bmjopen.bmj.com/site/about/guidelines.xhtml

Study group assignment: Participants will be given access to study materials based on their group assignment of VR (eHBB), NR video or Control groups for review before their in-person HBB course. All groups will receive digital versions of the HBB manual accessible through the mHBS platform.

5.2 PRE-COURSE ASSESSMENTS

During the pre-course period, study personnel will visit study facilities on a weekly basis (or more frequently) to observe delivery practices, number of deliveries, and will conduct a needs assessment on the preparedness of study facility and personnel to provide neonatal resuscitation.

- All study facilities will be provided with resuscitation kits as needed, based on the initial HBB equipment and resource mapping, which will be conducted during the clinical observations baseline period, before in-person HBB training courses.

The research assistant will also perform baseline knowledge tests, bag-mask ventilation (BMV) skills and standardized simulation performance assessments in all participants using the NeoNatalie Advanced Simulator (Figure 3).

Figure 3. Neonatalie Advanced Manikin (Laerdal)

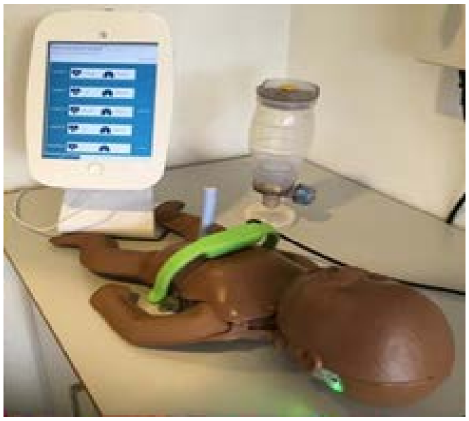

Participant performance during the simulation will be tracked using the Simulation (BMV and OSCE) Checklists by the research assistant and by the NeoNatalie Advanced simulator with standardized performance-based feedback provided to each participant. The manikin stores training data in a database that can be accessed remotely. When feasible, research assistants doing the performance and clinical observations will be blinded to the allocation of the HCPs whom they are assessing.

5.3 HELPING BABIES BREATHE 2ND EDITION COURSE

All study participants will complete a traditional in-person HBB Second Edition provider training course using a NeoNatalie low-fidelity manikin training kit and provider training materials. All participants will receive course attendance fees and a HBB 2nd edition course completion certificate.

HBB corner: All facilities will be encouraged to set up a HBB corner for manikin-based low-dose, high-frequency practice per current HBB program recommendations. All participants in all groups will be encouraged to perform low dose, high frequency (LDHF) manikin-based practice at facility-based HBB corners per current HBB recommendations. The frequency with which

For peer review only - http://bmjopen.bmj.com/site/about/guidelines.xhtml

participants interact with the manikins will be tracked using self-report on the mHBS app and paper logs at the HBB corners, and with trackers if feasible.

Figure 1. Study Diagram

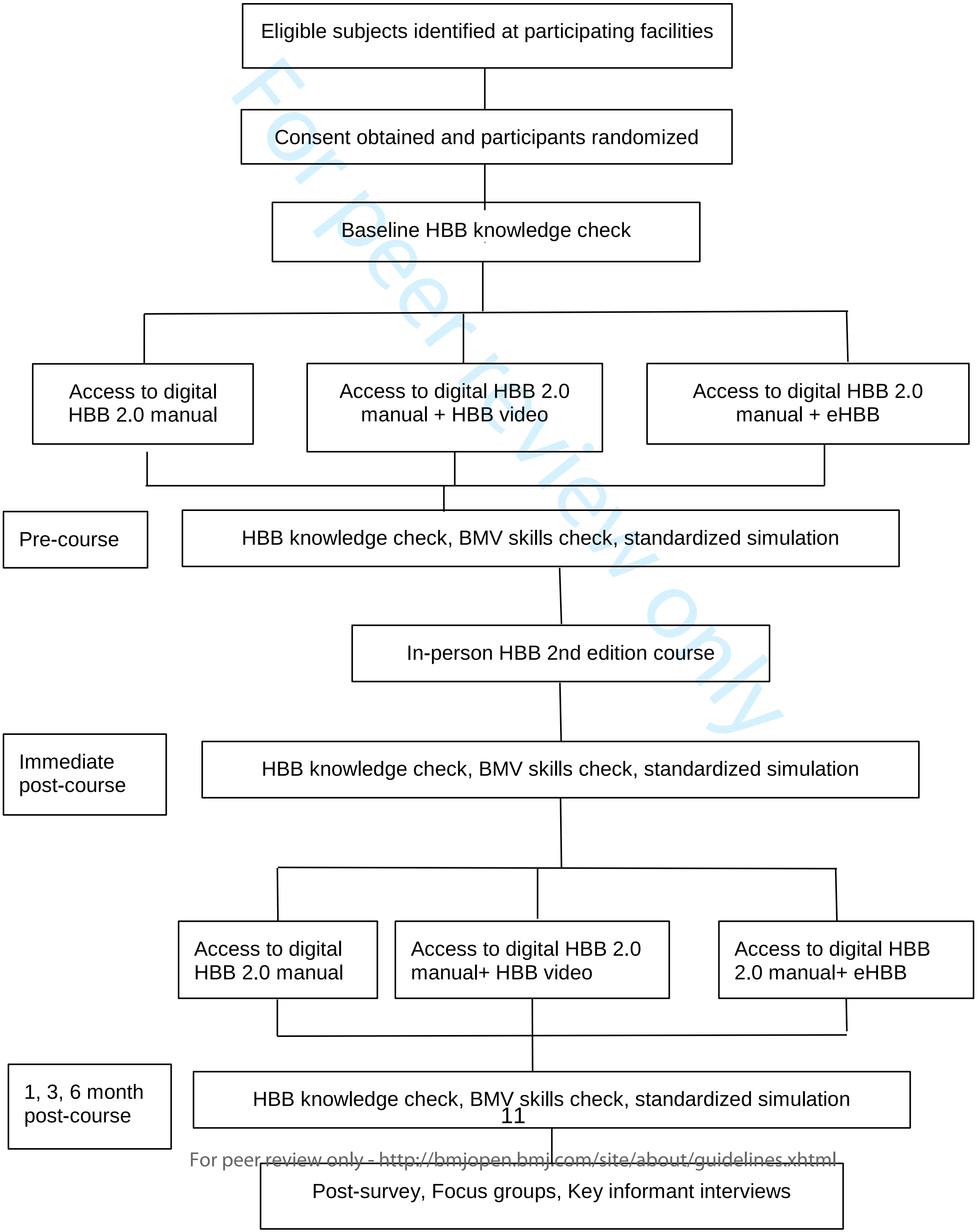

For peer review only

For peer review only - http://bmjopen.bmj.com/site/about/guidelines.xhtml

5.4 Immediate post-course assessments

Immediately after the HBB course, the research assistant will perform baseline knowledge tests, bag-mask ventilation (BMV) skills and standardized simulation performance assessments) in all participants using the NeoNatalie Advanced Simulator to conduct standardized simulation performance assessments.

Participant performance during the simulation will be tracked using the BMV and OSCE Checklists by the research assistant and by the NeoNatalie Advanced simulator with standardized performance-based feedback provided to each participant. The manikin stores training data in a database that can be accessed remotely. Where feasible, research assistants doing the performance and clinical observations will be blinded to the allocation of the HCPs whom they are assessing.

5.5 Follow up assessments

Participants will have ongoing access to the mHBS platform and will be sent reminders at 1, 3, and 6 months after initial NR training to take a follow-up NR knowledge test and to again participate in standardized performance simulations of bag-mask ventilation (BMV) skills and standardized simulation performance assessments conducted by research assistants who will visit their hospital facility with the NeoNatalie Advanced Simulator.

To understand the impact of educational strategies on clinical practice, clinical observations of routine care provided by participating healthcare providers will be conducted by research assistants in participating facilities using a standardized Delivery Checklist. This includes data on newborn care at the time of delivery and resuscitation (if applicable). See attached checklist. We will also gather data regarding capacity for provision of NR within facilities (e.g., number of deliveries, availability of trained staff and NR equipment), presence/absence of HBB practice corners, and rates of NR training among HCPs). We will review delivery outcomes if available from existing databases such as the Neonatal Registry before and after the HBB training. The Neonatal Registry data will be deidentified by a staff member who is not a member of the study team before release. When possible, resuscitation debriefs will be encouraged using the resuscitation debrief form and the perinatal death audit form will be used to identify the cause of death when not specified (see attached). No identifiable patient data will be stored and study facilities and participants will be identified by only by study code number.

At the end of the 6-month follow up period, participants will be asked to complete a feedback survey, designed to capture metrics related to the accessibility and utility of VR for neonatal resuscitation initial and refresher training.

6. Study Incentives

All active study participants will be provided with travel (if applicable), course attendance fees and air-time to facilitate upload of study data to a secure study database during the study follow-up period. Automated reminders will be sent one week before, on the due date, and one week

For peer review only - http://bmjopen.bmj.com/site/about/guidelines.xhtml

after the activity is due. After the third automated reminder, two attempts will be made by the study investigators to contact the study participant using contact information provided on enrollment. If no contact is made, the study participant will be deemed inactive.

7. DATA MANAGEMENT

Neonatalie Advanced Manikin data collected during simulation sessions will be identified by study ID only. *HBB Knowledge check, BMV and OSCE Checklists* data will be entered into mHBS and RedCap. Data from paper records will be entered into RedCap after each session. Electronic copies of study data will be stored on a local password protected study computer and in a secure study database. The hard copy of all documents should be filed in a locked cabinet in the study office.

8. DATA ANALYSIS

Demographic data will be compared between VR, NR video, and control groups and assessed for group to group differences. Pass/Fail rates on the study evaluations: Knowledge test, bag-and-mask ventilation skills assessment (BMV), and standardized simulations of routine care and initial resuscitation (OSCE A) and prolonged resuscitation (OSCE B) done at the following time periods: Baseline, Pre-course, Immediate post-course, and at 1 month, 3 months, and 6 months in the follow up period will be calculated and compared between groups. The primary endpoint is the 6-month post-baseline evaluation. We will perform an analysis of covariance with each six-month score regressed on the baseline score of each subject and compared among the three groups.

Two post-hoc statistical tests will be performed to ascertain possible statistically significant differences between VR and Video groups with the Control group at each time point. A secondary analysis will take full advantage of the longitudinal nature of the data (see Figure 9). Here, we posit a structural response model where skills will be initially acquired uniformly by all subjects (at the immediate post-course assessment after face-to-face training) but retention of these skills will dissipate rapidly over time in the Control group, but less precipitously in the other two groups, with the least deterioration of knowledge and skills in the VR group. The statistical modeling approach in this case will be through a change-point model with random intercept and slope. The main statistical test will be the assessment of the statistical significance of the interaction between group and time, during the second period (i.e., after the change or inflection point in the curve).

8.1 SAMPLE SIZE CALCULATIONS

We hypothesize that there will be a minimum 20% difference in the proportion of subjects who pass OSCE A and OSCE B, via a one-sided test at the 6-month evaluation. The desired power is 80% taking into account that two comparisons (VR vs. Controls and Video vs. Controls) will be carried out with the hypothesis that the proportions of subjects in the VR group will demonstrate performance metrics that are higher (by at least 20%) than the control group.

For peer review only - http://bmjopen.bmj.com/site/about/guidelines.xhtml

The alpha level has been halved so as not to inflate the type-1 error rate (of 5% overall) so 83 participants per group or a total of 249 participants will be sufficient to detect this difference under all the scenarios considered here. However, to account for study dropout, we will recruit up to 280 participants across the two country sites. We expect that the power generated by the ANCOVA procedure will be even higher as well as the power generated by the longitudinal (secondary) change-point analyses will be much more sensitive (powerful) to detect differences in each pairwise comparison. Thus the proposed sample size will likely be able to detect difference in skill retention with very high power.

9. QUALITATIVE STUDIES

This will consist of focus groups and key informant interviews led by an experienced facilitator utilizing semi-structured questionnaires. We will conduct 4-6 focus groups each consisting of 6-8 study participants and up to 15-20 key informant interviews among NR trained HCPs enrolled in the study and a convenience sample of key stakeholders within Nigeria such as health facility administrators and community-based stakeholders.

Domains addressed will include: what exposure to VR do healthcare providers have currently and what are their thoughts about learning from VR? How and when do healthcare providers use VR and what are their perspectives on it? What are the barriers and facilitators to using VR for initial and refresher training?

Sessions will be digitally recorded and transcribed. The data will be organized using qualitative analysis software. Qualitative data will be analyzed by two study investigators experienced in thematic analysis. Investigators will independently review all FGD transcripts to identify initial themes then work together to build consensus on all major themes. They will then independently code all transcripts through an iterative process using thematic content analysis[19]. As new codes are identified, the coding scheme will be refined using the grounded theory constant comparative method[20].

Investigators will meet regularly to compare and discuss codes, resolve disagreements, and come to consensus on discordantly coded data. Saturation will be achieved after no new themes emerge from the data. Codes within and across transcripts will be compared and synthesized into overarching themes that reflect the perspectives of healthcare providers on the use of technology and VR for training. A random sample of transcripts will be double coded to ensure agreement between coders. The data will be used to prepare a report on the facilitators and barriers to VR training for NR initial and refresher training in LMICs from the perspective of participants and stakeholders. The results will be shared with a subset of the participants for verification.

For peer review only - http://bmjopen.bmj.com/site/about/guidelines.xhtml

10. References

1. Chou D, Daelmans B, Jolivet RR, Kinney M, Say L. Ending preventable maternal and newborn mortality and stillbirths. *BMJ.* 2015;351.
2. Sharma G, Mathai M, Dickson K, et al. Quality care during labour and birth: a multi-country analysis of health system bottlenecks and potential solutions. *BMC Pregnancy Childbirth.* 2015;15(Suppl 2):S2.
3. Enweronu-Laryea C, Dickson K, Moxon S, et al. Basic newborn care and neonatal resuscitation: a multi-country analysis of health system bottlenecks and potential solutions. *BMC Pregnancy Childbirth.* 2015;15(Suppl 2):S4.
4. Lawn JE, Blencowe H, Oza S, et al. Every Newborn: progress, priorities, and potential beyond survival. *The Lancet.* 2014;384(9938):189-205.
5. Ersdal H, Singhal N. Resuscitation in resource-limited settings. Paper presented at: Seminars in Fetal and Neonatal Medicine2013.
6. Ersdal H, Vossius C, Bayo E, et al. A one-day "Helping Babies Breathe" course improves simulated performance but not clinical management of neonates. *Resuscitation.* 2013;84(10):1422-1427.
7. Mduma E, Ersdal H, Svensen E, Kidanto H, Auestad B, Perlman J. Frequent brief on-site simulation training and reduction in 24-h neonatal mortality—An educational intervention study. *Resuscitation.* 2015;93:1-7.
8. Bang A, Patel A, Bellad R, et al. Helping Babies Breathe (HBB) training: What happens to knowledge and skills over time? *BMC Pregnancy Childbirth.* 2016;16(1):364.
9. Bang A, Patel A, Bellad R, et al. Helping Babies Breathe (HBB) training: What happens to knowledge and skills over time? *BMC Pregnancy and Childbirth.* 2016;16(1):364.
10. Nagunwa TP, Lwoga ET. Developing an eLearning strategy to implement medical competency based curricula: Experiences from Muhimbili University of Health and Allied Sciences. Paper presented at: Education and e-Learning Innovations (ICEELI), 2012 International Conference on2012.
11. Frehywot S, Vovides Y, Talib Z, et al. E-learning in medical education in resource constrained low-and middle-income countries. *Human resources for health.* 2013;11(1):4.
12. Harun MH. Integrating e-learning into the workplace. *The Internet and higher education.* 2001;4(3):301-310.
13. Ruggeri K, Farrington C, Brayne C. A global model for effective use and evaluation of e-learning in health. *Telemedicine and e-Health.* 2013;19(4):312-321.
14. Joynes C. Distance learning for health: what works-A global review of accredited post-qualification training programmes for health workers in low and middle Income Countries. *London Int Dev Cent.* 2011;1.
15. Sweigart LI, Umoren RA, Scott PJ, et al. Virtual TeamSTEPPS® Simulations Produce Teamwork Attitude Changes Among Health Professions Students. *Journal of Nursing Education.* 2016;55(1):31-35.
16. Umoren RP, JA; Sweigart L; Rybas, N; Gossett, E; Johnson, M; Allen, M; Scott, PJ; Truman, B; Das, R. TEAMSTEPPS VIRTUAL TEAMS: Interactive virtual team training and practice for health professional learners *accepted for publication in Creative Nursing.*
17. Ericsson KA. Deliberate practice and the acquisition and maintenance of expert performance in medicine and related domains. *Academic medicine.* 2004;79(10):S70-S81.

For peer review only - http://bmjopen.bmj.com/site/about/guidelines.xhtml

18. Kapp KM. *The gamification of learning and instruction: game-based methods and strategies for training and education.* John Wiley & Sons; 2012.
19. Charmaz K. *Constructing grounded theory: A practical guide through qualitative analysis.* Sage; 2006.
20. Glaser BG. The constant comparative method of qualitative analysis. *Social problems.* 1965;12(4):436-445.

For peer review only

APPENDICES

Appendix 1. Abbreviations/Definition of Terms

AAP = American Academy of Pediatrics
BMGF = Bill and Melinda Gates Foundation
BMV = bag-and-mask ventilation
DHIS2 = District Health Information Software
eHBB = mobile/virtual reality Helping Babies Breathe
ECEB = Essential Care for Every Baby
ECSB = Essential Care for Small Babies
ENAP = Essential newborn action plan
ETAT+ = Emergency triage, assessment, and Treatment plus admission
HBB = Helping Babies Breathe
HBS = Helping Babies Survive
HCPs = Health care providers
LDHF = Low dose, high-frequency
LIFE = Life-saving Instruction for Emergencies
LMICs = Low/Middle income countries
mHBS = mobile Helping Babies Survive
MOH = Ministry of Health
MNCH = maternal newborn child health
NMR = neonatal mortality rate
NR = neonatal resuscitation
NRP = Neonatal Resuscitation Program
OSCE = Objective Structured Clinical Examination
PPV = positive-pressure ventilation
TOTs = Training of Trainers
VR = virtual reality
WHO = World Health Organization

Definition of Terms

3D simulation: These screen-based training scenarios are similar to the NRP eSIM® scenarios or the LIFE mobile game that enables learners practice the algorithm (e.g., “The Action Plan”) and reinforces knowledge of NR pathways but do not require a VR headset.

VR simulation: These training scenarios enable learners to practice both the NR algorithm and skills required for NR such as with the LIFE immersive VR NR game using a VR headset with or without hand controllers, e.g. Google cardboard, Google Daydream, Oculus Rift or HTC Vive.

Educational video: This is a video showing recommended NR algorithms, skills, and/or performance competencies related to preparation for delivery, immediate care at birth and/or successful newborn resuscitation using recommended HBB practices, such as videos offered by Global Health Media.

For peer review only - http://bmjopen.bmj.com/site/about/guidelines.xhtml

APPENDIX 2. STUDY DIAGRAM

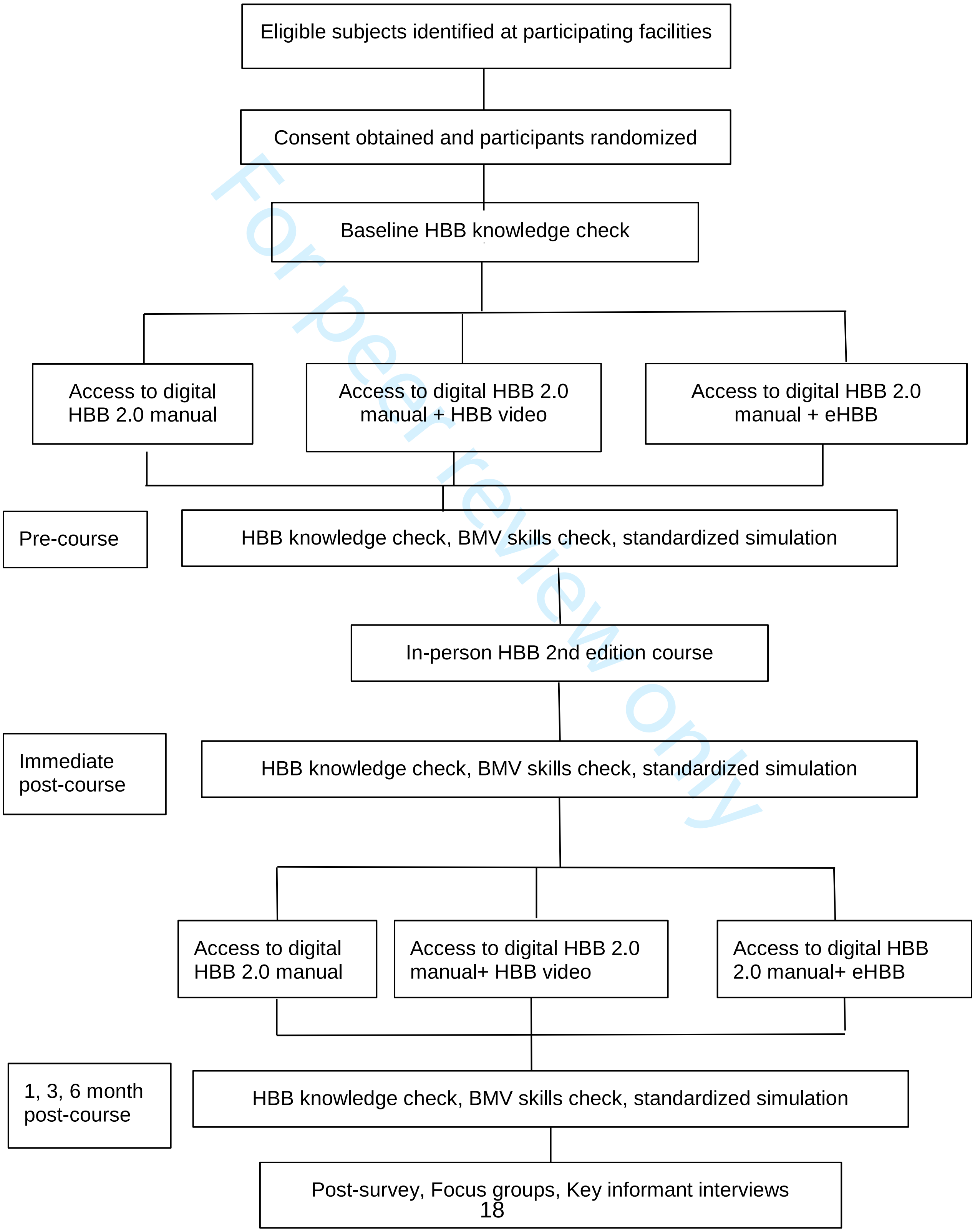

For peer review only

Appendix 3. Recruitment Information

You are invited to participate in the testing of a new mobile app for training on newborn resuscitation using the Helping Babies Breathe program.

We are recruiting health care workers and administrators with and without experience in using the Helping Babies Breathe program to assist with providing feedback on the application, particularly on whether it is easy to use and understand and whether it could be used in the future for training.

If you agree to participate, you will be assigned to one of three groups, eHBB, video or control group. You can use your own phone or a study phone to view the application using a simple virtual reality headset such as Google cardboard.

You will also receive training in the Helping Babies Breathe, 2nd edition program and your knowledge and skills in neonatal resuscitation will be observed by trained study personnel using a new type of manikin called the Neonatalie Advanced manikin. We will also ask you to answer questions about your experience either individually or as a group to help us understand your perspective on using these applications.

You will also receive a data credit to your phone as a thank you for your participation each month that you are in the study, for up to a six month period.

For peer review only

For peer review only - http://bmjopen.bmj.com/site/about/guidelines.xhtml

Appendix 4. Consent Form

For peer review only

For peer review only - http://bmjopen.bmj.com/site/about/guidelines.xhtml

Approved
8/7/2018
UW HSD IRB

INFORMATION SHEET FOR STAKEHOLDER INTERVIEWS

eHBB: virtual-reality training game for resuscitation of newborns

Researchers: <Site PI, Department, Institution, Contact information>
Study PI: Dr. Rachel A. Umoren, Pediatrics, University of Washington, 202-543-3200

Researchers' statement

We are asking you to be in a research study. The purpose of this consent form is to give you the information you will need to help you decide whether to be in the study or not. Please read the form carefully. You may ask questions about the purpose of the research, what we would ask you to do, the possible risks and benefits, your rights as a volunteer, and anything else about the research or this form that is not clear. When we have answered all your questions, you can decide if you want to be in the study or not. This process is called "informed consent." We will give you a copy of this form for your records.

PURPOSE OF THE STUDY

Virtual reality has been used extensively in industry for training in various domains. We are conducting a research project to evaluate "eHBB", a mobile phone based virtual reality (VR) simulation of the *Helping Babies Breathe* program integrated with mHBS/DHIS2 for training healthcare workers on newborn resuscitation. The goal of this study is to explore the perceptions of stakeholders on VR simulation for healthcare training.

STUDY PROCEDURES

We would like you to participate in a 30-minute interview or one-hour focus group discussion during which you will have an opportunity to use a new VR application called eHBB and answer questions on your perceptions of VR-based training with DHIS2 data collection. If you agree, the interview will be digitally recorded for analysis by researchers. There will not be any personal questions. Participation in the study is completely voluntary. You may refuse to answer any question or item. You are free to not participate if you so choose. You can stop at any time without penalty.

RISKS, STRESS, OR DISCOMFORT

Some individuals experience motion sickness with using VR. Using VR is not recommended for individuals with seizure disorders. If you experience discomfort, please stop using the eHBB VR application and inform the study coordinator immediately. All study information will be kept confidential. There is a small risk of loss of confidentiality. However every effort will be made to minimize this risk by using password protection and secure storage of study records. Data collected during the study will be kept indefinitely and deidentified data may be shared with other researchers.

BENEFITS OF THE STUDY

There are no anticipated benefits to participating in this study.

SOURCE OF FUNDING

The development of the eHBB and mHBS apps is being supported by the Bill and Melinda Gates Foundation.

CONFIDENTIALITY OF RESEARCH INFORMATION

All of the information you provide will be confidential. Identifier data and observation data will be collected but they will not be linked. Government or university staff may sometimes review studies such as this one to make sure they are being done safely and legally. If a review of this study takes place, your records may be examined. The reviewers will protect your privacy. There are some limits to this protection.

OTHER INFORMATION

You may refuse to participate and you are free to withdraw from this study at any time without penalty or loss of benefits to which you are otherwise entitled. There are no costs to you for participating in the study. You will be provided with travel costs (if applicable), and internet data for downloading and using the application. There is no compensation for participating in the study. If you have questions, complaints or concerns about this study, you can contact Rachel Umoren at rumoren@uw.edu.

For peer review only

For peer review only - http://bmjopen.bmj.com/site/about/guidelines.xhtml

Approved
8/7/2018
UW HSD IRB

CONSENT FORM

eHBB: virtual-reality training game for resuscitation of newborns

Researchers: <Site PI, Department, Institution, Contact information>

Study PI: Dr. Rachel A. Umoren, Pediatrics, University of Washington, 202-543-3200

Researchers' statement

We are asking you to be in a research study. The purpose of this consent form is to give you the information you will need to help you decide whether to be in the study or not. Please read the form carefully. You may ask questions about the purpose of the research, what we would ask you to do, the possible risks and benefits, your rights as a volunteer, and anything else about the research or this form that is not clear. When we have answered all your questions, you can decide if you want to be in the study or not. This process is called "informed consent." We will give you a copy of this form for your records.

For peer review only

PURPOSE OF THE STUDY

Virtual reality has been used extensively in industry for training in various domains. We are conducting a research project to evaluate "eHBB", a mobile phone based virtual reality (VR) simulation of the *Helping Babies Breathe* program for training healthcare workers on newborn resuscitation. The goal of this study is to assess the impact of mobile VR simulation used before and after initial neonatal resuscitation training on educational outcomes, in comparison to watching a neonatal resuscitation video or traditional HBB training.

STUDY PROCEDURES

If you agree to participate, you will be randomly assigned to one of three study groups: eHBB (VR) group, neonatal resuscitation Video group, and standard HBB training material (Control) group. Participants will attend a full-day HBB 2nd edition course free of charge and receive a course completion certificate. Study participants will be given access to the study intervention for their group before the HBB class and for six months after the class. Participants' knowledge and skills in neonatal resuscitation will be assessed through standardized tests and simulations by trained study observers before, immediately after, and at 1, 3, and 6 months following the HBB 2nd edition course. Study researchers may also visit participating facilities to assess readiness for neonatal resuscitation and delivery practices. The estimated time for each simulation session is 20 minutes. The eHBB simulation and video takes less than 10 minutes per viewing session.

Regardless of group assignment, participants will have the opportunity to view the eHBB and mHBS/DHIS2 applications and provide feedback on their perceptions of VR-based training during one-hour focus groups and interviews at the end of the study. Data captured during simulations, interviews and focus group discussions will be digitally recorded for analysis by researchers. There will not be any personal or sensitive questions.

Participation in the study is completely voluntary. You may refuse to answer any question or item. You are free to not participate if you so choose. You can stop at any time without penalty.

RISKS, STRESS, OR DISCOMFORT

Some individuals experience motion sickness with using virtual reality. Using virtual reality is not recommended for individuals with seizure disorders. If you experience discomfort, please stop using the eHBB VR application and inform the study coordinator immediately. All study information will be kept confidential. There is a small risk of loss of confidentiality. However every effort will be made to minimize this risk by using password protection and secure storage of study records. Data collected during the study will be kept indefinitely and deidentified datasets may be shared with other researchers.

For peer review only - http://bmjopen.bmj.com/site/about/guidelines.xhtml

Approved
8/7/2018
UW HSD IRB

BENEFITS OF THE STUDY

Healthcare workers participating in the study may gain added expertise in newborn resuscitation.

SOURCE OF FUNDING

The development of the eHBB and mHBS apps is being supported by the Bill and Melinda Gates Foundation.

CONFIDENTIALITY OF RESEARCH INFORMATION

All of the information you provide will be confidential. Identifier data and observation data will be collected but they will not be linked. Government or university staff sometimes review studies such as this one to make sure they are being done safely and legally. If a review of this study takes place, your records may be examined. The reviewers will protect your privacy. The study records will not be used to put you at legal risk of harm. There are some limits to this protection.

OTHER INFORMATION

You may refuse to participate and you are free to withdraw from this study at any time without penalty or loss of benefits to which you are otherwise entitled.

There are no costs to you for participating in the study. You will be provided with travel costs (if applicable), course attendance fees, and internet data for downloading and using the application. There is no compensation for participating in the study.

RESEARCH-RELATED INJURY

If you have questions, complaints or concerns about this study, you can contact Rachel Umoren at rumoren@uw.edu. The UW does not normally provide compensation for harm except through its discretionary program for medical injury. You do not waive any right to seek payment by signing this consent form.

Printed name of researcher   Signature of researcher   Date

Subject's statement

This study has been explained to me. I volunteer to take part in this research. I have had a chance to ask questions. If I have questions later about the research, or if I have been harmed by participating in this study, I can contact one of the researchers listed on the first page of this consent form. If I have questions about my rights as a research subject, I can call the UW Human Subjects Division at (206) 543-0098 or call collect at (206) 221-5940. I will receive a copy of this consent form.

Printed name of participant   Signature of participant   Date

Copies to: Researcher
Participant

For peer review only - http://bmjopen.bmj.com/site/about/guidelines.xhtml

APPENDIX 5. PARTICIPANT SURVEYS

For peer review only

For peer review only - http://bmjopen.bmj.com/site/about/guidelines.xhtml

Demographic Survey

Study ID ______________________

Please complete the following survey

1. Age range

◯ less than 21 years ◯ 21 - 30 years ◯ 31 - 40 years ◯ 41 - 50 years ◯ More than 50 years

2. Sex

◯ Male ◯ Female

3. Profession

◯ Consultant physician
◯ Senior registrar
◯ Registrar
◯ Nurse
◯ Midwife
◯ Clinical Officer
◯ Other

profession_other

____

Please list your Degree/Certification in your area of practice

____

4. Years of practice since completion of training

◯ Less than 5 years ◯ 5 - 10 years ◯ 11 - 15 years ◯ 16 - 20 years ◯ More than 20 years

5. Current location of practice

◯ Nigeria ◯ Kenya ◯ Other

Other (please specify)

____

6. Health care level of practice

◯ Government (Tertiary care)
◯ Government (Secondary care)
◯ Government (Primary care)
◯ Private
◯ Faith-based facility

Healthcare Facility

____

For peer review only
For peer review only - http://bmjopen.bmj.com/site/about/guidelines.xhtml

7. Specialty (if applicable)

- ◯ General Paediatrics
- ◯ Subspecialty Paediatrics
- ◯ Obstetrics & Gynaecology
- ◯ Subspecialty O & G
- ◯ Other specialty

Other Specialty (please specify)

____

8. Does your institution/health facility have facilities for simulation based training?

◯ Yes ◯ No

9. In what capacity does your institution use simulation-based training?

☐ Teaching ☐ Research ☐ Examination

10. Does your center have a skills-based simulation lab?

◯ Yes ◯ No

11. What is the skills-based simulation lab available for

☐ Skills practice, eg. HBB corner ☐ Teaching ☐ Research ☐ Examination

13. Which modality of simulation based training have you been exposed to?

- ☐ Manikin-based training (HBB)
- ☐ Manikin-based training (NRT)
- ☐ Manikin-based training (PALS)
- ☐ Manikin-based training (ENCC)
- ☐ Manikin-based training (BLS)
- ☐ NRP eSIM™
- ☐ HeartCode™ (PALS online course)
- ☐ Online Basic Life Support course
- ☐ Online ACLS course
- ☐ Virtual Reality Simulation (VR)
- ☐ Other (please specify below)

Other (please specify)

____

14. Are you aware of virtual reality simulation training?

◯ Yes ◯ No

15. When or where were you exposed to virtual reality simulation?

____

For peer review only

16. What are the challenges to online (computer-based or virtual reality) simulation?

- ☐ Lack of awareness about VR based simulation
- ☐ Lack of internet access
- ☐ Lack of standardized VR training modules
- ☐ Inconsistent power supply
- ☐ Lack of access to VR equipment and computers
- ☐ Other

Other (please specify)

____

18. Are you aware that VR simulation can be run on a mobile phone so that you can learn skills on your own time and pace.

◯ Yes ◯ No

19. What type of mobile phone device do you own or use? (Choose all that apply)

- ◯ a. Tablet (e.g. Ipad, tablets)
- ◯ b.Smart Phone (e.g. Iphone, Samsung, Techno, Nexus, Infinix etc.)
- ◯ c. Feature phone (e.g. Does some gprs based activities)
- ◯ d.Basic (e.g. Used for call and SMS only)

20. What is the manufacturer and model of your phone/mobile device?

____

21. If you are using an android enabled device, what android version does your device run (To find out, Goto |Settings->General->AboutDevice| and look for version number)

- ◯ a. Gingerbread (version 2.3)
- ◯ b.Ice Cream Sandwich (version 4.0)
- ◯ c. Jelly Bean (version 4.1 - 4.3)
- ◯ d.KitKat (Version 4.4)
- ◯ e. Lollipop (Version 5.0 - 5.1)
- ◯ f. Marshmallow (Version 6.0)
- ◯ g. Nougat (Version 7)
- ◯ h.Oreo (Version 8)
- ◯ i. Other

If Other, please specify

____

22. Do you use mobile device (phone or tablet) currently for your work?

◯ Yes ◯ No

Please describe how you use your phone for work

For peer review only

25. If all facilities were available, would you recommend online simulation for training healthcare workers in your country?

◯ a. Yes ◯ b. No

If all facilities were available, would you recommend online simulation for your center?

◯ a. Yes ◯ b. No

26. Please state your reason(s)

For peer review only

Follow Up Survey

Study ID ______________________________

Please tell us a little about your participation in deliveries and ongoing HBB training in the last 6 months.

Approximately how many deliveries have you participated in during the last 6 months?
- ◯ none
- ◯ 1-5
- ◯ 6-10
- ◯ 11-15
- ◯ 16-20
- ◯ 21-25
- ◯ >25

How many deliveries have you participated in during the last 6 months that required resuscitation (at least one: bag-mask ventilation, chest compressions, intubation)? ______________________________

Have you used the eHBB program in the last 6 months?
- ◯ No
- ◯ Yes

Have you watched the HBB neonatal resuscitation video in the last 6 months?
- ◯ No
- ◯ Yes

Please let us know about the quality of your training:

	Strongly disagree	Disagree	Neutral	Agree	Strongly agree
eHBB was easy to access	◯	◯	◯	◯	◯
eHBB was valuable for clinical practice	◯	◯	◯	◯	◯
eHBB was easy to navigate	◯	◯	◯	◯	◯
eHBB had realistic graphics	◯	◯	◯	◯	◯
eHBB provided valuable feedback	◯	◯	◯	◯	◯
the HBB video was easy to access	◯	◯	◯	◯	◯
the HBB video was valuable for clinical practice	◯	◯	◯	◯	◯
the HBB video was realistic	◯	◯	◯	◯	◯

Did you have any technical difficulties with the refresher training (eHBB or HBB videos) between your in person simulations?
- ◯ Yes
- ◯ No

If yes, please explain: ______________________________

Would you use this method of refresher training again?
- ◯ Yes
- ◯ No

For peer review only

If no, please explain: __________________________________

If you weren't able to complete your assigned refresher training to watch the HBB video or do the eHBB at the 1, 3 or 6 month mark from your HBB course, please tell us which barrier(s) contributed?

- ☐ I didn't receive a reminder
- ☐ The mHBS application didn't work
- ☐ I had trouble accessing the refreshers on mHBS
- ☐ I didn't have time to complete the refreshers
- ☐ I didn't find the refreshers relevant
- ☐ I didn't feel that I needed a refresher
- ☐ I didn't remember about the refreshers
- ☐ Other

If you chose other, please tell us about the barriers you experienced with the refreshers __________________________________

If you weren't able to complete your simulation practice at the 1, 3 or 6 month mark from your HBB course, please tell us which barrier(s) contributed?

- ☐ I didn't receive a reminder from the study personnel
- ☐ The practice location was not convenient
- ☐ I had trouble contacting the study personnel
- ☐ I didn't have time to complete the practice
- ☐ I didn't find the practice relevant
- ☐ I didn't feel that I needed the practice
- ☐ I didn't remember about the practice
- ☐ Other

If you chose other, please tell us about the barriers you experienced with attending the simulation practice __________________________________

Other comments or concerns: __________________________________

For peer review only

Appendix 6. HBB Knowledge Check

For peer review only

For peer review only - http://bmjopen.bmj.com/site/about/guidelines.xhtml

HBB Knowledge Check

Study ID ______________________

1. What should you do in The Golden Minute?
- ◯ a. Bathe the baby
- ◯ b. Deliver the placenta
- ◯ c. Evaluate the heart rate
- ◯ d. Help a baby breathe if necessary

2. To prepare for a birth
- ◯ a. You identify a helper and review the emergency plan
- ◯ b. You ask everyone but the mother to leave the area
- ◯ c. You prepare equipment only when you need it
- ◯ d. You do not need a helper

3 To prepare the area for delivery
- ◯ a. Open all the doors and windows to get fresh air
- ◯ b. Darken the room
- ◯ c. Make sure the area is clean, warm, and well-lighted
- ◯ d. Keep the room temperature cold

4. What should you do to keep the baby warm?
- ◯ a. Open all the windows
- ◯ b. Give the baby a bath after birth
- ◯ c. Place hot water bottles next to the baby's skin
- ◯ d. Place the baby skin-to-skin with mother

5. What should you do to keep the baby clean?
- ◯ a. Wash your hands before touching the baby and help
- ◯ mother wash her hands before breastfeeding
- ◯ b. Reuse the suction device before cleaning
- ◯ c. Keep the umbilical cord tightly covered
- ◯ d. Do not touch the baby

6. Which baby can receive routine care after birth?
- ◯ a. A baby who is not breathing
- ◯ b. A baby who is gasping
- ◯ c. A baby who is crying and/or breathing well
- ◯ d. A baby who is limp

7. Routine care for a healthy baby at birth includes
- ◯ a. Drying, removing the wet cloth, and bathing the baby
- ◯ b. Drying, removing the wet cloth, and positioning
- ◯ the baby skin-to-skin
- ◯ c. Bathing and putting clean clothes on the baby
- ◯ d. Drying and wrapping the baby in the wet cloth

8. When should the umbilical cord be clamped or tied and cut during routine care?
- ◯ a. After the placenta is delivered
- ◯ b. Around 1-3 minutes after birth
- ◯ c. Immediately after the baby is born
- ◯ d. Before a baby has cried

9. A baby is quiet, limp and not breathing at birth. What should you do?
- ◯ a. Dry the baby thoroughly
- ◯ b. Shake the baby
- ◯ c. Throw cold water on the face
- ◯ d. Hold the baby upside down

For peer review only

10. A newborn baby is quiet, limp and not crying. The baby does not respond to steps to stimulate breathing. What should you do next?	◯ a. Slap the baby's back<br>◯ b. Hold the baby upside down<br>◯ c. Squeeze the baby's ribs<br>◯ d. Begin ventilation
11. In which situation should a baby be suctioned?	◯ a. When a baby is crying at birth<br>◯ b. When a baby is crying but there is meconium in<br>◯ the amniotic fluid<br>◯ c. When you see secretions blocking the mouth and nose<br>◯ d. Before drying the baby
12. Suctioning a baby unnecessarily or frequently can	◯ a. Cause a baby to stop breathing<br>◯ b. Make a baby start coughing and breathing<br>◯ c. Stimulate a baby to cry<br>◯ d. Increase the baby's heart rate
13. Which of the following statements about ventilation with bag and mask is TRUE?	◯ a. The mask should cover the eyes<br>◯ b. Air should escape between the mask and face<br>◯ c. Squeeze the bag to produce gentle movement of the chest<br>◯ d. Squeeze the bag to give 80 to 100 breaths per minute
14. A baby's chest is not moving with bag and mask ventilation. What should you do?	◯ a. Stop ventilation<br>◯ b. Reapply the mask to get a better seal<br>◯ c. Slap the baby's back<br>◯ d. Give medicine to the baby
15. You can stop ventilation if	◯ a. A baby is blue and limp<br>◯ b. A baby's heart rate is slow<br>◯ c. A baby's heart rate is normal and the chest is not moving<br>◯ d. A baby's heart rate is normal and the baby is breathing or crying
16. A newborn baby's heart rate should be:	◯ a. Faster than your heart rate<br>◯ b. Slower than your heart rate<br>◯ c. Checked before drying the baby<br>◯ d. Checked only when the baby is crying
17. A baby who received ventilation	◯ a. Needs continued observation with mother<br>◯ b. Cannot be fed<br>◯ c. Always needs advanced care<br>◯ d. Should immediately receive antibiotics
18. When should the bag and mask and suction device be disinfected?	◯ a. After every use<br>◯ b. Only when they appear dirty<br>◯ c. Weekly<br>◯ d. Once a month

For peer review only

APPENDIX 7. SIMULATION CHECKLISTS

For peer review only

For peer review only - http://bmjopen.bmj.com/site/about/guidelines.xhtml

BMV Skill Check

Study ID ____________________

Complete this evaluation with participants before they attempt the OSCE evaluations.
- Read aloud the following instructions
- Use the comments below the numbered steps to score the performance
- Note the number of steps done correctly on the first attempt
- Read the feedback from Neonatalie Advanced ipad. Do not give any other feedback.

"You are attending the delivery of a term infant. You have prepared for the birth and tested the bag, mask, and suction device. You have dried and stimulated the baby, but the baby is not breathing. Show me how you will provide ventilation."

For peer review only

1. Begin to ventilate with bag and mask

	Done	Not done
Place the baby on the area for ventilation	☐	☐
Stand at the baby's head	☐	☐
Check that mask size is correct	☐	☐

2. Ventilate with bag and mask

	Done	Not done
Position the head slightly extended	☐	☐
Apply the mask to the face	☐	☐
Make a tight seal between the mask and the face	☐	☐
Squeeze the bag to produce gentle movement of the chest	☐	☐

3. Continue ventilation (for 1 minute)

	Done	Not done
Ventilate to produce gentle movement of the chest with each ventilation breath	☐	☐
Ventilate at 40 breaths/minute (30-50 breaths/minute acceptable)	☐	☐

The baby's chest has stopped moving with ventilation. Show me what you would do to improve ventilation.

4. Improve ventilation

	Done	Not done
Reapply mask	☐	☐
Reposition head	☐	☐
Clear mouth and nose of secretions	☐	☐
Open the mouth	☐	☐
Squeeze the bag harder	☐	☐

Score on first attempt ____________________

For peer review only

OSCE A

Study ID ______________________

Objective Structured Clinical Evaluations (OSCEs) can be used to determine whether participants have learned the essential steps to help a baby breathe. They can be used to verify that a participant knows enough to pass the course, or also as an exercise repeated regularly for practice. Most importantly, each completed evaluation should be used as an opportunity for the participant to review and learn.

Read the case scenario aloud to the participant. Provide the prompts shown in red. Indicate the baby's response to the participant's actions using the neonatal simulator or words if using a mannequin. For example, when the participants evaluate crying, show that the baby is not crying with a simulator. Say that the baby is not crying if using a mannequin. As you observe the participant, tick the boxes "Done" or "Not Done" for each activity. Apart from giving these prompts, keep silent during the evaluation.

After participants complete the OSCE, ask the 5 questions written below OSCE A. These questions will help the participants reflect on what actions they took and what they can do better the next time. Participants who can recognize their own mistakes will better remember the right steps to take the next time. Comment on the participant's performance only at the end of the case, after he/she has answered these 5 questions.

"I am going to read a role play case. Please listen carefully, and then show me the actions you would take. I will indicate the
baby's responses, but I will provide no other feedback until the end of the case."

"You are called to assist the delivery of a term baby. There are no complications in the pregnancy. The baby will be born in less than 10 minutes. Introduce yourself and prepare for the birth and care of the baby."

	Done	Not Done
Identifies a helper and reviews an emergency plan.	○	○
Prepares the area for delivery (warm, well-lighted, clean)	○	○
Washes hands	○	○
Prepares an area for ventilation and checks function of bag, mask and suction device	○	○

Prompt: After 5-7 minutes give baby to participant and say, "There is meconium in the amniotic fluid.
The baby is delivered onto the mother's abdomen. Show how you will care for the baby."

	Done	Not Done
Dries thoroughly	○	○
Removes wet cloth	○	○

For peer review only

Prompt: Show the baby is not crying. "There is meconium blocking the mouth."

	Done	Not Done
Recognizes baby is not crying	◯	◯
Positions head and clears airway	◯	◯
Stimulates breathing by rubbing the back	◯	◯

Prompt: Show the baby is breathing well (cries)

	Done	Not Done
Recognizes baby is crying and breathing well	◯	◯
Clamps or ties and cuts the cord	◯	◯
Positions skin-to-skin on mother's chest and puts on the head covering	◯	◯
Communicates with mother	◯	◯

Use the questions below to help the participant reflect on his or her own performance and then provide feedback.
1. What happened at the birth?
2. Did you follow the Action Plan?
3. What went well and what could have gone better?
4. What did you learn?
5. What will you do differently next time?

Comments

For peer review only

OSCE B

Study ID ______________________

Instructions to the facilitator: Read the below instructions for the case scenario.

"I am going to read a role play case. Please listen carefully, and then show me the actions you would take. I will indicate the baby's responses, but I will provide no other feedback until the end of the case."

"You are called to assist at the birth of 34 week (7-1/2 months) gestation baby. You have identified a helper, prepared an area for ventilation, washed your hands, and checked your equipment. The baby is born, and the amniotic fluid is clear.
Show how you will care for the baby."

	Done	Not Done
Dries thoroughly	◯	◯
Removes wet cloth	◯	◯

Prompt: Show the baby is not crying. "You do not see or hear secretions in the baby's mouth or nose."

	Done	Not Done
Recognizes baby is not crying	◯	◯
Stimulates breathing by rubbing the back	◯	◯

Prompt: Show the baby is not breathing

	Done	Not Done
Recognizes baby is not breathing	◯	◯
Cuts cord and moves to area for ventilation OR positions by mother for ventilation	◯	◯
Ventilates with bag and mask within The Golden Minute (at ____seconds)	◯	◯
Achieves a firm seal as demonstrated by chest movement	◯	◯
Time of effective ventilation (chest moving gently at _____ seconds)	◯	◯
Ventilates at 40 breaths/minute (30-50 acceptable)	◯	◯
Evaluates for breathing or chest movement	◯	◯

For peer review only

Prompt: Show the baby is not breathing.

	Done	Not Done
Recognizes baby is not breathing	◯	◯
Calls for help	◯	◯
Continues ventilation	◯	◯

Prompt: Say, "Please show what to do if the chest is not moving with ventilation." After one or more steps to improve ventilation, say "The chest is moving now."

	Done	Not Done
Reapplies mask	◯	◯
Repositions head	◯	◯
Clears secretions from the mouth and nose as needed	◯	◯
Opens mouth slightly	◯	◯
Squeeze bag harder	◯	◯

Prompt: Show the baby is not breathing; heart rate is normal.

	Done	Not Done
Recognizes baby is not breathing but heart rate is normal	◯	◯
Continues ventilation	◯	◯

Prompt: (After 3 minutes) Show the heart rate is 120 per minute and the baby is breathing.

	Done	Not Done
Recognizes baby is breathing and heart rate is normal	◯	◯
Stops ventilation	◯	◯
Provides close observation for the baby and communicates with the mother	◯	◯

Use the questions below to help the participant reflect on his or her own performance and then provide feedback.
1. What happened at the birth?
2. Did you follow the Action Plan?
3. What went well and what could have gone better?
4. What did you learn?
5. What will you do differently next time?

Comments

For peer review only

SUPPLEMENTARY FILE 2

SAMPLE CONSENT FORM

For peer review only

For peer review only - http://bmjopen.bmj.com/site/about/guidelines.xhtml

Approved
8/7/2018
UW HSD IRB

INFORMATION SHEET FOR STAKEHOLDER INTERVIEWS

eHBB: virtual-reality training game for resuscitation of newborns

Researchers: <Site PI, Department, Institution, Contact information>
Study PI: Dr. Rachel A. Umoren, Pediatrics, University of Washington, 202-543-3200

Researchers' statement

We are asking you to be in a research study. The purpose of this consent form is to give you the information you will need to help you decide whether to be in the study or not. Please read the form carefully. You may ask questions about the purpose of the research, what we would ask you to do, the possible risks and benefits, your rights as a volunteer, and anything else about the research or this form that is not clear. When we have answered all your questions, you can decide if you want to be in the study or not. This process is called "informed consent." We will give you a copy of this form for your records.

PURPOSE OF THE STUDY

Virtual reality has been used extensively in industry for training in various domains. We are conducting a research project to evaluate "eHBB", a mobile phone based virtual reality (VR) simulation of the *Helping Babies Breathe* program integrated with mHBS/DHIS2 for training healthcare workers on newborn resuscitation. The goal of this study is to explore the perceptions of stakeholders on VR simulation for healthcare training.

STUDY PROCEDURES

We would like you to participate in a 30-minute interview or one-hour focus group discussion during which you will have an opportunity to use a new VR application called eHBB and answer questions on your perceptions of VR-based training with DHIS2 data collection. If you agree, the interview will be digitally recorded for analysis by researchers. There will not be any personal questions. Participation in the study is completely voluntary. You may refuse to answer any question or item. You are free to not participate if you so choose. You can stop at any time without penalty.

RISKS, STRESS, OR DISCOMFORT

Some individuals experience motion sickness with using VR. Using VR is not recommended for individuals with seizure disorders. If you experience discomfort, please stop using the eHBB VR application and inform the study coordinator immediately. All study information will be kept confidential. There is a small risk of loss of confidentiality. However every effort will be made to minimize this risk by using password protection and secure storage of study records. Data collected during the study will be kept indefinitely and deidentified data may be shared with other researchers.

BENEFITS OF THE STUDY

There are no anticipated benefits to participating in this study.

SOURCE OF FUNDING

The development of the eHBB and mHBS apps is being supported by the Bill and Melinda Gates Foundation.

CONFIDENTIALITY OF RESEARCH INFORMATION

All of the information you provide will be confidential. Identifier data and observation data will be collected but they will not be linked. Government or university staff may sometimes review studies such as this one to make sure they are being done safely and legally. If a review of this study takes place, your records may be examined. The reviewers will protect your privacy. There are some limits to this protection.

OTHER INFORMATION

You may refuse to participate and you are free to withdraw from this study at any time without penalty or loss of benefits to which you are otherwise entitled. There are no costs to you for participating in the study. You will be provided with travel costs (if applicable), and internet data for downloading and using the application. There is no compensation for participating in the study. If you have questions, complaints or concerns about this study, you can contact Rachel Umoren at rumoren@uw.edu.

For peer review only - http://bmjopen.bmj.com/site/about/guidelines.xhtml

Approved
8/7/2018
UW HSD IRB

CONSENT FORM

eHBB: virtual-reality training game for resuscitation of newborns

Researchers: <Site PI, Department, Institution, Contact information>

Study PI: Dr. Rachel A. Umoren, Pediatrics, University of Washington, 202-543-3200

Researchers' statement

We are asking you to be in a research study. The purpose of this consent form is to give you the information you will need to help you decide whether to be in the study or not. Please read the form carefully. You may ask questions about the purpose of the research, what we would ask you to do, the possible risks and benefits, your rights as a volunteer, and anything else about the research or this form that is not clear. When we have answered all your questions, you can decide if you want to be in the study or not. This process is called "informed consent." We will give you a copy of this form for your records.

PURPOSE OF THE STUDY

Virtual reality has been used extensively in industry for training in various domains. We are conducting a research project to evaluate "eHBB", a mobile phone based virtual reality (VR) simulation of the *Helping Babies Breathe* program for training healthcare workers on newborn resuscitation. The goal of this study is to assess the impact of mobile VR simulation used before and after initial neonatal resuscitation training on educational outcomes, in comparison to watching a neonatal resuscitation video or traditional HBB training.

STUDY PROCEDURES

If you agree to participate, you will be randomly assigned to one of three study groups: eHBB (VR) group, neonatal resuscitation Video group, and standard HBB training material (Control) group. Participants will attend a full-day HBB 2nd edition course free of charge and receive a course completion certificate. Study participants will be given access to the study intervention for their group before the HBB class and for six months after the class. Participants' knowledge and skills in neonatal resuscitation will be assessed through standardized tests and simulations by trained study observers before, immediately after, and at 1, 3, and 6 months following the HBB 2nd edition course. Study researchers may also visit participating facilities to assess readiness for neonatal resuscitation and delivery practices. The estimated time for each simulation session is 20 minutes. The eHBB simulation and video takes less than 10 minutes per viewing session.

Regardless of group assignment, participants will have the opportunity to view the eHBB and mHBS/DHIS2 applications and provide feedback on their perceptions of VR-based training during one-hour focus groups and interviews at the end of the study. Data captured during simulations, interviews and focus group discussions will be digitally recorded for analysis by researchers. There will not be any personal or sensitive questions.

Participation in the study is completely voluntary. You may refuse to answer any question or item. You are free to not participate if you so choose. You can stop at any time without penalty.

RISKS, STRESS, OR DISCOMFORT

Some individuals experience motion sickness with using virtual reality. Using virtual reality is not recommended for individuals with seizure disorders. If you experience discomfort, please stop using the eHBB VR application and inform the study coordinator immediately. All study information will be kept confidential. There is a small risk of loss of confidentiality. However every effort will be made to minimize this risk by using password protection and secure storage of study records. Data collected during the study will be kept indefinitely and deidentified datasets may be shared with other researchers.

For peer review only - http://bmjopen.bmj.com/site/about/guidelines.xhtml

Approved
8/7/2018
UW HSD IRB

BENEFITS OF THE STUDY

Healthcare workers participating in the study may gain added expertise in newborn resuscitation.

SOURCE OF FUNDING

The development of the eHBB and mHBS apps is being supported by the Bill and Melinda Gates Foundation.

CONFIDENTIALITY OF RESEARCH INFORMATION

All of the information you provide will be confidential. Identifier data and observation data will be collected but they will not be linked. Government or university staff sometimes review studies such as this one to make sure they are being done safely and legally. If a review of this study takes place, your records may be examined. The reviewers will protect your privacy. The study records will not be used to put you at legal risk of harm. There are some limits to this protection.

OTHER INFORMATION

You may refuse to participate and you are free to withdraw from this study at any time without penalty or loss of benefits to which you are otherwise entitled.

There are no costs to you for participating in the study. You will be provided with travel costs (if applicable), course attendance fees, and internet data for downloading and using the application. There is no compensation for participating in the study.

RESEARCH-RELATED INJURY

If you have questions, complaints or concerns about this study, you can contact Rachel Umoren at rumoren@uw.edu. The UW does not normally provide compensation for harm except through its discretionary program for medical injury. You do not waive any right to seek payment by signing this consent form.

Printed name of researcher Signature of researcher Date

Subject's statement

This study has been explained to me. I volunteer to take part in this research. I have had a chance to ask questions. If I have questions later about the research, or if I have been harmed by participating in this study, I can contact one of the researchers listed on the first page of this consent form. If I have questions about my rights as a research subject, I can call the UW Human Subjects Division at (206) 543-0098 or call collect at (206) 221-5940. I will receive a copy of this consent form.

Printed name of participant Signature of participant Date

Copies to: Researcher

Participant

For peer review only - http://bmjopen.bmj.com/site/about/guidelines.xhtml

CONSORT 2010 checklist of information to include when reporting a randomised trial*

Section/Topic	Item No	Checklist item	Reported on page No
**Title and abstract**			
	1a	Identification as a randomised trial in the title	1
	1b	Structured summary of trial design, methods, results, and conclusions (for specific guidance see CONSORT for abstracts)	2
**Introduction**			
Background and objectives	2a	Scientific background and explanation of rationale	3
	2b	Specific objectives or hypotheses	4
**Methods**			
Trial design	3a	Description of trial design (such as parallel, factorial) including allocation ratio	4
	3b	Important changes to methods after trial commencement (such as eligibility criteria), with reasons	N/A
Participants	4a	Eligibility criteria for participants	4
	4b	Settings and locations where the data were collected	4
Interventions	5	The interventions for each group with sufficient details to allow replication, including how and when they were actually administered	5-6
Outcomes	6a	Completely defined pre-specified primary and secondary outcome measures, including how and when they were assessed	5-6
	6b	Any changes to trial outcomes after the trial commenced, with reasons	N/A
Sample size	7a	How sample size was determined	7
	7b	When applicable, explanation of any interim analyses and stopping guidelines	N/A
Randomisation:			
Sequence generation	8a	Method used to generate the random allocation sequence	5
	8b	Type of randomisation; details of any restriction (such as blocking and block size)	5
Allocation concealment mechanism	9	Mechanism used to implement the random allocation sequence (such as sequentially numbered containers), describing any steps taken to conceal the sequence until interventions were assigned	5
Implementation	10	Who generated the random allocation sequence, who enrolled participants, and who assigned participants to interventions	5
Blinding	11a	If done, who was blinded after assignment to interventions (for example, participants, care providers, those	5

For peer review only

For peer review only - http://bmjopen.bmj.com/site/about/guidelines.xhtml

		assessing outcomes) and how	
	11b	If relevant, description of the similarity of interventions	N/A
Statistical methods	12a	Statistical methods used to compare groups for primary and secondary outcomes	7-8
	12b	Methods for additional analyses, such as subgroup analyses and adjusted analyses	7-8
**Results**			
Participant flow (a diagram is strongly recommended)	13a	For each group, the numbers of participants who were randomly assigned, received intended treatment, and were analysed for the primary outcome	8
	13b	For each group, losses and exclusions after randomisation, together with reasons	8
Recruitment	14a	Dates defining the periods of recruitment and follow-up	8
	14b	Why the trial ended or was stopped	8
Baseline data	15	A table showing baseline demographic and clinical characteristics for each group	9
Numbers analysed	16	For each group, number of participants (denominator) included in each analysis and whether the analysis was by original assigned groups	8
Outcomes and estimation	17a	For each primary and secondary outcome, results for each group, and the estimated effect size and its precision (such as 95% confidence interval)	9-14
	17b	For binary outcomes, presentation of both absolute and relative effect sizes is recommended	9-14
Ancillary analyses	18	Results of any other analyses performed, including subgroup analyses and adjusted analyses, distinguishing pre-specified from exploratory	9-14
Harms	19	All important harms or unintended effects in each group (for specific guidance see CONSORT for harms)	9-14
**Discussion**			
Limitations	20	Trial limitations, addressing sources of potential bias, imprecision, and, if relevant, multiplicity of analyses	17
Generalisability	21	Generalisability (external validity, applicability) of the trial findings	17
Interpretation	22	Interpretation consistent with results, balancing benefits and harms, and considering other relevant evidence	15-17
**Other information**			
Registration	23	Registration number and name of trial registry	23
Protocol	24	Where the full trial protocol can be accessed, if available	23
Funding	25	Sources of funding and other support (such as supply of drugs), role of funders	23

*We strongly recommend reading this statement in conjunction with the CONSORT 2010 Explanation and Elaboration for important clarifications on all the items. If relevant, we also recommend reading CONSORT extensions for cluster randomised trials, non-inferiority and equivalence trials, non-pharmacological treatments, herbal interventions, and pragmatic trials. Additional extensions are forthcoming: for those and for up to date references relevant to this checklist, see www.consort-statement.org.

For peer review only - http://bmjopen.bmj.com/site/about/guidelines.xhtml

CONSORT 2010 Flow Diagram

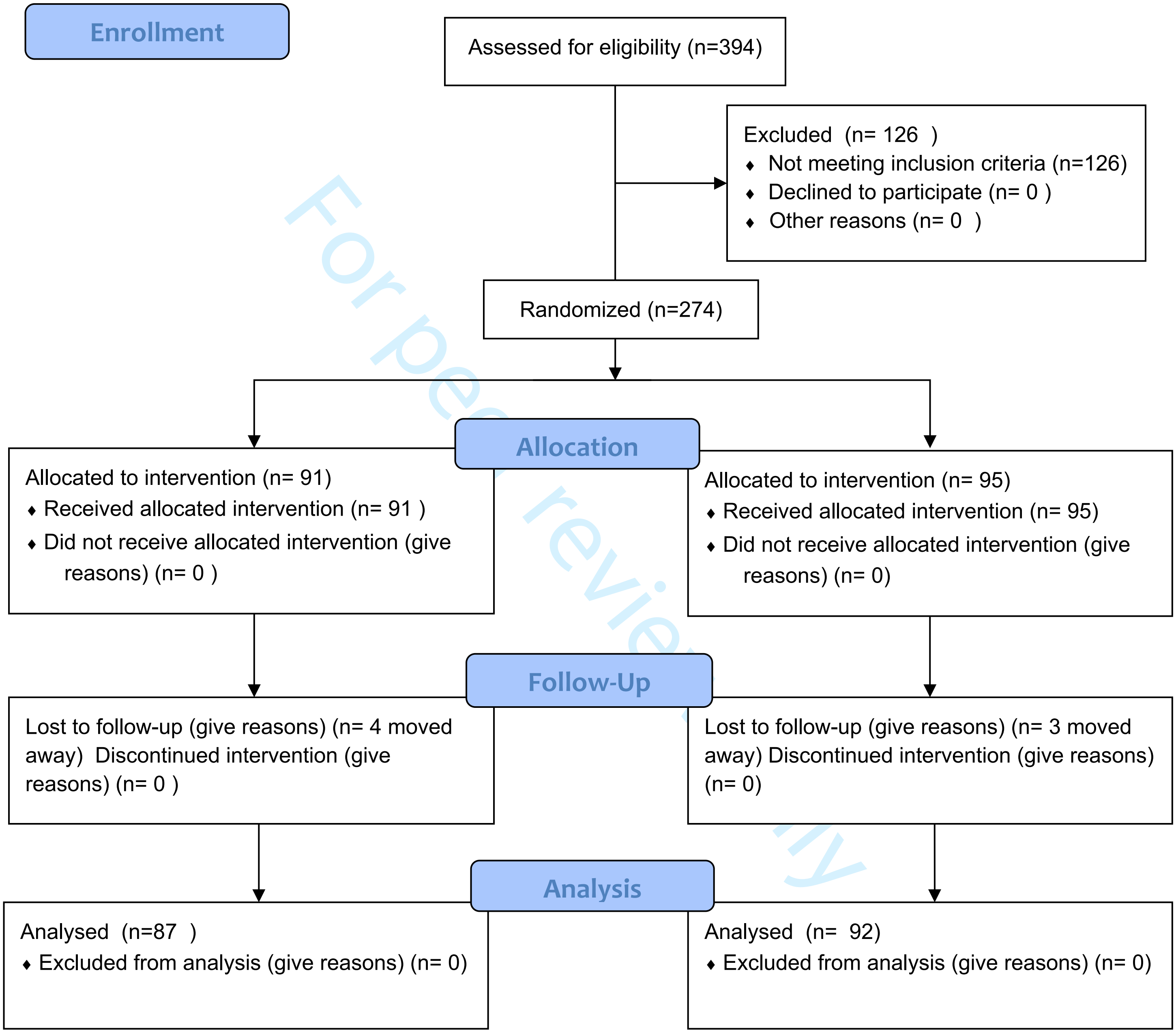

For peer review only
